# Oral rehydration therapies in Senegal, Mali, and Sierra Leone: a spatial analysis of changes over time and implications for policy

**DOI:** 10.1186/s12916-020-01857-7

**Published:** 2020-12-21

**Authors:** Kirsten E. Wiens, Lauren E. Schaeffer, Samba O. Sow, Babacar Ndoye, Carrie Jo Cain, Mathew M. Baumann, Kimberly B. Johnson, Paulina A. Lindstedt, Brigette F. Blacker, Zulfiqar A. Bhutta, Natalie M. Cormier, Farah Daoud, Lucas Earl, Tamer Farag, Ibrahim A. Khalil, Damaris K. Kinyoki, Heidi J. Larson, Kate E. LeGrand, Aubrey J. Cook, Deborah C. Malta, Johan C. Månsson, Benjamin K. Mayala, Ali H. Mokdad, Ikechukwu U. Ogbuanu, Osman Sankoh, Benn Sartorius, Roman Topor-Madry, Christopher E. Troeger, Catherine A. Welgan, Andrea Werdecker, Simon I. Hay, Robert C. Reiner

**Affiliations:** 1 Institute for Health Metrics and Evaluation, University of Washington, 3980 15th Ave. NE, Seattle, WA 98195 USA; 2 Centre for Vaccine Development, Mali (CVD-Mali), Bamako, Mali; 3 African Field Epidemiology Training Programme – Senegal, Ministry of Health, Dakar, Senegal; 4 World Hope International, Makeni, Sierra Leone; 5 Health Care Ministries, Wesleyan Church of Sierra Leone, Makeni, Sierra Leone; 6 Centre for Global Child Health, University of Toronto, Toronto, ON Canada; 7 Centre of Excellence in Women & Child Health, Aga Khan University, Karachi, Pakistan; 8 Department of Global Health, University of Washington, Seattle, WA USA; 9 Department of Health Metrics Sciences, School of Medicine, University of Washington, Seattle, WA USA; 10 Department of Infectious Disease Epidemiology, London School of Hygiene & Tropical Medicine, London, UK; 11 Department of Maternal and Child Nursing and Public Health, Federal University of Minas Gerais, Belo Horizonte, Brazil; 12 ICF International, DHS Program, Rockville, MD USA; 13 Expanded Programme on Immunization, World Health Organization, Geneva, Switzerland; 14 Statistics Sierra Leone, Tower Hill, Freetown, Sierra Leone; 15 Njala University, Njala, Bo, Freetown, Sierra Leone; 16 Faculty of Infectious and Tropical Diseases, London School of Hygiene & Tropical Medicine, London, UK; 17 Institute of Public Health, Jagiellonian University Medical College, Kraków, Poland; 18 Agency for Health Technology Assessment and Tariff System, Warsaw, Poland; 19 Demographic Change and Aging Research Area, Federal Institute for Population Research, Wiesbaden, Germany

**Keywords:** Oral rehydration solution, Recommended home fluids, Oral rehydration therapy, Diarrhea, Health policies, Spatial analysis, Geospatial modeling

## Abstract

**Background:**

Oral rehydration solution (ORS) is a simple intervention that can prevent childhood deaths from severe diarrhea and dehydration. In a previous study, we mapped the use of ORS treatment subnationally and found that ORS coverage increased over time, while the use of home-made alternatives or recommended home fluids (RHF) decreased, in many countries. These patterns were particularly striking within Senegal, Mali, and Sierra Leone. It was unclear, however, whether ORS replaced RHF in these locations or if children were left untreated, and if these patterns were associated with health policy changes.

**Methods:**

We used a Bayesian geostatistical model and data from household surveys to map the percentage of children with diarrhea that received (1) any ORS, (2) only RHF, or (3) no oral rehydration treatment between 2000 and 2018. This approach allowed examination of whether RHF was replaced with ORS before and after interventions, policies, and external events that may have impacted healthcare access.

**Results:**

We found that RHF was replaced with ORS in most Sierra Leone districts, except those most impacted by the Ebola outbreak. In addition, RHF was replaced in northern but not in southern Mali, and RHF was not replaced anywhere in Senegal. In Senegal, there was no statistical evidence that a national policy promoting ORS use was associated with increases in coverage. In Sierra Leone, ORS coverage increased following a national policy change that abolished health costs for children.

**Conclusions:**

Children in parts of Mali and Senegal have been left behind during ORS scale-up. Improved messaging on effective diarrhea treatment and/or increased ORS access such as through reducing treatment costs may be needed to prevent child deaths in these areas.

**Supplementary Information:**

The online version contains supplementary material available at 10.1186/s12916-020-01857-7.

## Background

Oral rehydration solution (ORS) is a type of oral rehydration therapy that can prevent deaths from severe diarrhea and dehydration in children [1]. It is most commonly sold as pre-made packets with standard amounts of sodium and glucose and is properly prepared by dissolving the solution in 1 L of clean water. It has been promoted by the World Health Organization (WHO) and the United Nations Children’s Fund (UNICEF) as an essential medicine to treat diarrhea since it was discovered over 50 years ago [2]. Access to and use of ORS, however, are low globally [3].

Recommended home fluids (RHF) are home-made types of oral rehydration therapy (ORT), which can include sugar-salt solutions, cereal-salt solutions, juice, rice water, tea, or coconut water [1]. In the 1980s, the WHO promoted the use of RHF as home-made alternatives to ORS [2]. However, RHF were broadly defined and it was unclear whether they were effective at preventing diarrheal deaths [1]. Thus, the WHO stopped recommending RHF in 1991 and ORS remains the recommended rehydration treatment for severe diarrhea according to UNICEF, the WHO, and the US Centers for Disease Control and Prevention [4, 5].

In a previous geospatial analysis, we found that the use of ORS increased and the use of RHF decreased between 2000 and 2017 in various low- and middle-income countries (LMICs) at both national and subnational levels [6]. These patterns were particularly striking in subnational areas of Sierra Leone, Mali, and Senegal, countries where diarrhea mortality rates remain high and effective diarrhea treatment is critical to save child lives [7]. It was possible that ORS use replaced RHF use within these countries; alternatively, these trends could indicate some children with diarrhea had been treated with ORS, while others had transitioned from treatment with RHF to no ORT. An important limitation of that analysis was that we did not focus on why there were changes in treatment.

Previous work in these and other countries has additionally shown that ORS scale-up is impacted by factors such as ORS cost, accessibility, knowledge, and local culture [3, 8]. To date, however, no study had specifically examined the relationship between ORS and RHF subnationally temporally in the context of changing health policies and events that impact those factors. Understanding changes in treatment is critical to ending preventable child deaths from severe diarrhea and dehydration.

In order to examine the basis of these trends, we first mapped the use of ORT across second administrative-level units (e.g., districts) in Sierra Leone, Mali, and Senegal to determine if RHF was replaced by ORS, or if RHF was not replaced and hence there were children left completely untreated. Then, in order to understand the broader impact of intervention scale-up on diarrhea treatment and, ultimately, on child survival, we explored whether changes in ORT occurred before or after interventions, changes to healthcare policy, or access. If children were left behind—not yet receiving ORS and no longer receiving RHF—improved access or messaging regarding effective diarrhea treatment may be needed to prevent deaths in affected districts.

Adapting the Bayesian geostatistical methods from our previous study [6], we estimated the percent of children that received three diarrhea treatments (any ORS, only RHF, and no ORT) from 2000 to 2018 at the district/cercle/department level in Sierra Leone, Mali, and Senegal, respectively. To our knowledge, this represents the first spatial analysis of changes in ORT over time in the context of changing policies and access to healthcare.

## Methods

The study locations, data sources, spatial covariates, and statistical analyses for this study are consistent with those employed in our previous study [6], with a few modifications. First, we restricted the analysis to just three countries: Senegal, Sierra Leone, and Mali. We selected these countries because each (1) had experienced changes in ORS and/or RHF during the study period [6], (2) had implemented policies or interventions aimed at improving ORS coverage during the study period (see the “Analysis of policy changes” section below), and (3) had at least 6 years of survey data available. Second, we created mutually exclusive and collectively exhaustive ORT indicators such that each child was placed into exactly one of three categories (any ORS, only RHF, and no ORT). Third, we ran models separately for each country and restricted models to run from the first to the last year of study data. These methods are described below, with further details in Additional file 1 [9–26].

### Survey data

We collected data from national, population-based household surveys—including Demographic Health Surveys and UNICEF Multiple Indicator Surveys—in which primary sampling units (PSU) could be geo-located below the country level. We included data from child modules where mothers were asked whether their children under 5 years of age had diarrhea in the past 2 weeks and, if so, whether they received ORS and/or RHF. In these surveys, diarrhea is defined as three or more loose or watery stools in a 24-h period, which corresponds to acute diarrhea (henceforth referred to as “diarrhea”). In total, we included six surveys in Sierra Leone (years 2000–2017), six surveys in Mali (2001–2018), and nine surveys in Senegal (2000–2017) (Additional file 1: Table S2).

### Treatment categories

We determined the percentage of children with diarrhea in each PSU that received (1) any ORS treatment (“any ORS,” which included treatment with only ORS, or with ORS and RHF), (2) only RHF treatment (“only RHF”), or (3) no oral rehydration treatment (“no ORT,” not treated with ORS or RHF) by taking the population-weighted mean of all children sampled within that PSU.

We employed methods from our previous study to adjust for differences in RHF definitions and survey questions from 2000 to 2018 [6]. In brief, RHF questions were classified as including options of (1) recommended or acceptable home fluids, (2) sugar and salt solutions, (3) other home fluids, and/or (4) other liquid foods. We fitted a logistic regression model to surveys across all LMICs, regressing coverage on definition, country-level fixed effects, and a natural cubic spline on survey year. The global adjustment factor for each non-standard definition (including options other than “recommended or acceptable home fluids”) was the coefficient of the fixed effect for that definition. Coverage reported by non-standard surveys was multiplied by the adjustment factor in logit space (Additional file 1: Tables S3 and S4).

### Statistical analyses

Analyses were carried out using R version 3.5.0. Coverage of any ORS, only RHF, and no ORT were modeled independently using the Bayesian model-based geostatistical framework from our previous ORT study [6]. Similar methodological details are available from additional previous mapping work [27–29]. Briefly, this framework used a hierarchical logistic regression model to predict coverage on a continuous surface, assuming similar coverage in locations closer together in space and time and with similar covariate patterns. Potential non-linear relationships between covariates (Additional file 1: Section 2.4) and coverage were incorporated through stacked generalization [30]. Posterior distributions of all model parameters and hyperparameters were estimated using R-INLA version 19.05.30.9000.30 [31]. Coverage estimates were obtained by taking 1000 draws from the posterior distribution and were adjusted by draw to ensure that the three categories summed to 1 in each location-year:

$$ \mathrm{Any}\ {\mathrm{ORS}}_{\mathrm{adjusted}}=\frac{\mathrm{Any}\ \mathrm{ORS}}{\mathrm{Any}\ \mathrm{ORS}+\mathrm{only}\ \mathrm{RHF}}\times \left(1-\mathrm{no}\ \mathrm{ORT}\right) $$

$$ \mathrm{Only}\ {\mathrm{RHF}}_{\mathrm{adjusted}}=\frac{\mathrm{Only}\ \mathrm{RHF}}{\mathrm{Only}\ \mathrm{RHF}+\mathrm{any}\ \mathrm{ORS}}\times \left(1-\mathrm{no}\ \mathrm{ORT}\right) $$

We calculated population-weighted aggregations of the 1000 draws by country and second administrative-level unit. Mean estimates are reported with 95% uncertainty intervals, which represent the 2.5th and 97.5th percentiles of the 1000 draws. All estimates are reported in Additional file 1: Tables S9–S11 and Additional file 2, with corresponding maps of mean estimates in Additional file 1: Figure S5–S6.

### Analysis of policy changes

We conducted a non-systematic literature search to identify changes in health policies, interventions, or events that may have impacted ORS scale-up within each country during the study period. We initially searched Google and PubMed with broad terms such as “Senegal” and “oral rehydration,” or “Senegal” and “diarrhea treatment.” We reviewed research articles, policy reports, news articles, and their references. Based on the initial findings, we modified and expanded our initial search until we exhausted the information we could find.

We synthesized and discussed the findings with in-country experts, whom we identified through contacts in the Global Burden of Disease Collaborator Network. We created a report for each country that included (1) study methodology, (2) summary of key dates and data sources, and (3) preliminary results. In each interview, we (1) discussed the content of the report, (2) answered any questions they had, and (3) asked questions about whether there were key events and/or additional context surrounding changes in diarrhea treatment and access to healthcare at national or subnational levels that should be taken into account.

For the final analysis, we focused on two major events in each country, which are described below with additional details on all policies, programs, and events in the study period in Additional file 1: Section 5.0. The time periods for analysis were selected based on (1) the date(s) that the policy, intervention, or event occurred; (2) years for which we had data available to inform the estimates; and (3) deliberation among co-authors. For ease of interpretation, we set the time periods to start and end at the mid-point of each year (i.e., July 2).

In Sierra Leone, key events were a national policy implemented in 2010 to make healthcare free for pregnant women, new mothers, and children [32], and the Ebola outbreak from 2014 to 2016 [33] (Additional file 1: Section 5.1). We examined ORT changes during three periods: (1) July 2, 2000–July 1, 2009, before policy changes; (2) July 2, 2009–July 1, 2013, comprising policy changes; and (3) July 2, 2013–July 1, 2017, comprising the outbreak.

In Mali, key events were interventions in southern Mali between 2003 and 2004 that introduced ORS and zinc therapy in Bougouni [34, 35] and established mutual health organizations in Bla and Sikasso [36], and the war in North Mali in 2012 [37] (Additional file 1: Section 5.2). We examined ORT changes from (1) July 2, 2001–July 1, 2004, during intervention implementation; (2) July 2, 2004–July 1, 2011, following the interventions; and (3) July 2, 2011–July 1, 2018, comprising the war.

In Senegal, key events were a national policy implemented in 2008 to promote combined ORS and zinc treatment for diarrhea [38], and a national ORS and zinc intervention launched in 2012 [39, 40] (Additional file 1: Section 5.3). We examined ORT changes from (1) July 2, 2000–July 1, 2006, before policy changes; (2) July 2, 2006–July 1, 2012, comprising policy changes; and (3) July 2, 2012–July 1, 2017, during the intervention.

### Analysis of changes over time

We analyzed changes over time in three distinct ways within each country. First, we described the absolute change in coverage of ORS and RHF within each time period (Additional file 1: Table S10). We compared results by location and year using mean and 95% uncertainty intervals. Second, we examined the annual rate of change in coverage (Additional file 1: Table S11). We calculated these rates at the draw-level to assess whether or not there was a high probability (posterior probability > 95%) that coverage increased or decreased within each second administrative-level unit and time period.

Finally, we examined whether or not the use of RHF was replaced with the use of ORS. RHF was considered not replaced when more than 95% of draws (posterior probability > 95%) showed that decreases in “only RHF” were greater than increases in “any ORS” (i.e., the percent of children that received no ORT increased). RHF was considered replaced when more than 95% of draws showed that the percent of children that received no ORT decreased.

### Numbers of untreated children

Numbers of untreated children with diarrhea were draw-level estimates of treatment coverage at the second administrative-level unit multiplied by mean childhood diarrhea prevalence [7] and by the number of children under the age of 5 [41]. Numbers of children that did not receive any ORS in 2017/2018, but would have received some form of ORT in 2000/2001, were calculated by multiplying the number untreated with ORS in 2017/2018 by ORT coverage (or, 1 – “no ORT”) in 2000/2001.

## Results

### National and subnational (district-level) trends in Sierra Leone

**Fig. 1:**
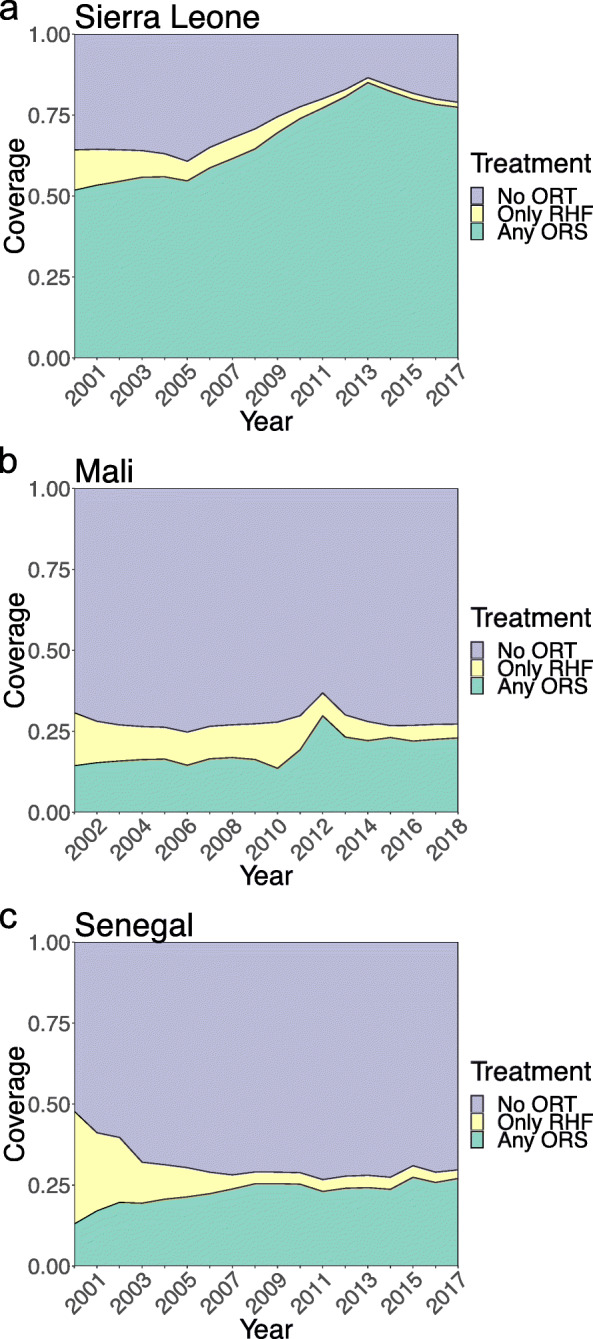
National-level changes in the use of different oral rehydration therapies to treat childhood diarrhea. **a** Changes in Sierra Leone from 2000 to 2017. **b** Changes in Mali from 2001 to 2018. **c** Changes in Senegal from 2000 to 2017. Coverage of any oral rehydration solution (“any ORS,” which included treatment with only ORS or with ORS and RHF) is shown in green; only recommended home fluids (only RHF) is in yellow, and no oral rehydration therapy (no ORT) is in purple. Coverage is defined as the percent of children with diarrhea that fell into each category. Results represent the population-weighted mean of estimates aggregated to the national level. See Additional file 1: Table S9 for corresponding mean estimates and uncertainty intervals

In Sierra Leone, ORS use increased from 51.8% (48.1–55.5) to 77.4% (74.2–80.3) between 2000 and 2017 and exceeded RHF use to treat diarrhea, resulting in a decrease in the percent of children receiving no ORT from 35.8% (32.0–40.0) to 21.1% (18.0–24.2) (Fig. 1a, Additional file 1: Table S9a). Therefore, RHF was completely replaced with ORS at the national level (Fig. 1a).

**Fig. 2:**
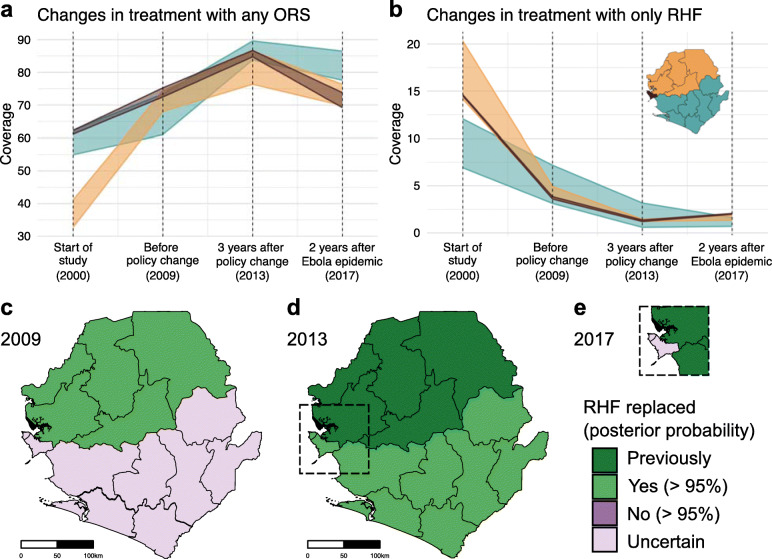
Changes over time in oral rehydration therapies by district in Sierra Leone. **a**, **b** Percent of children that received any ORS (**a**) or only RHF (**b**) at four time points during the study: start of the study (2000); before national policy was implemented to abolish health costs for children, pregnant women, and new mothers (2009); 3 years after policy change (2013); and 2 years after the Ebola epidemic (2017). The western districts saw the greatest number of Ebola cases. Colored bands show the range of the mean estimates across northern districts in orange, southern and eastern districts in blue, and western urban (or Freetown) and western rural districts in brown. Note that in order to clearly illustrate trends in ORS and RHF, scales on the *y*-axis differ between panels **a** and **b**. In addition, in order to focus on changes within three distinct time periods, the *x*-axis is not to scale by year. **c**–**e** RHF replacement by district from 2000 to 2009 (**c**), 2000 to 2013 (**d**), and 2000 to 2017 (**e**). Districts with > 95% posterior probability that RHF was replaced by ORS are shown in green. Light green indicates RHF was replaced by the indicated date, and dark green indicates RHF had already been replaced by the previous date. Districts where our estimates had higher uncertainty (< 95% posterior probability that RHF was either replaced or not replaced) are shown in light purple. Panel **e** corresponds to the region within the dashed inset in panel **d** and illustrates that RHF was no longer replaced in Western districts by 2017

Before a national health policy was implemented in 2010, five districts located in northern Sierra Leone had the lowest ORS coverage in 2000 (ranging from 32.7% (25.3–40.7) to 41.1% (34.8–48.3) coverage) and showed the largest improvements in ORS use by 2009 (55.8% (68.0–78.6) to 74.4% (64.5–82.8)) (Fig. 2a, Additional file 1: Table S10a). Decreases in RHF were small (Fig. 2b) and, by 2009, all northern districts had replaced their RHF use (Fig. 2c). Western, southern, and eastern districts showed increases in ORS use after health costs were abolished for pregnant women, new mothers, and children in 2010 (Fig. 2a). Before the policy, only one of seven southern and eastern districts showed a high probability (posterior probability > 95%) of increased ORS use. In the period comprising the policy change (2009–2013), ORS use increased in all seven southern and eastern districts (Additional file 1: Table S11a), replacing RHF by 2012 (Fig. 2d).

From 2013 to 2017, during and after the 2014–2016 Ebola outbreak, we found country-wide decreases in ORS coverage (Fig. 2a), with high probability of decreases in four of five northern districts, two of two western districts, and one of seven southern and eastern districts (Additional file 1: Table S11a). Sierra Leone’s Western districts saw the greatest decreases, with Western Area Urban (comprising the capital, Freetown) decreasing from 84.8% (79.3–89.1) to 69.2% (60.2–76.7) coverage (Fig. 2a). Western Area Urban also saw the greatest number of Ebola cases in the years 2014 and 2015 [33]. In addition, we found that RHF coverage increased in Western districts in this period, but these increases were smaller than decreases in ORS (Fig. 2b, Additional file 1: Table S11). By 2017, RHF was no longer replaced in Western Area Urban and Rural districts (Fig. 2c).

### National and subnational (cercle-level) trends in Mali

In Mali, ORS use increased nationally from 14.4% (11.8–17.0) to 23.0% (20.5–25.6) between 2001 and 2018 and almost replaced RHF use. The percent of children receiving no ORT changed very little in this time period, from 69.3% (67.0–71.3) to 72.7% (70.3–75.2) (Fig. 1b, Additional file 1: Table S9b). A spike in ORS coverage occurred in 2012 (Fig. 1b), but this should be interpreted with caution as data were not collected in north Mali during the war in 2012.

**Fig. 3:**
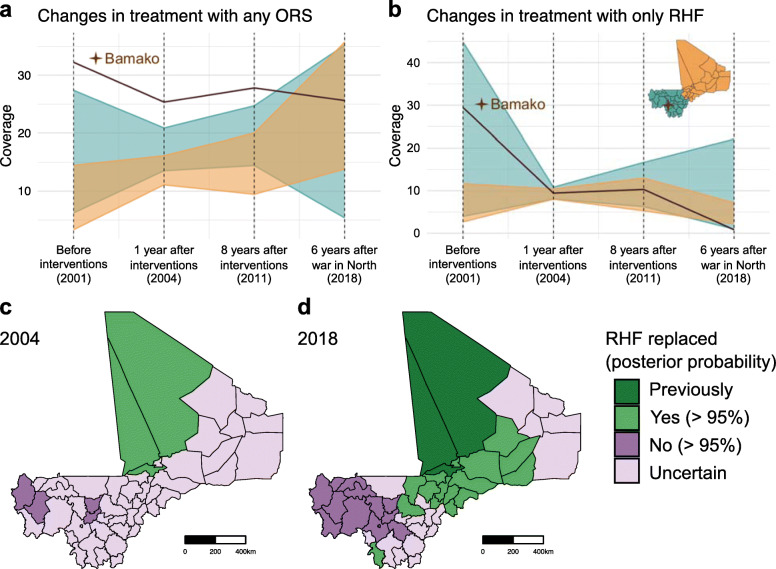
Changes over time in oral rehydration therapies by cercle in Mali. **a**, **b** Percent of children that received any ORS (**a**) or only RHF (**b**) at four time points during the study: before interventions were implemented in south Mali (2001), 1 year after interventions (2004), 8 years after interventions (2011), and 6 years after the war in North Mali (2018). Colored bands show the range of the mean estimates across northern cercles in orange, southern cercles in blue, and the capital city Bamako in brown and highlighted with a star. Note that in order to clearly illustrate trends in ORS and RHF, scales on the *y*-axis differ between panels **a** and **b**. In addition, in order to focus on changes within three distinct time periods, the *x*-axis is not to scale by year. **c**, **d** RHF replacement by cercle from 2001 to 2004 (**c**) and 2001 to 2018 (**d**). Cercles with > 95% posterior probability that RHF was replaced by ORS are shown in green. Light green indicates RHF was replaced by the indicated date, and dark green indicates RHF had already been replaced by the previous date. Cercles where there was greater than 95% posterior probability that RHF was not replaced are shown in darker purple. Cercles where our estimates had higher uncertainty (< 95% posterior probability that RHF was either replaced or not replaced) are shown in light purple. Cercles with > 95% posterior probability that RHF was not replaced by ORS are shown in dark purple

Subnationally, we found very little change in ORS coverage between 2001 and 2004, including cercles in southern Mali where interventions were implemented (Fig. 3a). In Bougouni, ORS coverage changed from 13.7% (8.6–19.8) to 16.5% (4.1–36.8), in Sikasso from 16.7% (10.4–24.5) to 17.2% (3.8–42.6), and in Bla from 21.5% (13.2–31.8) to 15.7% (3.3–38.3) (Fig. 3a). ORS coverage increased in northern Mali during this time period (Fig. 3a), although uncertainty around these estimates was high (Additional file 1: Table S10b). Northern cercles had low ORS coverage in 2001 (ranging from 3.3% (1.1–6.4) to 14.5% (7.2–24.8) coverage), and by 2004 had similar coverage to most southern cercles (11.1% (2.2–27.0) to 16.2% (4.1–40.6)). By 2004, RHF had been replaced in three of 21 northern cercles and had not been replaced in four of 28 southern cercles (Fig. 3c).

In the period following the interventions (2004–2011), we found little change in ORT coverage throughout Mali. Larger changes occurred between 2011 and 2018, which comprised the war (Fig. 3a, b). By 2018, 6 years after the war in north Mali, decreases in RHF (Fig. 3b) were replaced by increases in ORS in 16 of 21 northern cercles and three of 28 southern cercles (Fig. 3d). RHF was not replaced in 12 of 28 southern cercles or in Bamako (Fig. 3d).

### National and subnational (department-level) trends in Senegal

In Senegal, the proportion of children with diarrhea that received ORS increased from 13.0% (10.0–16.6) to 27.1% (23.4–30.7) between 2000 and 2017 (Fig. 1c, Additional file 1: Table S9c). The decrease in the proportion of children that received RHF, however, exceeded the increase in ORS and the proportion of children receiving no ORT increased from 52.3% (48.0–56.6) to 70.3% (66.5–74.0) (Fig. 1c, Additional file 1: Table S9c). Therefore, RHF was not replaced with ORS at the national level (Fig. 1c).

**Fig. 4:**
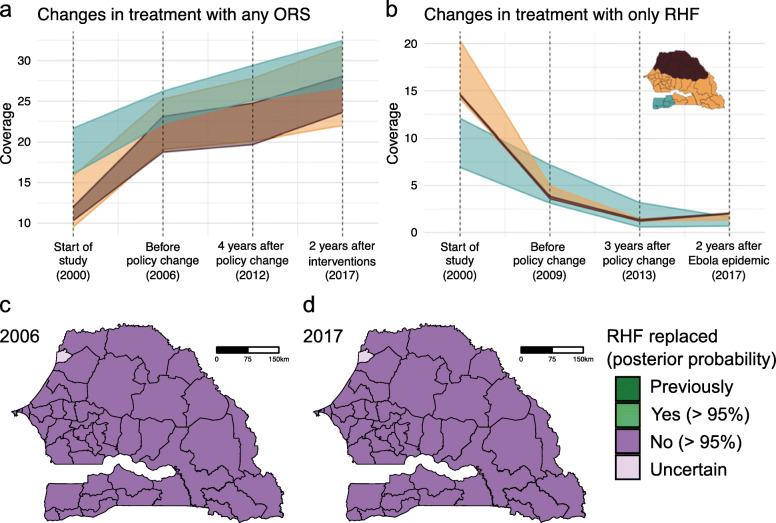
Changes over time in oral rehydration therapies by department in Senegal. **a**, **b** Percent of children that received any ORS (**a**) or only RHF (**b**) at four time points during the study: start of the study (2000), before the policy change to promote improved ORS and zinc as diarrhea treatment (2006), 6 years after the policy change (2012), and 5 years after the launch of the national ORS and zinc scale-up intervention (2017). Colored bands show the range of the mean estimates across northern departments in brown, south-western departments in blue, and the rest of the departments in orange. These divisions were chosen to highlight the largest differences between departments in 2000. Note that in order to clearly illustrate trends in ORS and RHF, scales on the *y*-axis differ between panels **a** and **b**. In addition, in order to focus on changes within three distinct time periods, the *x*-axis is not to scale by year. **c**, **d** RHF replacement by department from 2000 to 2006 (**c**) and 2000 to 2017 (**d**). Departments with > 95% posterior probability that RHF was not replaced by ORS are shown in darker purple. Departments where our estimates had higher uncertainty (< 95% posterior probability that RHF was either replaced or not replaced) are shown in light purple

Most changes in ORT occurred between 2000 and 2006, before promotion and implementation of combined ORS and zinc treatment for diarrhea, and there was limited variation between departments (Fig. 4a, b, Additional file 1: Table S10c). Increases in ORS were small, ranging from between 9.5% (6.1–14.0) and 21.6% (16.1–29.5) coverage in 2000 to between 18.8% (12.4–24.4) and 26.2% (19.2–34.9) coverage in 2006 (Fig. 4a). Decreases in RHF were large, ranging from between 26.4% (18.9–32.4) and 41.3% (32.8–50.6) coverage in 2000 to between 5.0% (2.6–8.3) and 10.7% (5.4–17.8) coverage in 2006 (Fig. 4b). Thus, RHF had not been replaced in any department by 2006 (Fig. 4c).

During the periods comprising the policy change (2006–2012) and the intervention (2012–2017), mean ORS coverage continued to increase while RHF decreased in most departments (Fig. 4a, b). While 28 of Senegal’s 45 departments showed high probability of improvement between 2000 and 2006, however, none showed high probability of improvement after 2006 (Additional file 1: Table S11c). By 2017, RHF had still not been replaced with ORS in 44 of Senegal’s 45 departments (Fig. 4d).

### Numbers of untreated children

**Fig. 5:**
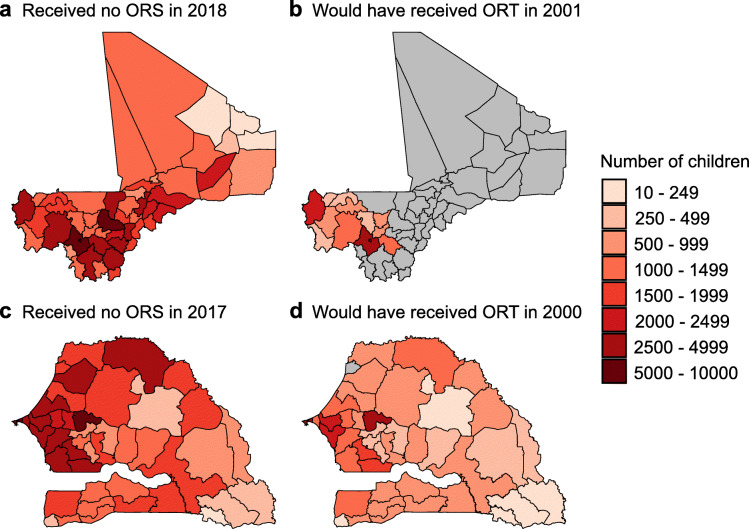
Number of children with diarrhea that were untreated with oral rehydration therapies. **a** Number of children with diarrhea that did not receive any ORS by cercle in Mali in 2018. **b** Number of children in Mali in 2018 that did not receive any ORS, but would have received ORT (ORS and/or RHF) if coverage were at 2001 levels. **c** Number of children with diarrhea that did not receive any ORS by department in Senegal in 2017. **d** Number of children in 2017 in Senegal that did not receive any ORS, but would have received ORT (ORS and/or RHF) if coverage were at 2000 levels. Panels **b** and **d** show units with > 95% posterior probability that RHF was not replaced by ORS; all other units are masked in gray. Numbers of children with diarrhea were determined using previous estimates of mean diarrhea prevalence in 2017 [7]

In the previous analyses, we found that Senegal and Mali each contain areas where decreases in RHF coverage were not replaced with increases in ORS coverage (Figs. 3 and 4) and numbers of untreated children remained high (Additional file 1: Figure S6). In order to estimate the numbers of children that have potentially been left behind during ORS scale-up efforts, we compared the number of children that remained untreated with ORS at the end of the study with the number of children that would have received any form of ORT (ORS and/or RHF) at the beginning of the study (Fig. 5).

In Mali, we found that 87,530 (84,580–90,360) children with diarrhea did not receive ORS in 2018 (Fig. 5a, Additional file 1: Table S9b). Of these, 26,900 (24,280–29,830) would have received some form of ORT if coverage maintained 2001 levels (Fig. 5b). Numbers were highest in the capital of Bamako; in Kati Cercle, Koulikoro Region; and in Kayes Cercle, Kayes Region (Fig. 5b). In Senegal, 77,780 (73,920–81,640) children with diarrhea did not receive ORS in 2017 (Fig. 5c, Additional file 1: Table S9c). Of these, 37,130 (32,100–42,420) would have received some form of ORT if coverage were at 2000 levels (Fig. 5d). Numbers were highest in Pikine Department, Dakar Region; in Mbacké Department, Diourbel Region; and in the capital of Dakar (Fig. 5d). These represent areas where a large number of children may have been left behind during ORS scale-up efforts.

## Discussion

This study shows, for the first time, how ORS and RHF treatments have changed in the context of changing health policies and interventions. This expands on previous findings that Sierra Leone was a “sustained success” in ORS scale-up, while Senegal and Mali were “non-starters” [3].

### Sierra Leone

In Sierra Leone, northern districts—which on average are estimated to have greater travel times to urban centers than southern and eastern districts [42]—had the lowest ORS use in 2000 and showed the greatest increases in ORS from 2000 to 2009. In comparison, southern and eastern districts that had higher ORS coverage in 2000 showed slower progress until 2010, when health costs were abolished for children, pregnant women, and new mothers [32], which increased healthcare utilization [43]. This may reflect a positive effect of low-cost access to healthcare on ORS scale-up.

ORS use to treat diarrhea decreased in northern and western districts during the Ebola outbreak (2014–2016) [33], which decreased healthcare utilization [44]. This change was largest in the capital of Freetown, the only district where RHF use was not replaced with ORS by 2017. Northern and western districts may be more susceptible to changes in access to ORS during disruptions to healthcare, such as the Ebola outbreak. In southern and eastern Sierra Leone, increased access to urban centers [42, 45] and larger amounts of foreign aid after the civil war [46] may have contributed to the relatively higher and more stable ORS coverage, as well as lower diarrhea prevalence [7].

### Mali

In Mali, we found small increases in ORS coverage in the north, which had extremely low coverage rates in 2000. Northern Mali is part of the Sahel desert, a region comprised largely of nomadic groups [47] with limited access to clean water, healthcare, and education, as well as widespread hunger and malnutrition [48]. Along with parts of Mopti in central Mali, it saw massive displacement and violence during the 2012 war [37] and continues to experience frequent conflict. It is unsurprising that diarrhea prevalence is high [7] and ORS coverage is low in a region with such serious security problems.

An additional barrier to ORS scale-up is insufficient access to healthcare throughout Mali. Treatments are generally mediated through community healthcare workers, of which there were only an estimated 2.7 workers per 10,000 people in 2007 [49]. In addition, although ORS is promoted through radio, television, and child survival interventions, it is often only considered with other therapies due to the time and expense of seeking treatment [49] and the knowledge that ORS does not cure diarrhea [35].

### Senegal

In Senegal, we found substantial decreases in RHF, with relatively small increases in ORS, such that many children went untreated with ORT in all districts. From 2000 to 2006—which saw the greatest decreases in RHF—a variety of USAID “technical assistance” programs were implemented across the country to introduce ORS through “health huts” and integrated community care management [38, 50]. The ORS prices were marked up in order to cover supply costs [51], suggesting that these programs increased ORS awareness without commensurately increasing access. Similar results have been found in other intervention contexts. A study on insecticide-treated bednets in Madagascar found that when prices increased by $0.55, coverage fell by 23.1% points [52].

We found no evidence for associations between changes in diarrhea treatment policy [38] or ORS and zinc scale-up programs [39, 40] and changes in ORT in Senegal. Previous work discussed how weak distribution systems, lack of knowledge of ORS, treatment cost, and reliance on external funders have contributed to slow ORS uptake in Senegal [3]. Additionally, about a third of Senegal’s population utilize private pharmacies [53], which may under-prescribe ORS [54]. Moreover, a 2011 study—conducted after a pilot ORS and zinc intervention was implemented in Dakar, Diourbel, and St. Louis—reported that home-made solutions were the preferred treatment for childhood diarrhea [55]. This is in contrast to Sierra Leone’s success, which has been attributed to strong community-based support for ORS treatment since the civil war [3].

### Implications for policy

Our results suggest that national-level policies promoting ORS are necessary but not sufficient for ORS scale-up. Policies must be combined with efforts to make ORS affordable and widely available. Sierra Leone succeeded in combining these strategies, while Senegal implemented a policy without increasing access. The decrease in ORS coverage during the Ebola outbreak in northwestern Sierra Leone and the overall low coverage in Mali additionally highlight the importance of stability for ensuring access.

The importance of free or affordable access to healthcare for ORS scale-up is supported by previous work. A trial in Uganda found that providing free and convenient ORS packets increased coverage by 19% points compared to controls [56]. The governments of Senegal and Mali have each committed to extending free healthcare to pregnant women and children by 2022 [57, 58]. If implemented successfully, these programs could make important progress toward reducing diarrheal deaths in children.

In all three countries, it will continue to be critical to educate caregivers about diarrhea causes, symptoms, and effective treatment. In rural Mali, mothers knew of ORS but sought other cures, such as antibiotics, traditional medicines, or malaria pills [35]. In Mbour, Senegal, only 23.2% of caregivers showed good management of diarrhea [59], while in Dakar, Diourbel, and St. Louis, only 29–54% knew of ORS and zinc treatment [55]. In contrast, 94.1% of mothers in rural Sierra Leone knew of ORS and its use to treat diarrhea [60]. Only 43.9%, however, had overall knowledge of diarrhea management, and only 39.0% knew the procedure for preparing RHF when ORS was not available [60]. In addition, in Sierra Leone, ORS is often used for conditions it is not intended to treat (Carrie Jo Cain, personal communication). This illustrates the complexity in education around diarrhea treatment and the importance of further work in this area.

### Limitations

This study has several limitations. Our models were optimized for prediction, not for causal inference. Thus, our aim was to describe patterns rather than evaluate causal impacts of policies or covariates on treatment changes. In addition, we did not have data in Sierra Leone immediately after the Ebola outbreak in 2015 and 2016, in Senegal from 2001 to 2005, or in northern Mali in 2012, so we were unable to capture data-driven variation in these years. Data quality also varied between surveys and locations. For example, in Mali, there was greater heterogeneity in the data compared with Senegal and Sierra Leone. This led to comparatively lower predictive validity (Additional file 1: Figure S2-S4), higher uncertainty (Additional file 1: Tables S9-S11), and many locations where we could not confidently estimate whether or not RHF was replaced with ORS (Fig. 3c, d). For this reason, we focused the final analysis of numbers of children left behind on subnational locations where uncertainty in our estimates was lower (Fig. 5). Though, in this latter analysis, we could not propagate uncertainty for numbers of children with diarrhea [7, 41]. Finally, survey data reported ORS and RHF use to treat diarrhea, but we cannot make conclusions about effective coverage, such as whether the solutions were prepared correctly. Similarly, the survey data did not include symptoms stratified by disease severity and we were not able to evaluate whether it was used appropriately to treat severe diarrhea and dehydration.

### Future work

Additional studies are needed on how health system functionality, treatment supply, and co-coverage of other interventions are associated with effective diarrhea treatment and reductions in child deaths. Our previous work showed that annualized rates of decline in childhood diarrhea mortality were similar in Sierra Leone, Senegal, and Mali (with high levels of uncertainty) [7]. Studies that develop inferential approaches, and integrate new data sources, could examine how interventions other than ORS have driven declines in Mali and Senegal and the best ways to reduce mortality further in Sierra Leone.

Similarly, in this study, we were not able to examine how subnational patterns in ORS coverage compared with patterns in joint ORS-zinc treatment. UNICEF’s latest data show that ORS-zinc treatment for diarrhea has increased nationally in Senegal from 0% (2011) to 18% (2017), in Mali from 2% (2012) to 9% (2018), and in Sierra Leone from 3% (2013) to 43% (2017) [61]. As more data become available and additional countries adopt policies promoting joint ORS-zinc therapy, mapping ORS-zinc coverage will be an important extension of this work.

Finally, these analyses could be applied to additional diseases and interventions. Subnational estimates of lower respiratory infections, malaria, HIV, and other health risks are publicly available and can be explored using interactive online visualization tools [62]. We encourage other investigators to use these data for similar retrospective studies.

## Conclusions

Our results show that districts with low ORS coverage in Mali, Sierra Leone, and Senegal have tended to quickly catch up with districts of higher coverage. Significant health policy changes such as abolishing health costs, however, may be needed to increase coverage further. We also show that events such as conflict and the Ebola outbreak likely negatively impact ORS access. Effective messaging regarding appropriate diarrhea treatment could save child lives during these destabilizing events, as well as in locations where ORS has not yet replaced RHF. These expanded efforts will be critical to reach the over 150,000 children with diarrhea that do not receive ORS in Mali and Senegal.

## Supplementary Information

**Additional file 1: Supplementary appendix.** Document containing detailed description of case definitions, data, geostatistical model, model validation, policies and events in each country, Tables S1-S11, and Figure S1-S6.

**Additional file 2.** Supplementary estimates for second administrative level-units. Spreadsheet containing all estimates of any ORS, only RHF, and any ORT coverage by district from 2000 to 2017 in Sierra Leone, by cercle from 2001 to 2018 in Mali, and by department from 2000 to 2017 in Senegal.

## Data Availability

The datasets supporting the conclusions of this article are included within the article and its additional files. The source code used to generate estimates and perform analysis is available at https://github.com/ihmeuw/lbd/tree/ort-sen-mli-sle-2020. The study data, including full sets of estimates at second administrative levels, are available at http://ghdx.healthdata.org/record/ihme-data/senegal-mali-and-sierra-leone-oral-rehydration-therapy-geospatial-estimates-2000-2018.

## Abbreviations

LMICs: Low- and middle-income countries
ORS: Oral rehydration solution
ORT: Oral rehydration therapy
PSU: Primary sampling unit
RHF: Recommended home fluids
UNICEF: United Nations Children’s Fund
WHO: World Health Organization
