# Supplementary material for: Oral rehydration therapies in Senegal, Mali, and Sierra Leone: a spatial analysis of changes over time and implications for policy
Source: BMC Med. 2020 Dec 21;18:405. doi: 10.1186/s12916-020-01857-7 (PMC7750121; doi:10.1186/s12916-020-01857-7)
Supplement: Supplementary file 1 — Additional file 1: Supplementary appendix. Document containing detailed description of case definitions, data, geostatistical model, model validation, policies and events in each country, Tables S1-S11, and Figure S1-S6. [file 12916_2020_1857_MOESM1_ESM.pdf]

Oral rehydration therapies in Senegal, Mali, and Sierra Leone: a spatial analysis of changes over time and implications for policy

Additional file 1: Contents

0.0 GATHER compliance ... 2
1.0 Case definition of modelled outputs ... 2
- **1.1 Any oral rehydration solution ('any ORS')** ... 2
- **1.2 Only recommended home fluids ('only RHF')** ... 2
- **1.3 No oral rehydration therapy ('no ORT')** ... 2

2.0 Data ... 3
- **2.1 Summary of included data sources** ... 3
- **2.2 Aggregation to finest possible geography** ... 3
- **2.3 Creation of pseudo-points within areal units** ... 3
- **2.4 Assigning covariates to points** ... 3
- **2.5 Administrative boundaries** ... 3

3.0 Geostatistical model ... 4
- **3.1 Model geographies and years** ... 4
- **3.2 Ensemble covariate modelling via stacked generalisation** ... 4
- **3.3 Geostatistical model** ... 4
- **3.4 Priors** ... 5
- **3.5 Mesh creation** ... 5
- **3.6 Fitted parameters and estimate generation** ... 6

4.0 Model vetting and validation ... 6
- **4.1 Vetting stacker models and time trends** ... 6
- **4.2 In-sample validation** ... 6
- **4.3 Out-of-sample validation** ... 6

5.0 Policy changes, interventions, and events ... 7
- **5.1 Sierra Leone** ... 7
- **5.2 Mali** ... 8
- **5.3 Senegal** ... 9

6.0 Additional file 1: Figures ... 10
7.0 Additional file 1: Tables ... 21
8.0 Additional file 1: References ... 61

0.0 GATHER compliance

Please see Additional file 1: Table S1 for discussion on how this study meets the conditions for GATHER guidelines.

1.0 Case definition of modelled outputs

1.1 Any oral rehydration solution (‘any ORS’)

The ‘any ORS’ category corresponded to children who received only ORS as well as children who received both ORS and RHF (see below). ORS was defined as a pre-packaged electrolyte solution containing glucose or another form of sugar or starch, as well as sodium, chloride, potassium, and bicarbonate or citrate. This included reduced osmolarity ORS (total osmolarity ≤250 mmol/l) and higher osmolarity ORS (up to 370 mmol/l) (1). Diarrhea was defined as a child having had three or more abnormally loose or watery stools within a 24-hour period.

The modelled output was the *proportion of children under 5 years-old (under-5) with diarrhea that received any ORS*, and corresponded to the following survey questions that were asked about children who had diarrhea in the previous 2 weeks (14 days):

- Did child receive a special packet called [local name for solution]?
- Did child receive a pre-packaged ORS liquid?
- This included any pre-made ORS solution, with designations such as *“oral rehydration solution”, “ors”, “sro”, “serum from pharmacy”, “soro”, “suero”, “pre-packaged”, “special packet”, “pedialyte”*.

1.2 Only recommended home fluids (‘only RHF’)

The ‘only RHF’ category corresponded to children who received RHF but did not receive any ORS. RHF were defined as all possible home fluid alternatives, including but not limited to sugar-salt solution, cereal-salt solution, rice-water solution, and additional fluids, such as plain water, juice, tea, or rice water. The only solution that is consistently not included in the “recommended fluid” definition is soda (1).

The modelled output was the *proportion of children under-5 with diarrhea that received only RHF*, and corresponded to the following survey questions that were asked about children who had had diarrhea in the previous 2 weeks (14 days):

- Did child receive government-recommended home fluids?
- Did child receive any other fluid-based treatment [written in or listed]?
- This includes any home fluid that may increase hydration, with designations such as *“recommended home solution”, “suero casero”, “sugar and salt solution”, “cereal-water”, “sweetened”, “salted”, “rice water”, “soup”, “fruit juice”, “yoghurt-based drink”, “tea”*.

Given the broad range in RHF definitions between countries, in addition to the fact that survey questions change over time and between survey series, we performed an analysis to adjust all “non-standard” RHF survey questions to a “standard” question. RHF questions were classified as including phasing that fell into categories of 1) recommended or acceptable home fluids, 2) sugar and salt solutions, 3) other home fluids, and/or 4) other liquid foods, for a total of 16 different possible definition combinations. The “standard” questionnaires were ones that asked only about category 1, recommended or acceptable home fluids, which was found most frequently across all surveys. We fit a logistic regression model to surveys across all low- and middle-income countries (LMICs) in the analysis, using data and methods from our previous study (2), regressing coverage on definition, country-level fixed effects, and a natural cubic spline on survey year. Coverage reported by non-standard surveys was adjusted by the coefficient of the fixed effect for the non-standard definition in logit space. See Additional file 1: Table S3 for the resulting RHF coverage adjustments.

1.3 No oral rehydration therapy (‘no ORT’)

The ‘no ORT’ category corresponded to all children that did not fall into either of the above categories (i.e., that did not receive ORS or RHF). ORT was defined as any form of oral rehydration treatment, including treatment with either ORS, RHF, or both. The modelled output was the *proportion of children under-5 with diarrhea that received*

neither ORS nor RHF. It was modelled using the ORS and RHF survey data described above. It was also adjusted for differences in RHF survey questions using the methods described in section 1.2. See Additional file 1: Table S4 for the resulting ORT coverage adjustments.

2.0 Data

2.1 Summary of included data sources

We collected data from national, population-based household surveys in Mali, Senegal, and Sierra Leone. For a survey to be considered for this analysis, we required that it provide geography information more granular than the national level, had data collected during the time frame of 2000 to 2018, provided survey weights if the survey was not self-weighted, provided data on whether a child had diarrhea in the weeks prior to the survey and data on whether children who had diarrhea had received ORS and RHF as treatment. This included 6 surveys in Sierra Leone from 2000 to 2017, 6 surveys in Mali from 2001 to 2018, and 9 surveys in Senegal from 2000 to 2017 (Additional file 1: Table S2).

2.2 Aggregation to finest possible geography

We aggregated/summarized the individual-level microdata to the finest possible spatial resolution available—preferably, a latitude and longitude pair representing the location of the survey cluster/primary sampling unit. Where point-level referencing was not available, we matched survey microdata to the smallest polygon/areal unit possible. We calculated the effective sample size for each spatial aggregation (point and polygon) via the Kish approximation considering the underlying complex survey design (3). After aggregation, the adjustments described below (sections 2.3–2.4) were applied.

2.3 Creation of pseudo-points within areal units

We created pseudo-points for areal data via a population-weighted resampling process as our desired model requires data of a single geometric type (e.g., latitude/longitude point). Specifically, we randomly generated 10 000 candidate points from within each areal unit using the WorldPop total population raster as a spatial distribution weight (4). K-means clustering was performed to aggregate candidate points into the pseudo-points used for modelling. These pseudo-points were assigned analytical weights proportional to the number of candidate points that entered into the k-means cluster. Each pseudo-point generated by this process was assigned the ORS coverage observed from the survey for that polygon.

2.4 Assigning covariates to points

We compiled 15 covariates that were indexed at the subnational level and had conceivable relationships with ORT: Access to cities, ratio of children dependents (age 0 to 14) to working adults (age 15 to 64), distance from rivers or lakes, night-time lights^{TV}, elevation, number of children under 5 per woman of childbearing age, total population^{TV}, aridity^{TV}, urban or rural^{TV}, urban proportion of the location^{TV}, irrigation, number of people whose daily vitamin A needs could be met, prevalence of under-5 stunting^{TV}, prevalence of under-5 wasting^{TV}, and maternal education^{TV} (TV = time-varying covariates). We also included the Healthcare Access and Quality Index (5) and the proportion of pregnant women who received four or more antenatal care visits as national-level time-varying covariates. We filtered these covariates for multi-collinearity within each country using variance inflation factor (VIF) (6) analysis using a threshold of VIF < 3. Additional file 1: Table S5 lists the source information for the covariates and Additional file 1: Table S6 shows the final covariates selected for each region following VIF analysis. Once assembled, we conducted a spatial query to match covariate values spatially and temporally to our collection of points and pseudo points. For numerical stability, all covariates were centered and scaled to mean 0, with a standard deviation of 1.

2.5 Administrative boundaries

For this analysis we use shapefiles from the Database of Global Administrative Areas (GADM) (7) to define relevant country and subnational/administrative boundaries. We made slight adjustments to ensure proper nesting of

administrative units, and made larger adjustments in the Democratic Republic of the Congo and India where collaborators in these countries had indicated mistakes in administrative boundaries in the shapefiles.

3.0 Geostatistical model

3.1 Model geographies and years

We ran models separately for Mali, Senegal, and Sierra Leone. In addition, we ran models from the first year of data to the last year of data, corresponding to 2000 to 2017 for Senegal and Sierra Leone and 2001 to 2018 for Mali. This ensured that our estimates were anchored in data and prevented the need to interpolate forward or backward in time. An overview of the modelling strategy can be found in Additional file 1: Figure S1.

3.2 Ensemble covariate modelling via stacked generalisation

We used a stacked generalisation ensemble model framework to capture non-linear effects and complex interactions among our covariates (8). For each region (see section 3.1), we fit three child models to our dataset: a generalised additive model (GAM), a penalised regression with the elastic net penalty, and a boosted regression tree (BRT). As described below in section 3.3, we use a spatio-temporal Gaussian process regression as the parent ensembler.

Parameters for the GAM model (spline type and number of knots) were selected by expert priors with a maximum of 3 knots, and the lambda parameter for the elastic net regression was selected by cross validation. Initial hyperparameters for the BRT (namely tree complexity, learning rate, and number of trees) were selected using non-parametric Bayesian optimisation over a finite space, where the objective function was the negative mean absolute error of the BRT fit (9). See Additional file 1: Table S8 for all initial hyperparameters that were selected by modelling region.

Each child model was fit using five-fold cross validation to reduce overfitting, and the out-of-sample predictions across the child model hold-outs were compiled into a single set of model predictions. Additionally, each child model was fit on 100% of the data and a full set of in-sample predictions were created. The out-of-sample predictions per child model were fed to the parent geostatistical model (see section 3.3 below) as covariates for fitting while the in-sample predictions from the child models are used during the parent model's prediction step.

3.3 Geostatistical model

Binomial count data are modelled within a Bayesian hierarchical modelling framework using a logit link function and a spatially- and temporally-explicit hierarchical generalised linear regression model to estimate the point prevalence (or coverage) of any ORS, only RHF, or no ORT in each modelling region (see section 3.1). Our model was constructed as follows:

$$C_i | p_i,\ N_i \sim \text{Binomial}(p_i,\ N_i)$$

$$\text{logit}(p_i) = \beta_0 + \boldsymbol{X_i \beta} + \epsilon_{GP_i} + \epsilon_{ctry_i} + \epsilon_{study_i} + \epsilon_i$$

$$\sum \boldsymbol{\beta} = 1$$

$$\epsilon_i \sim \text{N}(0, \sigma^2_{nug})$$

$$\boldsymbol{\epsilon_{GP}} | \boldsymbol{\Sigma}_{\text{space}},\ \boldsymbol{\Sigma}_{time} \sim \text{GP}(0,\ \boldsymbol{\Sigma}_{\text{space}} \otimes \boldsymbol{\Sigma}_{time})$$

$$\boldsymbol{\Sigma}_{\text{space}} = \sigma^2 \frac{2^{1-\nu}}{\Gamma(\nu)} \times \left(\frac{\sqrt{8}}{\rho_s}\boldsymbol{D}\right)^{\nu} \times \text{K}_{\nu}\left(\frac{\sqrt{8}}{\rho_s}\boldsymbol{D}\right)$$

$$\Sigma_{time_{j,k}} = \rho^{|t_k - t_j|}.$$

For each region, we modelled the number of under-5 children at location-time i, among a sample size, N_i, with diarrhea who received any ORS, only RHF, or no ORT, respectively, as binomial count data, C_i. The counts, C_i, probabilities, p_i, predictions from the three child models $\boldsymbol{X_i}$, and residual terms ϵ_* are all indexed at a space-time coordinate. The term p_i represents both the annual proportion and the annual probability that an individual child will receive any ORS, only RHF, or no ORT, respectively, given the child resides at that particular location. The logit of annual coverage, $\text{logit}(p_i)$, was modelled as a linear combination of the three child models, $\boldsymbol{X_i}$; a correlated spatio-temporal error term, ${\epsilon_{GP}}_i$; and an independent error term, ϵ_i. Coefficients, $\boldsymbol{\beta}$, on the child models represent their respective predictive weighting in the mean logit link and are constrained to sum to one. The country random effect was not used in individual country models, with the exception of India where we set this term to be state-level random effects. ϵ_{GP}, is modelled as a three-dimensional Gaussian process in space-time centered at zero and with a covariance matrix constructed from a Kroenecker product of spatial and temporal covariance kernels. The spatial covariance, Σ_{space}, is modelled using a Matérn covariance function (10), and temporal covariance, Σ_{time}, as an autoregressive order 1 (AR1) function represented in the model with 18 annual knots.

This approach leveraged the data's residual correlation structure to more accurately predict coverage estimates for locations with no data, while also propagating the dependence in the data through to uncertainty estimates (11). The posterior distributions were fit using computationally efficient and accurate approximations in R-INLA (12,13) (integrated nested Laplace approximation) with the stochastic partial differential equations (SPDE) (14) approximation to the Gaussian process residuals.

3.4 Priors

The following priors were used:

- $\beta_0 \sim N(\mu = 0, \sigma^2 = 1000)$,
- $\boldsymbol{\beta} \sim N(\boldsymbol{\mu}, \Sigma\ )$,
  - $\boldsymbol{\mu} = (\frac{1}{3}, \frac{1}{3}, \frac{1}{3})'$
  - $\Sigma\ = 1{,}000 * I_{3\,x\,3}$
- $log\left(\frac{1+\rho}{1-\rho}\right) \sim N(\mu = 2, \sigma^2 = 1/(1.2^2))$,
- $\left(\frac{1}{\sigma^2_{country}}\right) \sim gamma(\alpha = 4, \gamma = 4)$,
- $\left(\frac{1}{\sigma^2_{study}}\right) \sim gamma(\alpha = 1, \gamma = 0.00005)$,
- $\left(\frac{1}{\sigma^2_{nugget}}\right) \sim gamma(\alpha = 1, \gamma = 0.00005)$,

We used a "penalized complexity" prior (15) for the marginal standard deviation, σ, and spatial range, ρ_s, of the spatio-temporal Gaussian process, $\boldsymbol{\Sigma}_{\text{space}}$. The priors for these parameters were specified such that $\Pr(\sigma > 3) = 0.05$ and $\Pr(\rho_s < X) = 0.05$, where X was 5% of the max extent of the mesh for each modelling region (X corresponded to 0.76 in Sierra Leone, 1.42 in Mali, and 0.91 in Senegal).

The mean (μ) and variance (σ^2) parameters for the hyperpriors selected by INLA for the meshes in each region can be found in Additional file 1: Table S5. In our parameterisation we represent α and γ in the *gamma* distribution as rate and shape, respectively. The starting set of hyperparameters were selected using INLA defaults, as well as a previously performed sensitivity analysis (16). From this initial set, we modified the prior on the variance of the country random effects to allow high levels of variation between countries due to the strong effect that national-level policies may have on ORS and RHF coverage.

3.5 Mesh creation

We constructed the finite elements mesh for the stochastic partial differential equation approximation to the Gaussian process regression using a simplified polygon boundary (in which coastlines and complex boundaries were smoothed) for each of the regions within our model. This paper uses an improved mesh that is constructed on the S2 domain. This allows distance to be calculated along the sphere instead of using Euclidean distance between latitude and longitude coordinates. This mesh also generates denser vertices in data rich areas. We set the minimum triangle

edge length to 25 kilometres, the maximum triangle length to 1 000 kilometres, with the mesh extending 500 kilometres past the region's boundary.

3.6 Fitted parameters and estimate generation

Fitted parameters and hyperparameters, as well as their 95% uncertainty intervals are shown by indicator and region in Additional file 1: Table S7. All estimates were generated by taking 1 000 draws from the posterior distribution. For estimates at the 5 × 5-km grid-cell level, these draws were used directly to generate estimates and uncertainty. We generated 95% uncertainty intervals around the mean of our estimates by taking the 2.5% and 97.5% quantiles of each of the draws, at the grid-cell or administrative level. The proportion of children that received any ORS and only RHF estimates were adjusted by draw using the following formulas to ensure that they together summed to 1; the proportion of children that received no ORT in each location-year as follows:

$$any\ ORS_{adjusted} = \frac{any\ ORS}{any\ ORS + only\ RHF} * (1 - no\ ORT)$$

$$only\ RHF_{adjusted} = \frac{only\ RHF}{only\ RHF + any\ ORS} * (1 - no\ ORT)$$

To aggregate our results to second administrative-level units (e.g., districts, counties) for each draw, we fractionally assigned each grid cell to any intersecting unit by examining the starting area of the grid cell and the relative areas of the resulting geometric intersections, and took population-weighted averages of grid cells assigned to each unit. To aggregate to first administrative-level units (e.g., states, provinces), we computed population-weighted averages of nested second administrative units. To aggregate to country levels, we computed population-weighted averages of nested first administrative units.

4.0 Model vetting and validation

4.1 Vetting stacker models and time trends

For each intermediate model and for final models, we created line plots of our estimates for each of the stacking models and the final INLA model including uncertainty overlaid on the input data. We created and reviewed these plots for each country and for each first administrative unit.

4.2 In-sample validation

To explore the in-sample validity of our models, we plotted our predictions vs. the observed data by modelling regions and by year at the country-level, first administrative-level, and second administrative-level aggregations. We also calculated mean error (ME, or bias), root-mean-squared-error (RMSE, which summarizes total variance), and 95% coverage of our predictive intervals (the proportion of observed in-sample data that fall within our predicted 95% uncertainty intervals). The in-sample fit statistics are shown in Additional file 1: Figures S2–S4 and Additional file 1: Table S8 for any ORS, only RHF, and no ORT models.

4.3 Out-of-sample validation

We examined the predictive validity of our modelling strategy using five-fold out-of-sample cross-validation. Folds were created by randomly assigning entire second administrative units, stratified by region, to one of five folds. For each modelling region, we ran the entire modelling process once per fold, in addition to the full in-sample runs described above, generating a complete set of out-of-sample predictions. Using these out-of-sample predictions, we then calculated mean error (ME, or bias), root-mean-squared-error (RMSE, which summarizes total variance), and 95% coverage of our predictive intervals (the proportion of observed out-of-sample data that fall within our predicted 95% uncertainty intervals) aggregated to the spatial holdout level. Additional file 1: Figures S2–S4 show out-of-sample prediction vs. observed data. Additional file 1: Table S8 summarize out-of-sample statistics for any ORS, only RHF, and no ORT models.

5.0 Policy changes, interventions, and events

The sections below include various policy changes, interventions, and events that may have impacted access to healthcare and diarrhea treatment within each country. The events and data sources were collected according to the methods described in the "Analysis of Policy Changes" section within the manuscript methods. Key events in the analysis are bolded and underlined, and events are organized within the time periods that were used for analysis.

5.1 Sierra Leone

2000 to 2009

- Sierra Leone Civil War from 1991 – 2002 (17).
- Before the war, ORS was promoted by Blue Flag Volunteers. During the war, ORS was given in displaced persons camps. After the conflict, ORS continued to be supported and promoted at all levels, from community health workers to the Ministry of Health and Sanitation (18).
- In 2005, began deploying community case management (CCM) through community health volunteers in rural areas to increase coverage of treatment for malaria, pneumonia, and diarrhea (19).

2009 to 2013

- **Key event:** In April 2010, the government of Sierra Leone abolished healthcare costs—including drug and treatment costs—for pregnant women, new mothers and children under five (20,21). Although there were various challenges to full implementation, including inadequate infrastructure and inefficient drug distribution systems (21), the program resulted in increased healthcare utilization (19,21,22).
- In 2010, UNICEF engaged with partners to introduce CCM for diarrhea, malaria, and pneumonia in districts with especially poor health indicators (23).
- In June 2012, the Ministry of Health and Sanitation released a policy for community healthcare workers (CHW) in an effort to strengthen health care system. It described a framework for training, although no specific modules were provided (24).
- In November 2012, UNICEF and World Hope International entered into an agreement to launch a CHW program in Bombali District (which includes Makeni, Sierra Leone's fourth largest city). The aim was improving maternal health outcomes through intensive case management of pregnant women. The agreement covered cost of CHW salaries until the completion of the grant in June 2016 (25).
- In 2012, there was a nation-wide cholera epidemic during the rainy season, Sierra Leone's largest recorded cholera outbreak, with a total of 245 293 cases (26).

2013 to 2017

- **Key event:** Ebola outbreak from 2014 to 2016 (27). Ebola cases were first reported in the spring of 2014. Incidence rose sharply from August to December 2014, exceeding the capacity of Ebola holding and treatment centers (27). In response, additional treatment, holding, and community care centers were opened. This coincided with efforts to increase community engagement and prevent viral transmission, particularly during funerals (27). There were over 8000 confirmed cases of Ebola by January 2015, with the Western Areas seeing the most cases, followed by Port Loko, Bombali, Kailahun, Kenema, Tonkolili, and Bo (27). A study conducted in Moyamba district (Southern Region) and Koinadugu district (Northern Region) found that the outbreak was accompanied by disruptions in relationships between health systems and communities, which led to decreases in health facility utilization, including decreases in both paediatric and maternal admissions (28).

5.2 Mali

2001 to 2004

- **Key event:** From 2003-2004 pilot interventions were conducted in southern Mali that introduced zinc therapy in addition to ORS treatment for diarrhea in two health zones of Bougouni (29,30). Zinc was provided through existing community health centers and drug kits and managed by community health workers. The cost of zinc to the patients was 100 CFA Francs (~0.19 US$) for the full package of 14 zinc tablets, and it was promoted through village meetings and individual counseling at health centers. In addition, health staff were trained in diarrhea case management with zinc and ORS, as well as in diarrhea prevention. Household surveys were conducted before and after introduction. Preliminary evidence suggested that zinc introduction did not affect ORS coverage and that there were various barriers to scale-up, including financial access, management of concurrent diarrhea and fever symptoms, and use of unauthorized drug vendors (29,30).
- **Key event:** From 2003-2004 the Ministry of Health and the Partners in Health Reform project partnered to do a case-control study of mutual health organizations in two rural districts of Bla and two urban districts of Sikasso (31). Mutual health organizations are community-based health insurance services where membership is voluntary. Their aim is to increase healthcare access by reducing out-of-pocket costs of care for individual households. The study found that cases were three times as likely as controls to take children to healthcare facilities and/or treat them with ORS at home (31).

2004 to 2011

- Community-Led Total Sanitation was introduced in 2009 (32).
- In 2011-2015, the National Department of Health set "The Strategic Plan for the promotion of hygiene practices for big impact within the framework for the reduction of diarrhoeal diseases*"* (32).
- The Maternal and Child Health Integrated Program rolled out a mentorship program from October 1, 2010 – June 30, 2014 (33). In this program, trained supervisors coached staff on applying the Integrative Community Care Guidelines for treating diarrhea. This covered 25% of regions, included 7 districts, and a total of 166 facilities (33).

2011 to 2018

- **Key event:** From January to April 2012, Mali underwent its fourth Tuareg uprising since it gained independence from France in 1960 (34). The rebellion was launch on January 17th, three years after the previous north-south peace agreement, with an attack on a Malian military garrison in the north-eastern town of Menaka (34). On March 22, there was an improvised military coup in the south, causing the situation to deteriorate further (34). By the end of April, all major northern cities were controlled by the rebellion. From April to May the rebellion underwent an "Islamist take-over", which led to the establishment of a new order and creation of an Islamic police force in the north (34). The northern region and parts of Mopti in central Mali saw massive displacement and violence during the war (34) and continue to experience frequent conflict.
- In 2019, the government of Mali announced wide-scale health reforms that would include free health services for pregnant women and children under the age of 5, although these reforms will not be fully effective before 2022 (35).

5.3 Senegal

2000 to 2006

Prior to 2006 several USAID "technical assistance" programs were implemented that promoted ORS and RHF treatments (18,36,37):

- PRITECH introduced ORS in Senegal in 1985. ORS was donated by USAID and UNICEF, and was distributed free of charge. PRITECH additionally promoted the use of RHF for diarrhea (18,36,37).
- BASICS followed PRITECH in 1994 and continued to promote both ORS and RHF, and focused on technical assistance in Thiès, Louga, Kaolack, and Ziguinchor regions (18,36,37).
- ORS and RHF were introduced in community "health huts" starting in the 1990s. However, ORS was not widely available due to weak distribution systems that caused frequent shortages (18,36,37).
- The Child Survival Program was implemented from 1998 to 2002. It increased knowledge of ORS preparation, but did not increase ORS use or availability (18,36,37).
- Integrated Community Care Management (iCCM) started in 2003 in collaboration with the Ministry of Health, Public Hygiene and Prevention. This covered the whole country, but was also accompanied by increases in medicine prices in order to cover supply costs (38).

2006 to 2012

- Fatick Partnership from 2007 to 2009 continued the work of BASICS in Fatick Region (18,36,37).
- The Community Health Program was implemented from 2006 to 2011 by six NGOs (ChildFund, Africare, Plan, World Vision, Catholic Relief Services and Counterpart International) to strengthen access to primary health care services in previously underserved rural communities (18,36,37).
- **Key event**: In 2008, The Ministry of Health, Public Hygiene and Prevention began promoting the new WHO and UNICEF guidelines for treating diarrhea with low-osmolality ORS and zinc together (39). Following this policy change, an ORS-zinc pilot program was launched in Thiès, Fatick and Kolda, and the iCCM guidelines for diarrhea were revised (39). In addition, in 2010, UNICEF provided an annual stock of zinc at the regional level, and capacity building began to implement the new iCCM guidelines (39). There was a lot of reliance on external implementation of this program (18,36,37).
- Zinc was introduced in community "health huts" in 2009. There were more than 1600 of these in Senegal by 2010. However, they still charged full price for ORS and zinc and few workers knew how to look for signs of dehydration (18,36,37).

2012 to 2017

- **Key event**: In 2012, the Ministry of Health, Public Hygiene and Prevention partnered with the Canada-based Zinc Alliance for Child Health (ZACH) to launch a nation-wide ORS and zinc scale-up campaign, which was rolled out over 3 years (39,40). The project aimed to use behavior change communication to strengthen knowledge of diarrhea and improve awareness of the importance of treating childhood diarrhea with ORS and zinc (39,40). It aimed to train community actors, mothers and guardians, and health workers. In addition, it aimed to work at all levels of government to improve availability of supplies and treatment in ways that are affordable for communities, families, and the health system (39,40). This included making ORS and zinc available in all public and private communities health facilities, as well as strengthening organizational management systems (39). Finally, it aimed to establish systems for evaluating progress and ensuring sustainability of the program (39).
- In 2013, the government committed to pursuing universal health coverage, but this will not be fully implemented until 2022 (41).

6.0 Additional file 1: Figures

Additional file 1: Figure S1. Geospatial modelling flowchart 11
Additional file 1: Figure S2. In-sample and out-of-sample validation plots for any ORS. 12
Additional file 1: Figure S3. In-sample and out-of-sample validation plots for only RHF. 13
Additional file 1: Figure S4. In-sample and out-of-sample validation plots for no ORT. 14
Additional file 1: Figure S5. Oral rehydration therapy coverage by district/cercle/department and year. 15
Additional file 1: Figure S6. Numbers of untreated children by district/cercle/department and year. 18

Additional file 1: Figure S1. Geospatial modelling flowchart

The geospatial modelling process consists of four sections. First (in blue), we compile all available survey data that can be referenced to a coordinate/point (e.g., survey cluster) or small polygon unit and calculate any ORS, only RHF, and no ORT coverage at the respective level (sections 1.0 and 2.0). Data matched to polygons are resampled into pseudo-points using a k-means clustering algorithm (section 2.3). Covariates are subsequently merged to the points and pseudo-points via a spatial join (section 2.4). Second (green), we use the point data and their associated covariates and a stacked generalisation ensemble model (section 3.2). The child models—boosted regression trees, generalised additive models, and elastic net regression—are fit using a five-fold cross validation process (section 3.3). The cross-validated predictions from each model then serve as the covariate values for the main/parent model (Spatio-temporal GPR model) (section 3.3). The predictions from when the child models are fit on all the data (rather than $4/5^{ths}$ implied by the cross-validation) are then used to create posterior predictions of any ORS, only RHF, and no ORT coverage in a 5 × 5-km grid for the years 2000–2017 or 2001–2018 (section 3.3). Finally (orange), we aggregate our estimates to second administrative units (section 3.6).

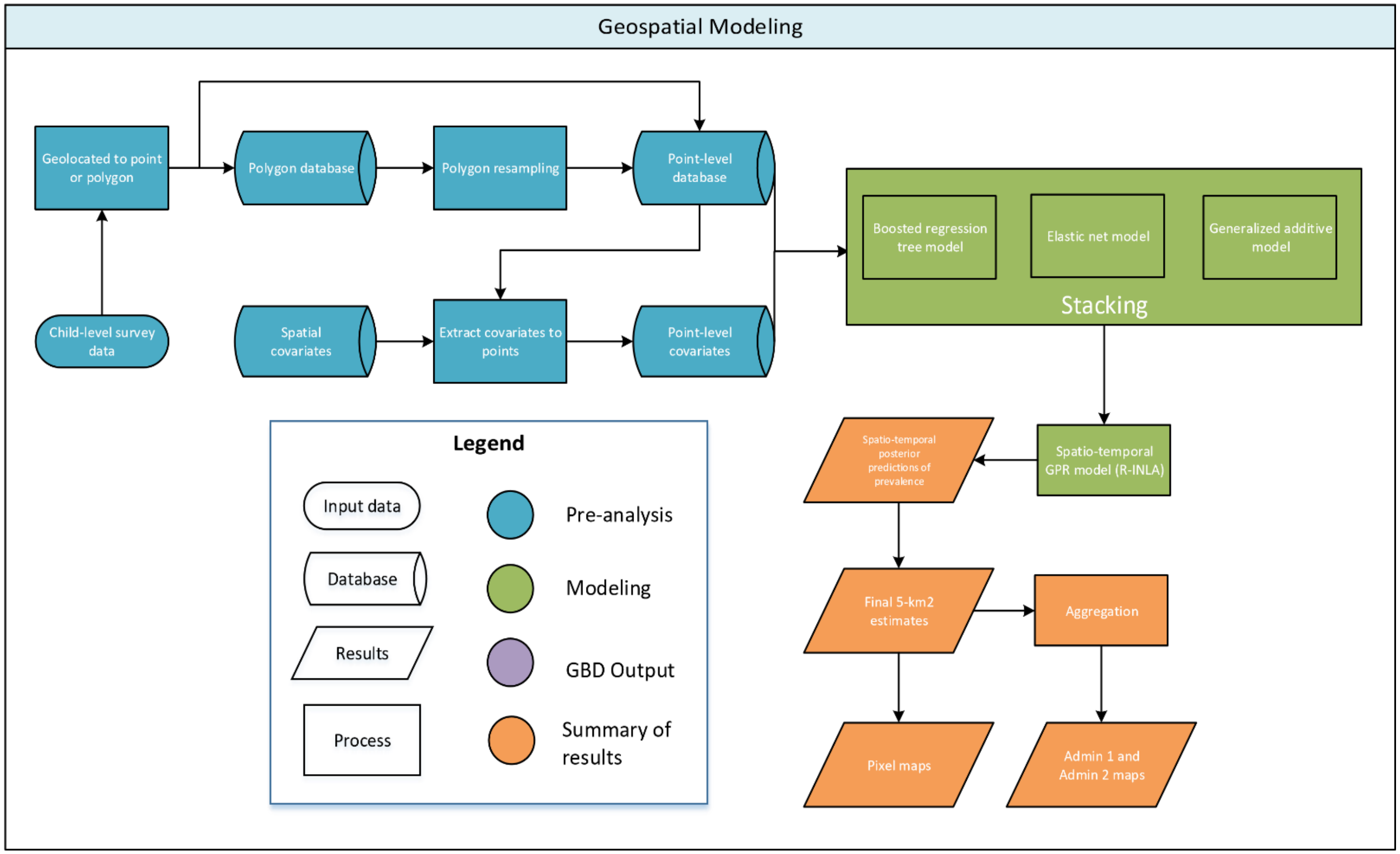

Additional file 1: Figure S2. In-sample and out-of-sample validation plots for any ORS.

Each plot shows the survey data on the x-axis and mean posterior predictions of coverage y-axis. The size of each dot is proportional to sample size in the underlying data. Estimates for in-sample **(a)** and out-of-sample **(b)** models are shown aggregated to second administrative levels for Mali (MLI), Senegal (SEN), and Sierra Leone (SLE). For corresponding in-sample and out-of-sample fit statistics see Additional file 1: Table S8.

a)

In-sample validation plot for any ORS by second administrative unit

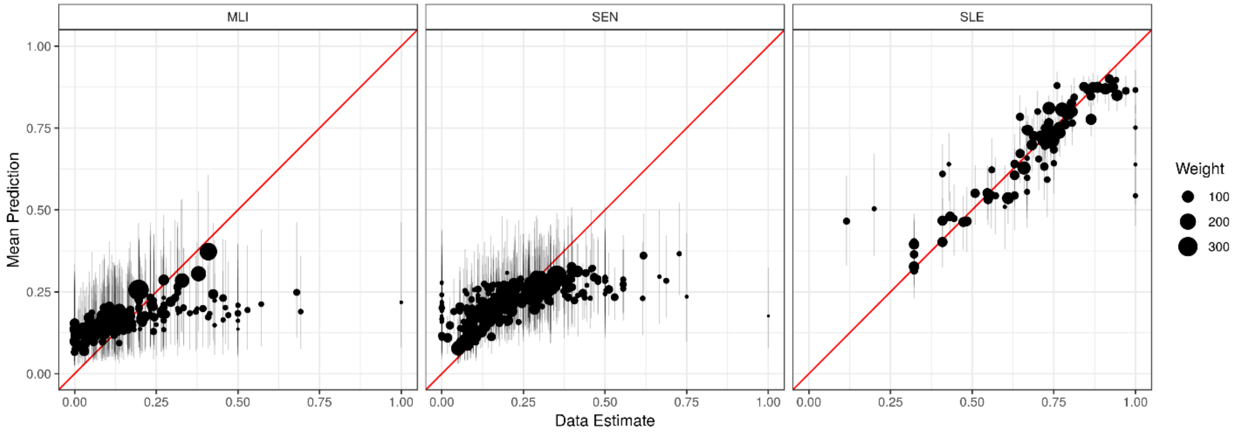

b)

Out-of-sample validation plot for any ORS by second administrative unit

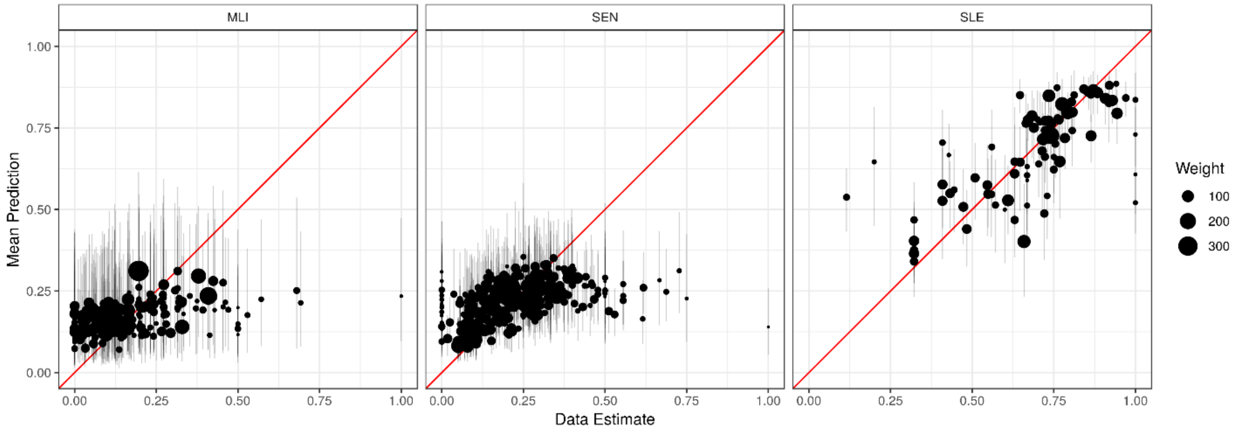

Additional file 1: Figure S3. In-sample and out-of-sample validation plots for only RHF.

Each plot shows the survey data on the x-axis and mean posterior predictions of coverage y-axis. The size of each dot is proportional to sample size in the underlying data. Estimates for in-sample **(a)** and out-of-sample **(b)** models are shown aggregated to second administrative levels for Mali (MLI), Senegal (SEN), and Sierra Leone (SLE). For corresponding in-sample and out-of-sample fit statistics see Additional file 1: Table S8.

a)

In-sample validation plot for only RHF by second administrative unit

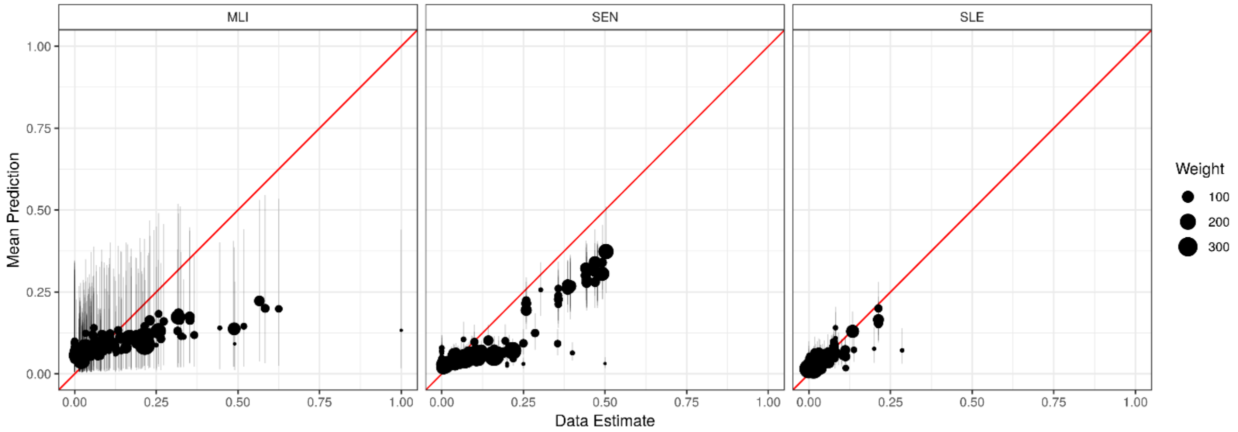

b)

Out-of-sample validation plot for only RHF by second administrative unit

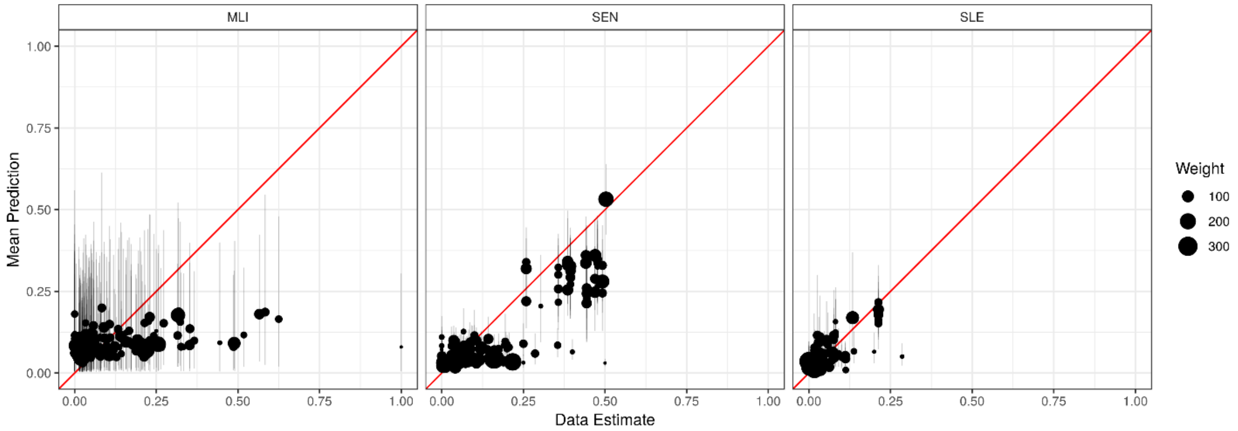

Additional file 1: Figure S4. In-sample and out-of-sample validation plots for no ORT.

Each plot shows the survey data on the x-axis and mean posterior predictions of coverage y-axis. The size of each dot is proportional to sample size in the underlying data. Estimates for in-sample **(a)** and out-of-sample **(b)** models are shown aggregated to second administrative levels for Mali (MLI), Senegal (SEN), and Sierra Leone (SLE). For corresponding in-sample and out-of-sample fit statistics see Additional file 1: Table S8.

a)

In-sample validation plot for no ORT by second administrative unit

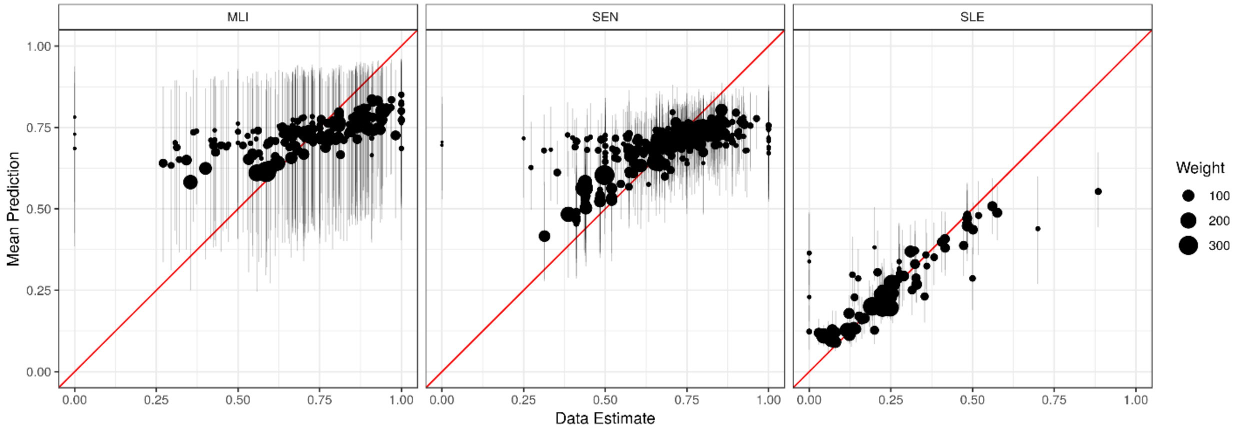

b)

Out-of-sample validation plot for no ORT by second administrative unit

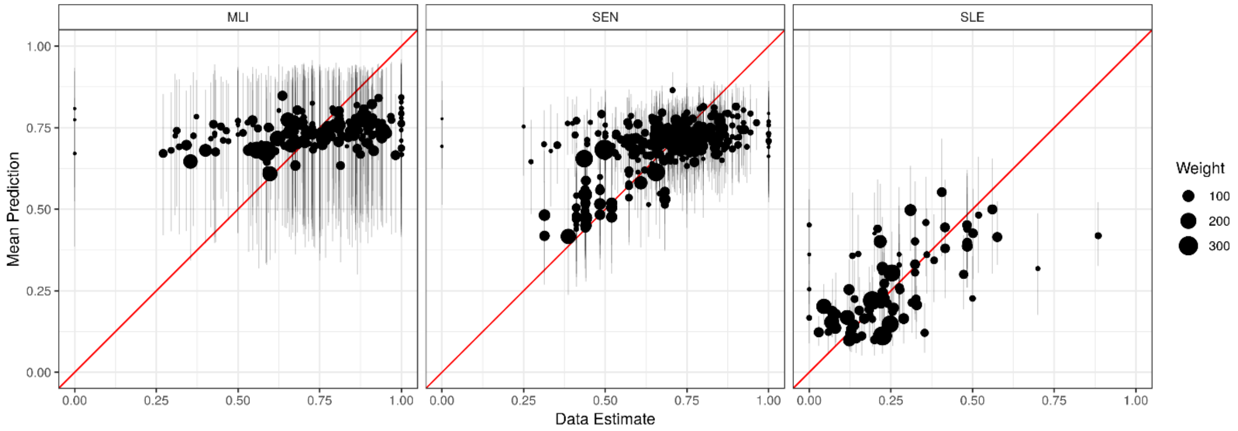

Additional file 1: Figure S5. Oral rehydration therapy coverage by district/cercle/department and year.
Mean oral rehydration therapy coverage estimates for a) Sierra Leone, b) Mali, and c) Senegal.

a) Sierra Leone
Coverage estimates by district for Any ORS, Only RHF, and No ORT in 2000, 2009, 2013, and 2017. Estimates correspond to results in Figure 2a,b and Additional file 1: Table S10a.

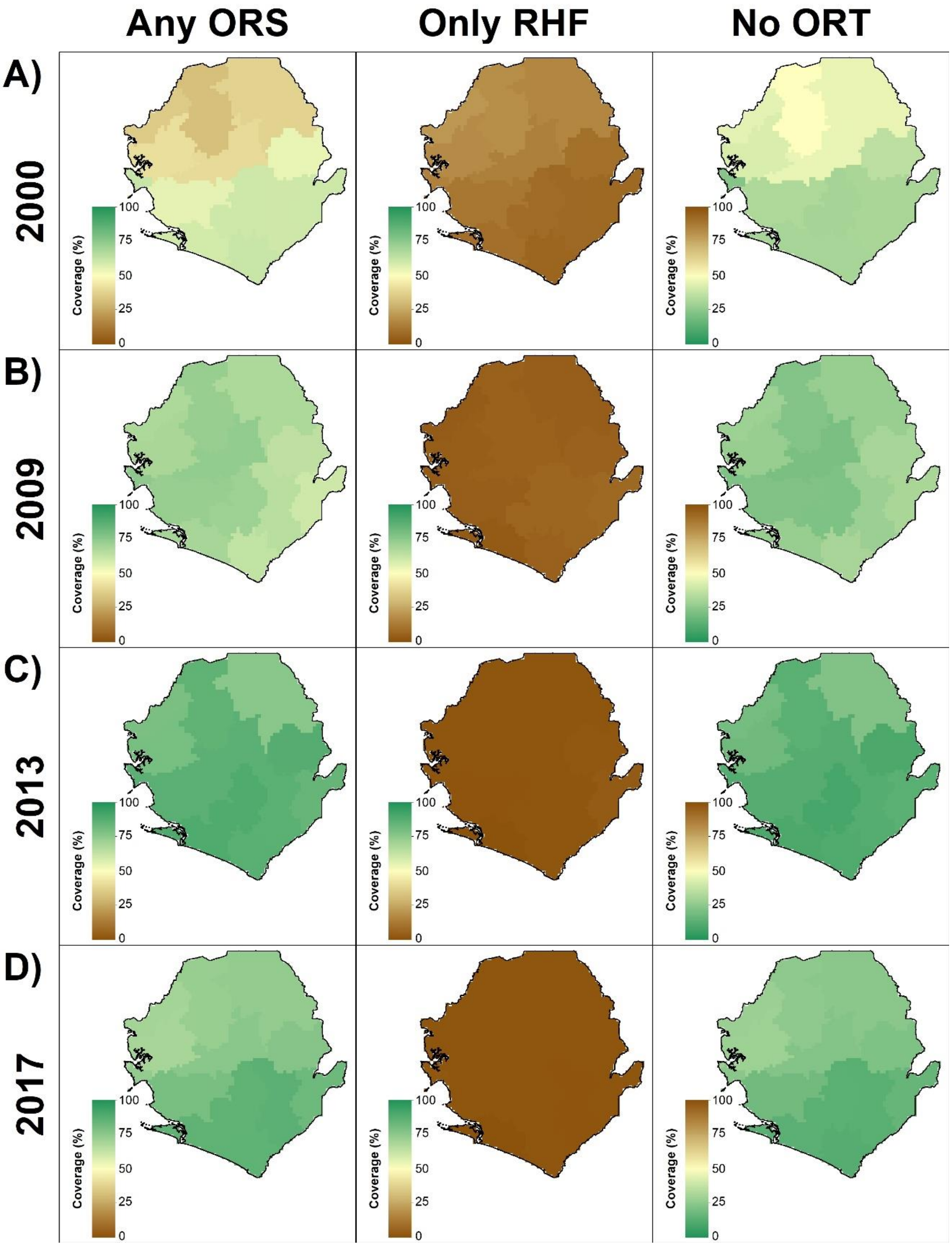

a) Mali

Coverage estimates by cercle for Any ORS, Only RHF, and No ORT in 2001, 2004, 2011, and 2018. Estimates correspond to results in Figure 3a,b and Additional file 1: Table S10b.

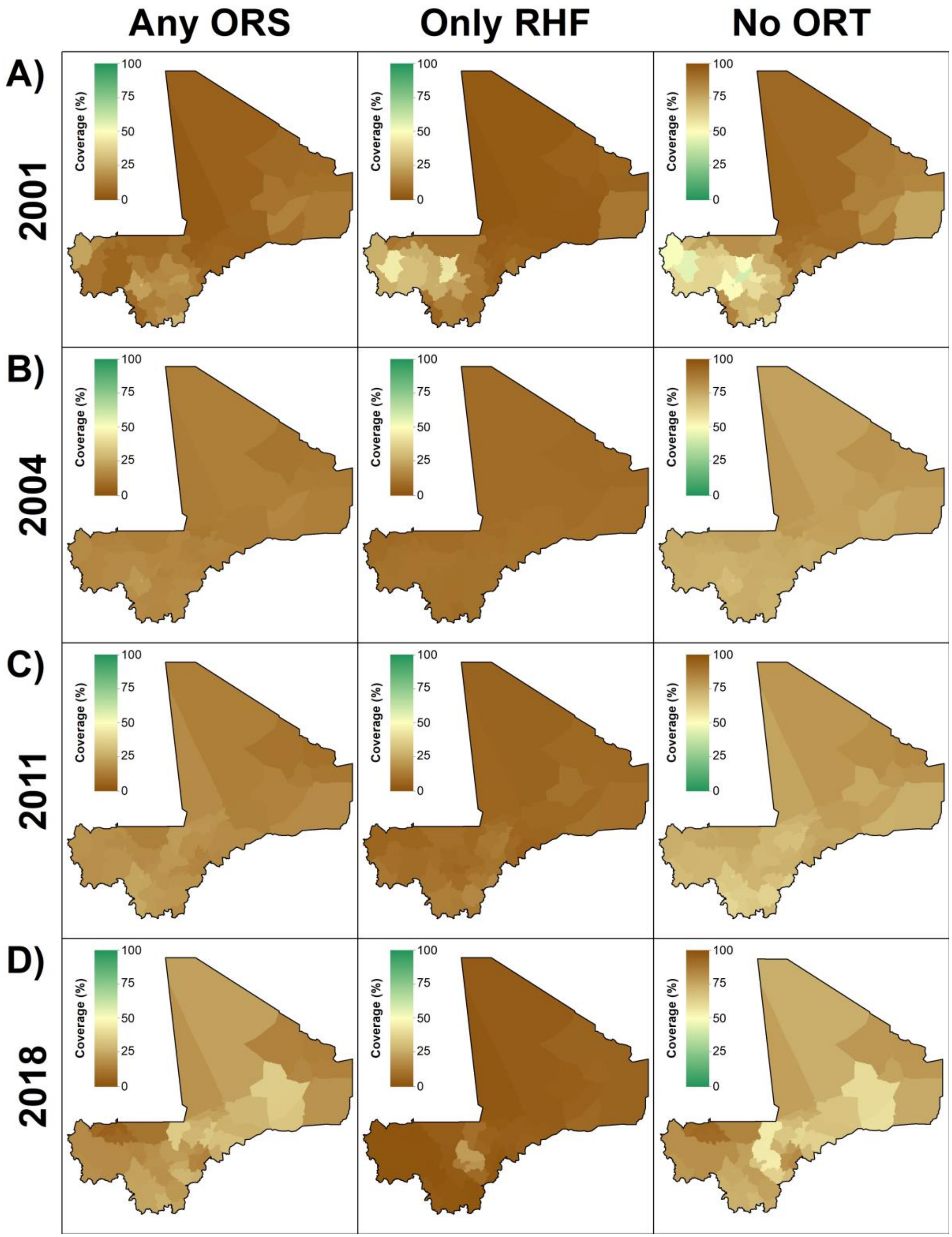

a) Senegal

Coverage estimates by department for Any ORS, Only RHF, and No ORT in 2000, 2006, 2012, and 2017. Estimates correspond to results in Figure 4a,b and Additional file 1: Table S10c.

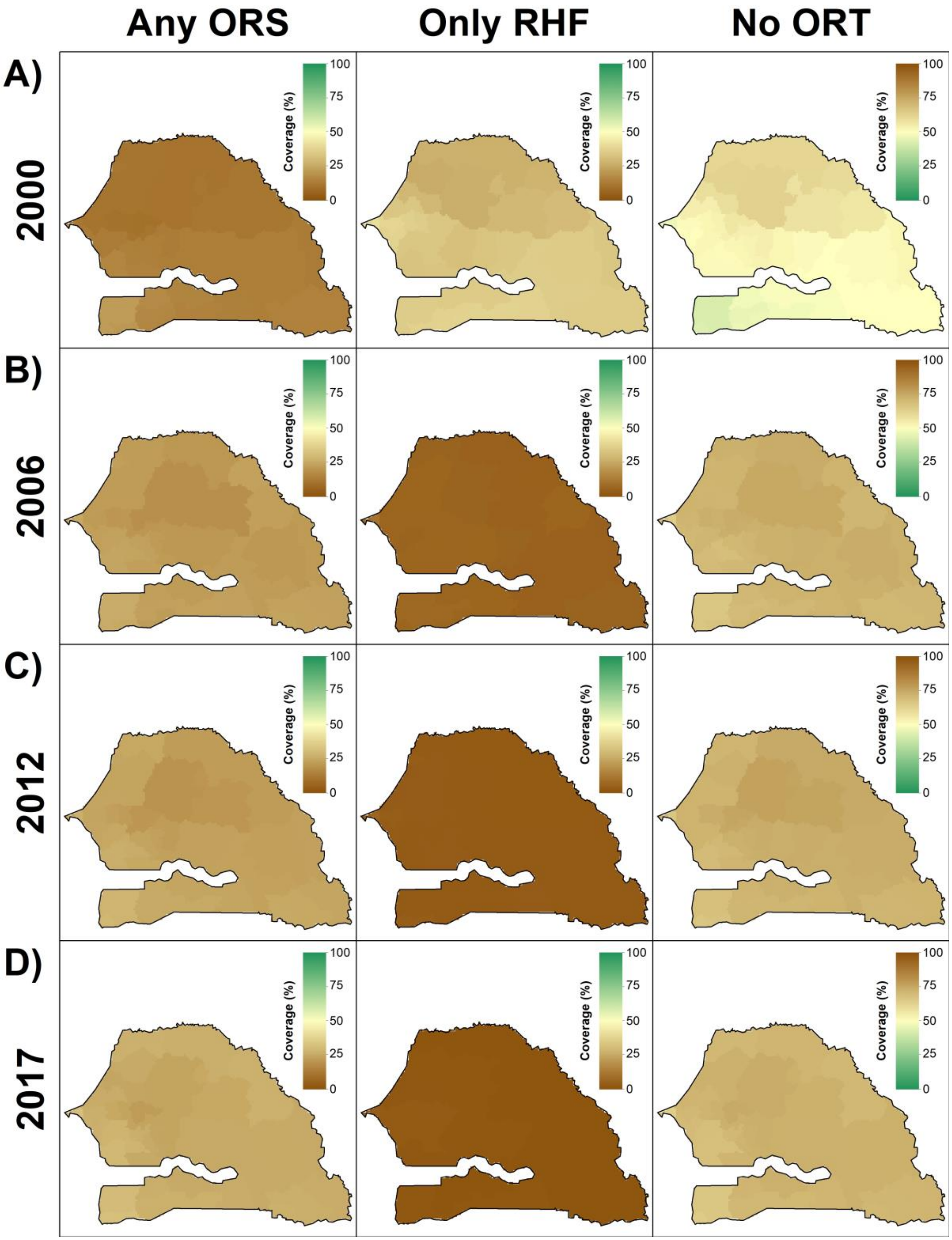

Additional file 1: Figure S6. Numbers of untreated children by district/cercle/department and year. Mean number of children with diarrhea untreated with ORT for a) Sierra Leone, b) Mali, and c) Senegal.

a) Sierra Leone

Number of children with diarrhea untreated by district for Any ORS, Only RHF, and Any ORT (either ORS or RHF) in 2000, 2009, 2013, and 2017. Mean numbers of children with diarrhea were calculated using previous estimates (42).

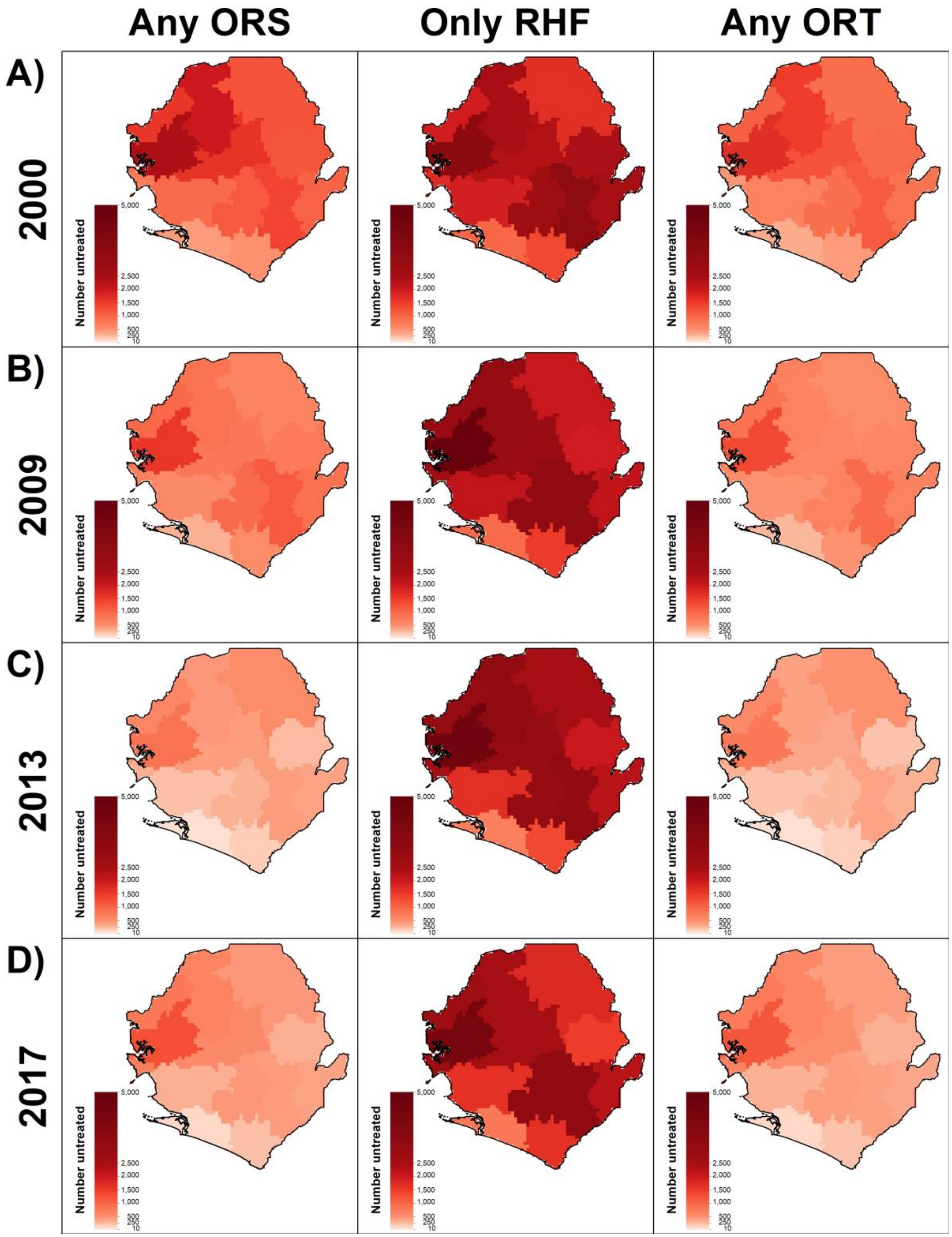

a) Mali

Number of children with diarrhea untreated by cercle for Any ORS, Only RHF, and Any ORT (either ORS or RHF) in 2001, 2004, 2011, and 2018. Mean numbers of children with diarrhea were calculated using previous estimates (42). For simplicity, 2018 numbers with diarrhea in Mali were assumed to be the same as 2017 numbers.

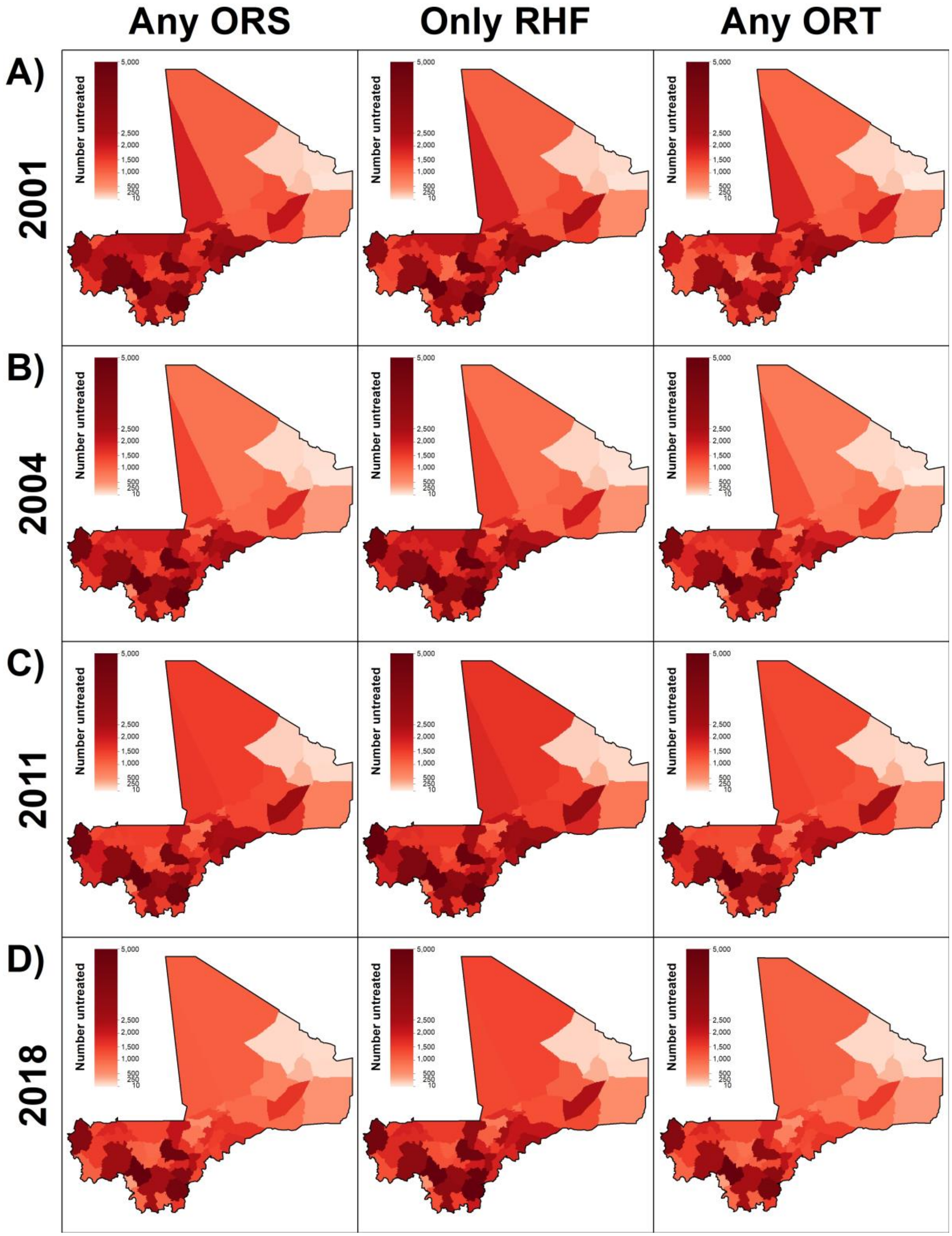

a) Senegal

Number of children with diarrhea untreated by department for Any ORS, Only RHF, and Any ORT (either ORS or RHF) in 2000, 2006, 2012, and 2017. Mean numbers of children with diarrhea were calculated using previous estimates (42).

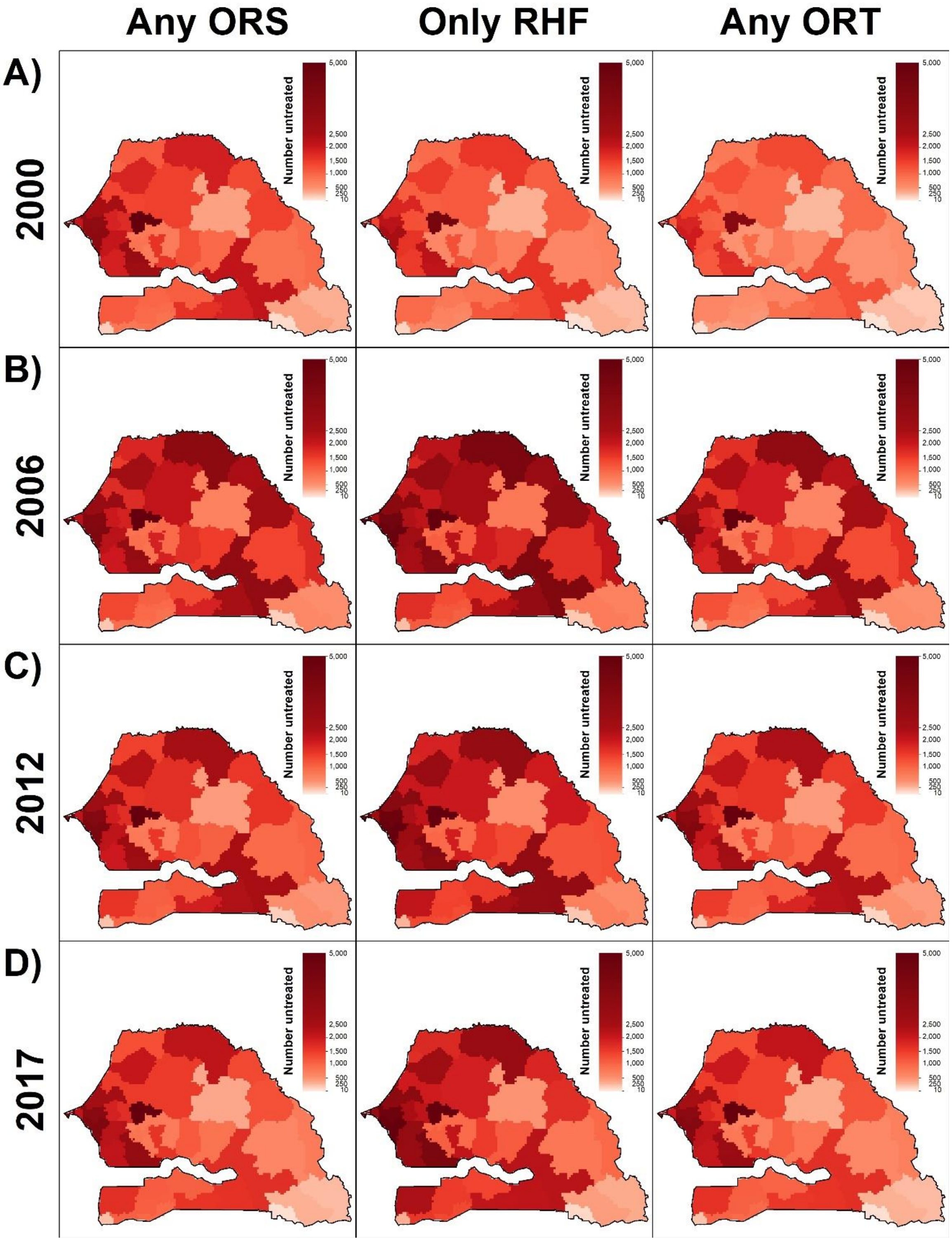

7.0 Additional file 1: Tables

Additional file 1: Table S1. Compliance for the Guidelines for Accurate and transparent Health Estimates Reporting 22
Additional file 1: Table S2. Input data sources....23
Additional file 1: Table S3. Only recommended home fluid (only RHF) definition adjustments 24
Additional file 1: Table S4. No oral rehydration therapy (No ORT) definition adjustments 24
Additional file 1: Table S5. Covariates used in mapping 25
Additional file 1: Table S6. Covariates used in ensemble covariate modelling via stacked generalisation, stratified by country....27
Additional file 1: Table S7. Fitted parameters....28
Additional file 1: Table S8. In-sample and out-of-sample fit statistics for any ORS, only RHF, and no ORT 31
Additional file 1: Table S9. Oral rehydration therapy coverage and numbers untreated by country and year. 32
Additional file 1: Table S10. Oral rehydration therapy coverage by district/cercle/department and year. 35
Additional file 1: Table S11. Changes in oral rehydration therapies in three time periods. 48

Additional file 1: Table S1. Compliance for the Guidelines for Accurate and transparent Health Estimates Reporting

Item #	Checklist item	Reported on page #
**Objectives and funding**		
**1**	Define the indicator(s), populations (including age, sex, and geographic entities), and time period(s) for which estimates were made.	Manuscript: Methods<br>Additional file 1: Sections 1.0, 2.0
**2**	List the funding sources for the work.	Manuscript: Methods
**Data Inputs**		
For all data inputs from multiple sources that are synthesised as part of the study:		
**3**	Describe how the data were identified and how the data were accessed.	Manuscript: Methods<br>Additional file 1: Section 2.0
**4**	Specify the inclusion and exclusion criteria. Identify all ad-hoc exclusions.	Manuscript: Methods<br>Additional file 1: Sections 1.0 and 2.0
**5**	Provide information on all included data sources and their main characteristics. For each data source used, report reference information or contact name/institution, population represented, data collection method, year(s) of data collection, sex and age range, diagnostic criteria or measurement method, and sample size, as relevant.	Manuscript: Methods<br>Additional file 1: Sections 1.0 and 2.0<br><br>Global Health Data Exchange (*link available upon publication*)
**6**	Identify and describe any categories of input data that have potentially important biases (e.g., based on characteristics listed in item 5).	Additional file 1: Section 2.0
For data inputs that contribute to the analysis but were not synthesised as part of the study:		
**7**	Describe and give sources for any other data inputs.	Manuscript: Methods<br>Additional file 1: Sections 2.0, 3.0
**8**	Provide all data inputs in a file format from which data can be efficiently extracted (e.g., a spreadsheet rather than a PDF), including all relevant meta-data listed in item 5. For any data inputs that cannot be shared because of ethical or legal reasons, such as third-party ownership, provide a contact name or the name of the institution that retains the right to the data.	Global Health Data Exchange (*link available upon publication*)
**9**	Provide a conceptual overview of the data analysis method. A diagram may be helpful.	Manuscript: Methods<br>Additional file 1: Section 3.0<br>Additional file 1: Figure S1
**10**	Provide a detailed description of all steps of the analysis, including mathematical formulae. This description should cover, as relevant, data cleaning, data pre-processing, data adjustments and weighting of data sources, and mathematical or statistical model(s).	Manuscript: Methods<br>Additional file 1: Sections 2.0, 3.0
**11**	Describe how candidate models were evaluated and how the final model(s) were selected.	Manuscript: Methods<br>Additional file 1: Sections 3.0, 4.0
**12**	Provide the results of an evaluation of model performance, if done, as well as the results of any relevant sensitivity analysis.	Manuscript: Methods<br>Additional file 1: Section 4.0<br>Additional file 1: Figures 2, 3, 4<br>Additional file 1: Table S8
**13**	Describe methods for calculating uncertainty of the estimates. State which sources of uncertainty were, and were not, accounted for in the uncertainty analysis.	Manuscript: Methods and Discussion<br>Additional file 1: Sections 3.0
**14**	State how analytic or statistical source code used to generate estimates can be accessed.	https://github.com/ihmeuw/lbd/tree/ors-rhf-2020 (*link live available upon publication*)
**Results and Discussion**		
**15**	Provide published estimates in a file format from which data can be efficiently extracted.	Additional file 1: Tables 9, 10, 11<br>Raster files for spatial data and CSVs of estimates at Global Health Data Exchange (*link available upon publication*)
**16**	Report a quantitative measure of the uncertainty of the estimates (e.g. uncertainty intervals).	Manuscript: Results and Figures<br>Additional file 1: Section 3.0<br>Additional file 1: Tables 9, 10, 11
**17**	Interpret results in light of existing evidence. If updating a previous set of estimates, describe the reasons for changes in estimates.	Manuscript: Discussion
**18**	Discuss limitations of the estimates. Include a discussion of any modelling assumptions or data limitations that affect interpretation of the estimates.	Manuscript: Discussion

Additional file 1: Table S2. Input data sources

Data sources included in the study, listed by country, global health data exchange (GHDx) unique identifier (NID), source, and year of survey (median interview year across primary sampling units).

Country	GHDx NID	Source	Year
Mali	20315	MACRO DHS	2001
	20274	MACRO DHS	2006
	270627	UNICEF MICS	2010
	77388	MACRO DHS	2012
	248224	UNICEF MICS	2015
	398033	MACRO DHS	2018
Senegal	27044	UNICEF MICS	2000
	26855	MACRO DHS	2005
	56063	MACRO DHS	2011
	111432	MACRO DHS	2013
	191270	MACRO DHS	2014
	218592	MACRO DHS	2015
	287639	UNICEF MICS	2015
	286772	MACRO DHS	2016
	353526	MACRO DHS	2017
Sierra Leone	11639	UNICEF MICS	2000
	11649	UNICEF MICS	2005
	21258	MACRO DHS	2008
	76700	UNICEF MICS	2010
	131467	MACRO DHS	2013
	218619	UNICEF MICS	2017

Additional file 1: Table S3. Only recommended home fluid (only RHF) definition adjustments

Estimates of only RHF coverage for each survey that was adjusted by methods described in Additional file 1: Section 1.2. Coverage before adjustment is shown as "unadjusted" and coverage after adjustment is shown as "adjusted".

NID	Country	Source	Start Year	Unadjusted	Adjusted
11639	SLE	UNICEF_MICS	2000	0.176805	0.150391
27044	SEN	UNICEF_MICS	2000	0.5134	0.42325
248224	MLI	UNICEF_MICS	2015	0.028571	0.028619
270627	MLI	UNICEF_MICS	2010	0.147106	0.142821
287639	SEN	UNICEF_MICS	2015	0.189884	0.176073

Additional file 1: Table S4. No oral rehydration therapy (No ORT) definition adjustments

Estimates of no ORT (treatment with neither ORS nor RHF) coverage for each survey that was adjusted by methods described in Additional file 1: Section 1.2. Coverage before adjustment is shown as "unadjusted" and coverage after adjustment is shown as "adjusted".

NID	Country	Source	Start Year	Unadjusted	Adjusted
11639	SLE	UNICEF_MICS	2000	0.321169	0.367994
27044	SEN	UNICEF_MICS	2000	0.399272	0.470408
248224	MLI	UNICEF_MICS	2015	0.751126	0.748186
270627	MLI	UNICEF_MICS	2010	0.744623	0.746195
287639	SEN	UNICEF_MICS	2015	0.511869	0.534582

Additional file 1: Table S5. Covariates used in mapping

A variety of socio-economic and environmental variables were used to predict any ORS, only RHF, and no ORT coverage. Where available, the finest spatio-temporal resolution of gridded data sets was used.

Covariate	Temporal Resolution	Source	Reference
**Access to cities**	Static	Oxford	Weiss, D., Nelson, A., Gibson, H., *et al.* A global map of travel time to cities to assess inequalities in accessibility in 2015. *Nature* **533**, 333–336 (2018).
**Ratio of children dependents (age 0 to 14) to working adults (age 15 to 64)**	Static	WorldPop (derived)	Lloyd, C.T., Sorichetta, A., & Tatem, A.J. High resolution global gridded data for use in population studies. *Sci Data* **4**, 170001 (2017). https://doi.org/10.1038/sdata.2017.1<br><br>WorldPop. Get data. Available at: http://www.worldpop.org.uk/data/get_data/. (Accessed: 25th July 2017)
**Distance from rivers or lakes**	Static	Natural Earth Data (derived)	Natural Earth. Rivers and lake centerlines dataset. Available at: http://www.naturalearthdata.com/downloads/10m-physical-vectors/10m-rivers-lake-centerlines/. (Accessed: 24th July 2017)
**Nighttime lights[TV]**	Annual	NOAA DMSP satellite program (derived)	Savory, D.J., Andrade-Pacheco, R., Gething, P.W., Midekisa, A., Bennett, A., & Sturrock, H.J.W. Intercalibration and Gaussian Process Modelling of Nighttime Lights Imagery for Measuring Urbanization Trends in Africa 2000–2013. *Remote Sens.* **9**, 713 (2017). Available at: http://www.mdpi.com/2072-4292/9/7/713
**Elevantion**	Static	NOAA GLOBE	Hastings, David A. and Paula K. Dunbar. Global Land One-kilometer Base Elevation (GLOBE) Digital Elevation Model, Documentation, Volume 1.0. Key to Geophysical Records Documentation (KGRD) 34. National Oceanic and Atmospheric Administration, National Geophysical Data Center, 325 Broadway, Boulder, Colorado 80303, U.S.A (1999).<br>GLOBE Task Team and others (Hastings, David A., Paula K. Dunbar, Gerald M. Elphingstone, Mark Bootz, Hiroshi Murakami, Hiroshi Maruyama, Hiroshi Masaharu, Peter Holland, John Payne, Nevin A. Bryant, Thomas L. Logan, J.-P. Muller, Gunter Schreier, and John S. MacDonald), eds., 1999. The Global Land One-kilometer Base Elevation (GLOBE) Digital Elevation Model, Version 1.0. National Oceanic and Atmospheric Administration, National Geophysical Data Center, 325 Broadway, Boulder, Colorado 80305-3328, U.S.A. Available at: https://www.ngdc.noaa.gov/mgg/topo/globe.html. (Accessed: 16th February 2017)
**Fertility**	Annual	WorldPop (derived)	Lloyd, C.T., Sorichetta, A., & Tatem, A.J. High resolution global gridded data for use in population studies. *Sci. Data* **4**, 170001 (2017). https://doi.org/10.1038/sdata2017.1<br><br>WorldPop. Get data. Available at: http://www.worldpop.org.uk/data/get_data/. (Accessed: 25th July 2017)
**Population[TV]**	Annual	WorldPop	Lloyd, C.T., Sorichetta, A., & Tatem, A.J. High resolution global gridded data for use in population studies. *Sci. Data* **4**, 170001 (2017). https://doi.org/10.1038/sdata2017.1<br><br>WorldPop. Get data. Available at: http://www.worldpop.org.uk/data/get_data/. (Accessed: 25th July 2017)
**Aridity[TV]**	Annual	WorldClim (derived)	Zomer, R.J., Trabucco, A., Bossio, D.A., & Verchot, L.V. Climate change mitigation: A spatial analysis of global land suitability for clean development mechanism afforestation and reforestation. *Agriculture, Ecosystems & Environment* **126**, 67–80 (2008). https:/doi.org/10.1016/j.agee.2008.01.014<br><br>Global Aridity Index (Global-Aridity) and Global Potential Evapo-Transpiration (Global-PET) Methodology and Geospatial Dataset Description (2009). Available at: http://www.cgiar-csi.org/data/global-aridity-and-pet-database
**Urban or rural[TV]**	Annual	European Commission/GHS	Pesaresi, M., *et al*. Operating procedure for the production of the Global Human Settlement Layer from Landsat data of the epochs 1975, 1990, 2000, and 2014. (Publications Office of the European Union, 2016). Available at:

Covariate	Temporal Resolution	Source	Reference
			http://ghsl.jrc.ec.europa.eu/data.php
**Urban proportion of the location^{TV}**	Annual	MODIS	Friedl, M. & Sulla-Menashe, D. MCD12Q1v006.MODIS/Terra+Aqua Land Cover Type Yearly L3 Global 500m SIN Grid https://doi.org/10.5067/MODIS/MCD12Q1.006 (NASA EOSDIS Land Processes DAAC, 2019)
**Irrigation**	Static	University of Frankfurt and FAO	Siebert, S., Doll, P., Hoogeveen, J., Faures, J.-M., Frenken, K., & Feick, S. Development and validation of the global map of irrigation areas. *Hydrology and Earth System Sciences* **9**, (5) 535–547 (2005).<br><br>Goethe-Universität. Generation of a digital global map of irrigation areas. Available at: https://www.unifrankfurt.de/45218039/Global_Irrigation_Map. (Accessed: 25th July 2017). Also from: http://www.fao.org/nr/water/aquastat/irrigationmap/index10.stm
**Number of people whose daily vitamin A needs could be met (nutrient yield)**	Static	Herrero et al. (modelled)	Herrero, M., *et al*. Farming and the geography of nutrient production for human use: a transdisciplinary analysis. *Lancet Planet. Health* **1**, e33–e42 (2017).
**Prevalence of under-5 stunting ^{TV}**	Annual	Internally modelled	Kinyoki, D.K., Osgood-Zimmerman, A.E., Pickering, B.V., et al. Mapping child growth failure across low- and middle-income countries. Nature 577, 231–234 (2020). http://doi.org/10.1038/s41586-019-1878-8
**Prevalence of under-5 wasting^{TV}**	Annual	Internally modelled	Kinyoki, D.K., Osgood-Zimmerman, A.E., Pickering, B.V., et al. Mapping child growth failure across low- and middle-income countries. Nature 577, 231–234 (2020). http://doi.org/10.1038/s41586-019-1878-8
**Maternal education**	Annual	Internally modelled	Graetz, N., Woyczynski, L., Wilson, K.F. *et al*. Mapping disparities in education across low- and middle-income countries. *Nature* **577**, 235–238 (2020). https://doi.org/10.1038/s41586-019-1872-1

Additional file 1: Table S6. Covariates used in ensemble covariate modelling via stacked generalisation, stratified by country

Covariates used in each modelling region for the any ORS, only RHF, and no ORT generalised additive model (GAM), penalised regression with the elastic net penalty, and boosted regression tree (BRT) models in each country Mali, Senegal, and Sierra Leone.

**Covariate**	**any ORS**			**only RHF**			**no ORT**		
	Mali	Senegal	Sierra Leone	Mali	Senegal	Sierra Leone	Mali	Senegal	Sierra Leone
**Antenatal care coverage**	✓	✓		✓	✓		✓	✓	
**Access to cities**	✓	✓	✓	✓	✓	✓	✓	✓	✓
**Aridity**		✓		✓	✓		✓	✓	
**Ratio of dependents**	✓			✓			✓		
**Distance to rivers or lakes**	✓	✓	✓	✓	✓	✓	✓	✓	✓
**Maternal education**	✓	✓	✓	✓	✓	✓	✓	✓	✓
**Elevation**	✓	✓	✓	✓	✓	✓	✓	✓	✓
**Fertility**		✓	✓		✓	✓		✓	✓
**Urban or rural**		✓	✓		✓	✓		✓	✓
**Nutrient yield**	✓	✓	✓	✓	✓	✓	✓	✓	✓
**Irrigation**	✓	✓	✓	✓	✓	✓	✓	✓	✓
**Under-5 stunting**	✓			✓			✓		
**Under-5 wasting**	✓	✓	✓	✓	✓	✓	✓	✓	✓
**Population**		✓			✓			✓	

Additional file 1: Table S7. Fitted parameters

Posterior lower, median, and upper quantiles (0.025%, 0.50%, 0.975%) for the main parameters by region. The first four rows provide information on the fixed effects: the intercept (int) and the the covariates (gam, gbm, and enet) corresponding to the predicted ensemble rasters. Fitted values for the spatio-temporal field hyperparameters and the precision parameters (inverse variance) for random effects are shown in the bottom four rows. Table **a)** presents fitted parameters for any ORS, table **b)** presents fitted parameters for only RHF, and table **c)** presents fitted parameters for no ORT.

a) any ORS

	Quantiles	int	gam	gbm	enet	Nominal Range	Nominal Variance	Ar1 ρ	precis
**Mali**	0.025	-0.2474	-0.1367	-0.0463	0.3555	0.9857	0.3695	-0.6220	1311.8408
	0.500	-0.0201	0.1535	0.1915	0.6532	2.6662	0.5614	0.3327	13415.7514
	0.975	0.2066	0.4518	0.4288	0.9470	4.8910	0.7519	0.7688	68424.7677
**Senegal**	0.025	-0.1934	-0.1482	0.0681	0.3098	0.7369	0.1704	-0.3712	4.0874
	0.500	-0.0853	0.1284	0.3112	0.5619	2.2736	0.2686	0.1675	6.5593
	0.975	0.0225	0.4004	0.5549	0.8140	8.5983	0.4101	0.6523	10.7206
**Sierra Leone**	0.025	-0.6037	0.0670	-0.4337	0.4371	1.9083	0.4314	0.2517	1283.2924
	0.500	-0.1194	0.4061	-0.1516	0.7455	3.6275	0.6563	0.6923	12920.7753
	0.975	0.3648	0.7471	0.1302	1.0516	7.1172	1.0085	0.8779	66482.4416

b) only RHF

	Quantiles	int	gam	gbm	enet	Nominal Range	Nominal Variance	Ar1 ρ	precis
**Mali**	0.025	-0.9996	-0.3120	-0.1151	0.5765	1.8202	0.8994	-0.5464	1232.2215
	0.500	-0.6751	-0.0066	0.1201	0.8853	2.4584	1.0824	0.1841	14377.7707
	0.975	-0.3519	0.3038	0.3554	1.1907	3.4458	1.3079	0.5779	70673.4292
**Senegal**	0.025	-0.6738	-0.2853	-0.1187	0.6202	0.4553	0.1695	-0.3510	1181.8001
	0.500	-0.4526	-0.0448	0.1396	0.9036	1.3965	0.2838	0.5125	12412.5203
	0.975	-0.2357	0.1985	0.3944	1.1901	4.6335	0.4795	0.9001	65495.7142
**Sierra Leone**	0.025	-0.6821	-0.8899	0.0041	0.9343	1.8194	0.4857	0.0029	1431.9786
	0.500	-0.0291	-0.4294	0.0158	1.4125	4.3478	0.7837	0.7161	14169.6830
	0.975	0.6216	0.0499	0.0299	1.8716	11.5097	1.2720	0.9308	70880.2595

c) no ORT

	Quantiles	int	gam	gbm	enet	Nominal Range	Nominal Variance	Ar1 ρ	precis
**Mali**	0.025	-0.0772	0.0476	-0.0932	0.3963	1.7220	0.6232	-0.4380	1161.2776
	0.500	0.1378	0.3204	0.0059	0.6639	2.4214	0.7556	0.1844	14359.1791
	0.975	0.3529	0.5926	0.1262	0.9303	3.2063	0.8891	0.5638	71009.8751
**Senegal**	0.025	-0.0238	-0.1587	0.1524	0.2488	0.5286	0.1327	-0.4712	4.8956
	0.500	0.0612	0.0885	0.4006	0.5118	1.6862	0.2190	0.1981	8.2698
	0.975	0.1462	0.3301	0.6488	0.7774	6.3994	0.3603	0.7214	14.0866
**Sierra Leone**	0.025	-0.2032	0.0334	0.0066	0.4215	1.1009	0.3947	0.3076	1288.8710
	0.500	0.0910	0.2903	0.0332	0.6764	2.0876	0.5336	0.6707	12570.0690
	0.975	0.3847	0.5495	0.0599	0.9286	5.7115	0.7197	0.8499	67079.4773

Additional file 1: Table S8. In-sample and out-of-sample fit statistics for any ORS, only RHF, and no ORT

Calculated mean error (Mean err.), root-mean-squared-error (RMSE), median sample side (Median SS), correlation between data and predictions (Corr.), and the proportion of observed out-of-sample data that fall within our predicted 95% uncertainty intervals (95% Cov.) aggregated to the second administrative unit. Fit statistics are shown for **a)** any ORS, **b)** only RHF, and **c)** no ORT and correspond to Additional file 1: Figures S2–S4.

a) any ORS

Country	Mean Err.	RMSE	Median SS	Corr.	95% Cov.
**In-sample**					
**Mali**	-0.019	58.544	33.000	0.767	0.952
**Senegal**	0.003	53.023	32.000	0.809	0.987
**Sierra Leone**	0.002	64.430	47.843	0.924	0.974
**Out-of-sample**					
**Mali**	-0.022	58.545	33.000	0.505	0.914
**Senegal**	0.003	53.023	32.000	0.631	0.980
**Sierra Leone**	0.004	64.428	47.843	0.787	0.887

b) only RHF

Country	Mean Err.	RMSE	Median SS	Corr.	95% Cov.
**In-sample**					
**Mali**	0.025201	58.42732	32.83201	0.83822	0.967538
**Senegal**	0.031833	53.71814	32	0.958387	0.767769
**Sierra Leone**	-0.00119	86.05985	54.40851	0.901452	0.989033
**Out-of-sample**					
**Mali**	0.026229	58.42661	32.83201	0.489817	0.951237
**Senegal**	0.025581	53.72277	32	0.902352	0.844649
**Sierra Leone**	-0.00587	86.06338	54.40851	0.804126	0.956522

c) no ORT

Country	Mean Err.	RMSE	Median SS	Corr.	95% Cov.
**In-sample**					
**Mali**	0.008091	58.43968	32.83201	0.77476	0.983894
**Senegal**	-0.00586	53.74613	32	0.837809	0.984748
**Sierra Leone**	-9.92E-05	86.05907	54.40851	0.92928	0.985337
**Out-of-sample**					
**Mali**	0.002997	58.44338	32.83201	0.440505	0.936616
**Senegal**	-0.00753	53.74739	32	0.67921	0.932377
**Sierra Leone**	-0.0053	86.06305	54.40851	0.684657	0.926448

Additional file 1: Table S9. Oral rehydration therapy coverage and numbers untreated by country and year. Oral rehydration therapy coverage estimates at the national level for a) Sierra Leone, b) Mali, and c) Senegal.

a) Sierra Leone

Coverage estimates at the national level for Any ORS, Only RHF, and No ORT from 2000 to 2017. Estimates are shown as percentages in the format: mean (2.5%–97.5% uncertainty interval). Estimates of the number (in thousands) of children with diarrhea untreated with any ORS or with any ORT (either ORS or RHF) are also shown. Mean numbers of children with diarrhea from 2000 to 2017 in each country were calculated using previous estimates (42).

	Coverage (percent)			Number untreated (thousands)	
**Year**	Any ORS	Only RHF	No ORT	Any ORS	Any ORT
**2000**	51.8 (48.1 – 55.5)	12.4 (10.1 – 14.8)	35.8 (31.9 – 39.5)	17.1 (15.8 – 18.4)	12.7 (11.3 – 14)
**2001**	53.4 (42.5 – 63.6)	11.1 (5.2 – 19.2)	35.6 (26.5 – 45)	17.3 (13.5 – 21.3)	13.2 (9.8 – 16.7)
**2002**	54.6 (41.9 – 66.3)	9.7 (3.8 – 19.3)	35.7 (25.3 – 47.5)	17.6 (13 – 22.5)	13.8 (9.8 – 18.4)
**2003**	55.8 (44.3 – 67.5)	8.2 (2.9 – 16.9)	36 (25.7 – 46.9)	17.8 (13.1 – 22.4)	14.5 (10.3 – 18.9)
**2004**	56 (45.9 – 65.7)	7.1 (3.1 – 13.3)	36.9 (27.4 – 47)	18.1 (14.1 – 22.3)	15.2 (11.3 – 19.3)
**2005**	54.7 (50.6 – 58.2)	6 (4.4 – 7.9)	39.3 (35.5 – 43.2)	18.8 (17.4 – 20.5)	16.3 (14.7 – 17.9)
**2006**	58.7 (49.8 – 67.4)	6.3 (2.7 – 11.8)	35 (25.8 – 43.8)	17 (13.4 – 20.7)	14.4 (10.6 – 18.1)
**2007**	61.6 (52 – 70)	6.4 (2.8 – 12.5)	32 (23.8 – 41.3)	15.5 (12.1 – 19.4)	12.9 (9.6 – 16.7)
**2008**	64.6 (59.9 – 69.2)	6.2 (4.1 – 8.8)	29.3 (25.1 – 33.6)	14 (12.2 – 15.9)	11.6 (9.9 – 13.3)
**2009**	69.6 (62.1 – 76.1)	4.9 (2.3 – 8.7)	25.5 (19.4 – 33.1)	11.8 (9.2 – 14.6)	9.9 (7.5 – 12.8)
**2010**	73.9 (71 – 76.5)	3.6 (2.6 – 5)	22.4 (20 – 25)	9.9 (8.9 – 11)	8.5 (7.6 – 9.5)
**2011**	77.2 (69.9 – 83.3)	2.9 (1.2 – 5.6)	19.9 (13.9 – 27.4)	8.5 (6.3 – 11.3)	7.5 (5.2 – 10.3)
**2012**	80.7 (73.9 – 85.9)	2.2 (0.9 – 4.3)	17.1 (11.9 – 24)	7.2 (5.2 – 9.7)	6.3 (4.4 – 8.9)
**2013**	85.1 (83.3 – 86.7)	1.5 (1 – 2.1)	13.5 (11.8 – 15.2)	5.5 (4.9 – 6.1)	4.9 (4.3 – 5.5)
**2014**	82.3 (76.3 – 87.6)	1.7 (0.7 – 3.6)	16 (10.8 – 22.2)	6.4 (4.5 – 8.6)	5.8 (3.9 – 8)
**2015**	79.9 (71.9 – 86.2)	1.8 (0.6 – 4.1)	18.3 (12.2 – 26.2)	7.2 (4.9 – 10)	6.5 (4.3 – 9.3)
**2016**	78.3 (71.1 – 84.1)	1.7 (0.6 – 3.4)	20 (14.2 – 27.6)	7.6 (5.6 – 10.1)	7 (5 – 9.7)
**2017**	77.4 (74.2 – 80.3)	1.5 (0.9 – 2.6)	21.1 (18.1 – 24.2)	7.8 (6.8 – 8.9)	7.3 (6.3 – 8.4)

b) Mali

Coverage estimates at the national level for Any ORS, Only RHF, and No ORT from 2001 to 2018. Estimates are shown as percentages in the format: mean (2.5%–97.5% uncertainty interval). Estimates of the number (in thousands) of children with diarrhea untreated with any ORS or with any ORT (either ORS or RHF) are also shown. Mean numbers of children with diarrhea from 2000 to 2017 in each country were calculated using previous estimates (42). For simplicity, 2018 numbers with diarrhea in Mali were assumed to be the same as 2017 numbers.

	**Coverage (percent)**			**Number untreated (thousands)**	
**Year**	Any ORS	Only RHF	No ORT	Any ORS	Any ORT
**2001**	14.4 (11.8 – 17)	16.3 (13.7 – 19)	69.3 (67 – 71.3)	104.2 (101.1 – 107.4)	84.3 (81.5 – 86.8)
**2002**	15.3 (8.1 – 25)	12.8 (5.9 – 21.8)	71.9 (59.5 – 82.9)	103.8 (91.9 – 112.7)	88.1 (72.9 – 101.7)
**2003**	15.9 (8 – 26)	11.1 (5.2 – 19.9)	73 (60.2 – 83.6)	103.7 (91.2 – 113.4)	90 (74.2 – 103)
**2004**	16.3 (8.4 – 26.5)	10.2 (4.4 – 19)	73.5 (60.9 – 84.5)	103.7 (91.2 – 113.6)	91.1 (75.5 – 104.7)
**2005**	16.5 (8.8 – 26.2)	9.8 (4.2 – 17.7)	73.7 (61.5 – 83.8)	104.5 (92.3 – 114)	92.2 (76.9 – 104.9)
**2006**	14.6 (12.1 – 17.4)	10.2 (7.9 – 12.7)	75.2 (72.7 – 77.4)	108.2 (104.6 – 111.3)	95.3 (92.1 – 98.1)
**2007**	16.6 (8.9 – 26.3)	10 (4.4 – 18.2)	73.4 (61.1 – 83.9)	107.1 (94.6 – 116.9)	94.2 (78.4 – 107.7)
**2008**	16.9 (8.7 – 27.3)	10.1 (4.3 – 18.6)	73 (59.6 – 83.9)	107.9 (94.4 – 118.5)	94.8 (77.4 – 108.9)
**2009**	16.3 (8.4 – 26.4)	11 (5.2 – 19.8)	72.7 (60.7 – 83.4)	109.7 (96.5 – 120.1)	95.3 (79.6 – 109.3)
**2010**	13.6 (11.2 – 16)	14.2 (11.8 – 16.6)	72.1 (70.2 – 73.8)	113.9 (110.8 – 117.1)	95.1 (92.6 – 97.4)
**2011**	19.3 (11.1 – 30.9)	10.5 (5.1 – 18.4)	70.2 (57.4 – 80.5)	105.5 (90.4 – 116.3)	91.8 (75.1 – 105.4)
**2012**	29.8 (25.8 – 33.6)	7.1 (4.9 – 10.2)	63.1 (59.7 – 66.6)	90.5 (85.7 – 95.7)	81.4 (77 – 86)
**2013**	23.3 (13.9 – 34.3)	6.8 (2.5 – 13.3)	69.9 (56.5 – 80.6)	97.7 (83.7 – 109.6)	89 (72 – 102.6)
**2014**	22.2 (13.3 – 32.5)	5.9 (2.4 – 12.1)	72 (59.8 – 82.3)	97.3 (84.4 – 108.4)	90 (74.7 – 102.9)
**2015**	23.1 (20.9 – 25.2)	3.7 (2.5 – 5.1)	73.2 (71.1 – 75.3)	93.8 (91.2 – 96.5)	89.3 (86.8 – 91.8)
**2016**	22 (12.7 – 33.7)	4.9 (1.8 – 10.1)	73.1 (60.5 – 83.8)	92.2 (78.4 – 103.2)	86.4 (71.5 – 99)
**2017**	22.6 (13.3 – 33)	4.6 (1.7 – 9.5)	72.8 (60.5 – 83.4)	88 (76.2 – 98.5)	82.8 (68.7 – 94.9)
**2018**	23 (20.5 – 25.6)	4.3 (2.9 – 5.9)	72.7 (70.3 – 75.2)	87.5 (84.6 – 90.4)	82.7 (79.9 – 85.5)

c) Senegal

Coverage estimates at the national level for Any ORS, Only RHF, and No ORT from 2000 to 2017. Estimates are shown as percentages in the format: mean (2.5%–97.5% uncertainty interval). Estimates of the number (in thousands) of children with diarrhea untreated with any ORS or with any ORT (either ORS or RHF) are also shown. Mean numbers of children with diarrhea from 2000 to 2017 in each country were calculated using previous estimates (42).

	**Coverage (percent)**			**Number untreated (thousands)**	
**Year**	Any ORS	Only RHF	No ORT	Any ORS	Any ORT
**2000**	13 (10 – 16.6)	34.8 (30.6 – 38.8)	52.3 (48 – 56.6)	74.1 (71 – 76.7)	44.5 (40.9 – 48.2)
**2001**	17 (13 – 21.8)	24.1 (19.4 – 29.2)	58.8 (53.7 – 63.7)	73.8 (69.6 – 77.4)	52.4 (47.8 – 56.7)
**2002**	19.7 (15.1 – 24.8)	20.1 (15.4 – 24.9)	60.3 (55.2 – 65.7)	77.1 (72.2 – 81.5)	57.9 (53 – 63.1)
**2003**	19.4 (15 – 24.3)	12.6 (9.2 – 16.8)	67.9 (62.2 – 73)	84.1 (79 – 88.7)	70.9 (64.9 – 76.2)
**2004**	20.7 (16.4 – 24.8)	10.6 (7.7 – 14)	68.7 (63.5 – 73.5)	88.9 (84.3 – 93.7)	77 (71.2 – 82.4)
**2005**	21.4 (18.1 – 24.9)	9 (6.8 – 11.5)	69.6 (65.8 – 73.1)	91.9 (87.8 – 95.8)	81.4 (77 – 85.5)
**2006**	22.4 (18.5 – 26.4)	6.6 (4.5 – 9)	71 (66.7 – 75.5)	91.5 (86.7 – 96.1)	83.7 (78.6 – 89)
**2007**	23.8 (20.1 – 27.8)	4.3 (3 – 6)	71.8 (67.3 – 75.8)	88.6 (84 – 92.9)	83.6 (78.2 – 88.2)
**2008**	25.4 (21.6 – 29.7)	3.6 (2.5 – 5)	70.9 (66.4 – 75.2)	84.5 (79.7 – 88.9)	80.4 (75.3 – 85.2)
**2009**	25.5 (21.5 – 29.6)	3.5 (2.4 – 5)	71 (66.4 – 75.4)	82.3 (77.7 – 86.6)	78.4 (73.3 – 83.2)
**2010**	25.3 (21.7 – 29.2)	3.5 (2.4 – 4.9)	71.2 (66.6 – 75.2)	81.4 (77.1 – 85.2)	77.5 (72.5 – 81.9)
**2011**	23.1 (20.3 – 26.1)	3.6 (2.7 – 4.7)	73.3 (69.8 – 76.5)	83.6 (80.2 – 86.5)	79.6 (75.8 – 83.1)
**2012**	24 (20.3 – 28.3)	3.7 (2.6 – 5.2)	72.2 (67.7 – 76.5)	82.1 (77.5 – 86.1)	78.1 (73.2 – 82.7)
**2013**	24.2 (21.3 – 27.5)	3.8 (2.9 – 4.9)	72 (68.3 – 75.3)	81.5 (77.9 – 84.7)	77.4 (73.4 – 81)
**2014**	23.7 (20.9 – 26.6)	3.7 (2.8 – 4.8)	72.6 (69.3 – 75.6)	81.6 (78.5 – 84.6)	77.6 (74.1 – 80.9)
**2015**	27.4 (24.5 – 30.3)	3.6 (2.8 – 4.5)	69 (65.8 – 72.2)	77.3 (74.3 – 80.5)	73.5 (70.1 – 76.9)
**2016**	25.8 (22.5 – 29.1)	3.2 (2.4 – 4.1)	71 (67.4 – 74.7)	78.9 (75.4 – 82.5)	75.6 (71.7 – 79.5)
**2017**	27.1 (23.4 – 30.7)	2.6 (1.9 – 3.6)	70.3 (66.5 – 74)	77.8 (73.9 – 81.6)	74.9 (70.9 – 78.9)

Additional file 1: Table S10. Oral rehydration therapy coverage by district/cercle/department and year. Oral rehydration therapy coverage estimates subnationally for a) Sierra Leone, b) Mali, and c) Senegal.

a) Sierra Leone

Coverage estimates by Province, District, Treatment (Any ORS, Only RHF, and No ORT) for 2000, 2009, 2013, and 2017. Estimates are shown as percentages in the format: mean (2.5%–97.5% uncertainty interval). Groups Western, Northern, and Southern correspond to regions shown in Figure 2.

Group	Province	District	Treatment	2000	2009	2013	2017
**Western**	Western	Western Rural	Any ORS	62.2 (54.6 – 69.2)	75.2 (62.6 – 84.5)	86.7 (82 – 90.4)	73.8 (64.9 – 81.3)
	Western	Western Rural	Only RHF	23.3 (16.8 – 30.8)	21.2 (12.1 – 33.9)	12.1 (8.5 – 16.7)	24.3 (17.2 – 33.1)
	Western	Western Rural	No ORT	14.5 (10.4 – 19.2)	3.6 (1.3 – 7.9)	1.2 (0.5 – 2.3)	2 (0.7 – 4.2)
	Western	Western Urban	Any ORS	61.1 (53.5 – 68.1)	72.5 (59 – 82.1)	84.8 (79.3 – 89.1)	69.2 (60.2 – 76.7)
	Western	Western Urban	Only RHF	24.3 (18.2 – 31.2)	23.7 (13.7 – 37.4)	13.9 (9.7 – 19.4)	28.7 (21.1 – 38.7)
	Western	Western Urban	No ORT	14.6 (10.2 – 19.8)	3.8 (1.3 – 8.4)	1.4 (0.6 – 2.6)	2.1 (0.7 – 4.5)
**Northern**	Northern	Bombali	Any ORS	32.7 (25.3 – 40.7)	72.9 (61.8 – 81.8)	85.7 (81.6 – 89.4)	72.9 (65.1 – 79.9)
	Northern	Bombali	Only RHF	49.6 (41.3 – 57.9)	22.4 (13.7 – 33.8)	12.9 (9.5 – 16.8)	25.6 (18.1 – 33.8)
	Northern	Bombali	No ORT	17.7 (12.5 – 23.8)	4.7 (1.8 – 9.7)	1.4 (0.7 – 2.4)	1.5 (0.6 – 3.2)
	Northern	Kambia	Any ORS	35.5 (27.3 – 44.3)	68 (55.8 – 78.6)	79.4 (75.7 – 83.1)	70.5 (61.1 – 78)
	Northern	Kambia	Only RHF	44.1 (33.9 – 54)	28.2 (17.8 – 40.7)	19.4 (15.8 – 23.1)	27.9 (20.1 – 37.2)
	Northern	Kambia	No ORT	20.3 (14.4 – 27.4)	3.7 (1.3 – 8.1)	1.2 (0.6 – 2.3)	1.6 (0.6 – 3.7)
	Northern	Koinadugu	Any ORS	38.2 (29 – 48.3)	68.2 (56.8 – 78.6)	76.3 (71 – 81.3)	74.2 (67 – 80)
	Northern	Koinadugu	Only RHF	46 (36.1 – 56.6)	27.3 (17.6 – 38.7)	22.2 (17.3 – 27.6)	24.4 (18.5 – 31.5)
	Northern	Koinadugu	No ORT	15.8 (10.2 – 23.1)	4.5 (1.6 – 9.7)	1.4 (0.8 – 2.5)	1.4 (0.6 – 3.1)
	Northern	Port Loko	Any ORS	41.1 (34.8 – 47.6)	68.4 (56 – 78.9)	80.4 (76.6 – 83.8)	69.8 (62.7 – 76.2)
	Northern	Port Loko	Only RHF	41.9 (34.9 – 49)	27.5 (17.9 – 40.1)	18.2 (15 – 21.9)	28.3 (22 – 35.2)
	Northern	Port Loko	No ORT	17 (12.9 – 21.4)	4.1 (1.6 – 8.5)	1.3 (0.8 – 2.3)	1.9 (0.8 – 3.8)
	Northern	Tonkolili	Any ORS	40.2 (32.7 – 47.4)	74.4 (64.5 – 82.8)	86.5 (83.2 – 89.3)	76.3 (70.4 – 81.7)
	Northern	Tonkolili	Only RHF	45.7 (37.6 – 54.2)	20.7 (12.6 – 30.6)	12.1 (9.4 – 15.5)	22.3 (16.8 – 28.5)
	Northern	Tonkolili	No ORT	14.1 (9.7 – 18.9)	4.9 (1.9 – 9.4)	1.4 (0.8 – 2.2)	1.3 (0.6 – 2.6)

**Southern and Eastern**	Eastern	Kailahun	Any ORS	61.1 (51.1 – 69.7)	61 (46.7 – 73.5)	83.8 (77.4 – 88.8)	82.6 (75.1 – 88.3)
	Eastern	Kailahun	Only RHF	31.6 (22.7 – 41.9)	31.8 (19.5 – 45.8)	13 (8.5 – 19.3)	15.7 (10.2 – 23.5)
	Eastern	Kailahun	No ORT	7.2 (4 – 11.5)	7.2 (2.7 – 15.1)	3.2 (1.5 – 6.1)	1.6 (0.6 – 3.6)
	Eastern	Kenema	Any ORS	60.4 (52.5 – 68.2)	65.7 (52.6 – 76.2)	86.7 (82 – 90.8)	86.5 (81.5 – 90.4)
	Eastern	Kenema	Only RHF	32 (23.9 – 40.4)	28 (17.4 – 41.2)	11.8 (7.9 – 16.6)	12.6 (8.9 – 17.6)
	Eastern	Kenema	No ORT	7.6 (4.7 – 11.4)	6.3 (2.5 – 12.5)	1.4 (0.7 – 2.6)	0.9 (0.4 – 1.8)
	Eastern	Kono	Any ORS	54.9 (46.2 – 63.5)	64 (49.6 – 75.9)	88 (84.5 – 90.9)	77.6 (69.7 – 84)
	Eastern	Kono	Only RHF	35.6 (26.8 – 45.2)	30.6 (19.1 – 44.7)	10 (7.1 – 13.6)	21.2 (14.9 – 29.2)
	Eastern	Kono	No ORT	9.4 (5.8 – 14.2)	5.3 (2 – 11.1)	2 (1.1 – 3.4)	1.3 (0.5 – 2.8)
	Southern	Bo	Any ORS	60.9 (50.9 – 70.1)	72.2 (60.2 – 81.9)	89.6 (85.9 – 92.6)	85.8 (80.6 – 90.3)
	Southern	Bo	Only RHF	30.3 (20.7 – 40.8)	21.1 (12.5 – 32.7)	8.8 (6 – 12.4)	13 (8.7 – 18.1)
	Southern	Bo	No ORT	8.8 (5.3 – 13.4)	6.7 (2.7 – 13.4)	1.6 (0.8 – 2.7)	1.2 (0.6 – 2.5)
	Southern	Bonthe	Any ORS	60.1 (47.7 – 72)	69.3 (55.4 – 80.8)	89.5 (84 – 93.4)	85.7 (78 – 91.6)
	Southern	Bonthe	Only RHF	30.9 (18.7 – 44.2)	27.6 (16 – 42.1)	9.9 (6 – 15.4)	13.6 (7.7 – 21.1)
	Southern	Bonthe	No ORT	9 (4.2 – 15.5)	3.1 (1.1 – 7.1)	0.6 (0.2 – 1.4)	0.7 (0.2 – 1.8)
	Southern	Moyamba	Any ORS	55.5 (45.8 – 64.4)	73.4 (62.5 – 82.8)	86.4 (82.6 – 89.9)	79.8 (72.1 – 85.9)
	Southern	Moyamba	Only RHF	32.4 (23.4 – 43)	22.1 (12.8 – 33.1)	12.4 (9 – 16.2)	18.6 (12.5 – 26.4)
	Southern	Moyamba	No ORT	12 (7.7 – 17.3)	4.5 (1.8 – 9.2)	1.1 (0.6 – 2)	1.5 (0.6 – 3.2)
	Southern	Pujehun	Any ORS	62.4 (50 – 74.3)	63.5 (49.2 – 77)	87.5 (82 – 91.9)	86.2 (80.4 – 91)
	Southern	Pujehun	Only RHF	30.7 (19 – 44.6)	31.5 (17.9 – 46.1)	11.1 (6.8 – 16.6)	12.3 (7.8 – 18)
	Southern	Pujehun	No ORT	6.9 (3.1 – 11.8)	5.1 (1.8 – 11.2)	1.4 (0.6 – 2.9)	1.5 (0.5 – 3.2)

b) Mali

Coverage estimates by Region, Cercle, Treatment (Any ORS, Only RHF, and No ORT) for 2001, 2004, 2011, and 2018. Estimates are shown as percentages in the format: mean (2.5%–97.5% uncertainty interval). Groups Bamako, Northern, and Southern correspond to regions shown in Figure 3.

Group	Region	Cercle	Treatment	2001	2004	2011	2018
Bamako	Bamako	Bamako	Any ORS	32.2 (25.6 – 38.7)	25.3 (5.5 – 54.6)	27.8 (8.2 – 56)	25.6 (20 – 31.8)
	Bamako	Bamako	Only RHF	38.3 (32 – 44.8)	65.2 (30.5 – 91)	61.9 (30 – 86.3)	73.6 (67.4 – 79.5)
	Bamako	Bamako	No ORT	29.4 (23.2 – 35.8)	9.4 (0.9 – 30.2)	10.3 (0.9 – 29.7)	0.8 (0.2 – 2.2)
Northern	Gao	Ansongo	Any ORS	9.2 (4.4 – 15.8)	15 (2.9 – 38.5)	17 (3.8 – 38.1)	33.5 (19.7 – 50)
	Gao	Ansongo	Only RHF	87.7 (80.5 – 93.1)	75.4 (48.5 – 93.2)	72.9 (45.6 – 91.6)	59.4 (42 – 74.8)
	Gao	Ansongo	No ORT	3.1 (0.9 – 7.4)	9.5 (1.3 – 26.7)	10.1 (1.7 – 27.8)	7.2 (1.8 – 17.2)
	Gao	Bourem	Any ORS	10.3 (5 – 18.2)	13.4 (2.8 – 31.9)	14.6 (4 – 33)	35.7 (25 – 48)
	Gao	Bourem	Only RHF	85.9 (76 – 92.2)	78.4 (53.4 – 93.3)	75.5 (52.5 – 91.9)	60.2 (47.5 – 71.6)
	Gao	Bourem	No ORT	3.8 (1 – 8.6)	8.3 (1.4 – 22.2)	9.9 (1.9 – 24.9)	4.1 (1.3 – 9.6)
	Gao	Gao	Any ORS	14.5 (7.2 – 24.8)	16.2 (2.9 – 40.6)	14.9 (3.1 – 35.8)	35.1 (24.3 – 47.1)
	Gao	Gao	Only RHF	82.1 (71 – 90.2)	75 (44.8 – 93.5)	77.7 (51.9 – 93.7)	59.9 (46.7 – 71.1)
	Gao	Gao	No ORT	3.4 (0.8 – 8.6)	8.9 (1 – 29.3)	7.3 (0.9 – 22.1)	5.1 (1.5 – 11.7)
	Gao	Ménaka	Any ORS	12.1 (5.2 – 22.5)	13.4 (4.1 – 28.6)	17.4 (5.9 – 34.4)	19.5 (8 – 38.4)
	Gao	Ménaka	Only RHF	76.5 (62.1 – 87.4)	77.5 (57.2 – 90.9)	73.5 (51.5 – 89.5)	75 (53.4 – 88.4)
	Gao	Ménaka	No ORT	11.4 (4.3 – 21.5)	9.1 (1.9 – 22.6)	9.1 (1.7 – 22.9)	5.5 (1 – 15.3)
	Kidal	Abeïbara	Any ORS	8.6 (2.2 – 20.2)	11.3 (2.3 – 28.4)	9.5 (2.1 – 24.9)	13.8 (4 – 31)
	Kidal	Abeïbara	Only RHF	85.5 (70 – 94.8)	80.1 (56.5 – 94)	83.4 (62.9 – 94.7)	80.4 (60 – 92.8)
	Kidal	Abeïbara	No ORT	5.9 (1.1 – 15.7)	8.6 (1.1 – 24.7)	7.2 (1.2 – 20.6)	5.7 (0.8 – 15.4)
	Kidal	Kidal	Any ORS	9.5 (3.9 – 18.1)	12.9 (2.8 – 31.4)	13 (2.7 – 33.1)	16.8 (8.5 – 27.4)
	Kidal	Kidal	Only RHF	86.2 (75.6 – 93.2)	79 (53.8 – 93.7)	78.9 (54.1 – 93.9)	76.7 (63.7 – 87.2)
	Kidal	Kidal	No ORT	4.3 (1.1 – 9.9)	8 (1 – 23.7)	8.1 (1.1 – 22.5)	6.6 (1.9 – 13.9)
	Kidal	Tessalit	Any ORS	8.1 (2.7 – 16.7)	11.1 (2.8 – 27)	10.6 (2.6 – 25.3)	15.2 (5.1 – 31.6)
	Kidal	Tessalit	Only RHF	86.9 (74.8 – 94)	80.8 (59.4 – 93.6)	82.3 (63.1 – 94)	79.3 (59.8 – 92.2)
	Kidal	Tessalit	No ORT	5 (1.1 – 12.2)	8.1 (1.4 – 22)	7.1 (1.4 – 18.4)	5.6 (1 – 16.4)

	Kidal	Tin-Essako	Any ORS	9.6 (2.8 – 21.2)	12.3 (2.6 – 30.6)	11.8 (2.4 – 30.1)	14.8 (4.7 – 32)
	Kidal	Tin-Essako	Only RHF	84.7 (69.7 – 94.1)	79.5 (55.7 – 93.8)	80.7 (58.4 – 94)	79.4 (59.7 – 92.5)
	Kidal	Tin-Essako	No ORT	5.8 (1 – 15)	8.1 (1.2 – 24.5)	7.4 (1 – 21.5)	5.8 (0.9 – 15.7)
	Mopti	Bandiagara	Any ORS	6.7 (3.1 – 11.4)	15.6 (3.2 – 39.1)	17.5 (4.6 – 38.2)	32.5 (22.3 – 43)
	Mopti	Bandiagara	Only RHF	87.4 (80.2 – 92.7)	74.5 (46 – 92.7)	74.6 (47.1 – 91.8)	63 (51.9 – 73.6)
	Mopti	Bandiagara	No ORT	6 (2.5 – 11)	9.9 (1.1 – 28.3)	7.9 (1.1 – 23.7)	4.5 (1.5 – 9.5)
	Mopti	Bankass	Any ORS	6.4 (2.9 – 11.5)	15.1 (2.9 – 37.7)	16.7 (3.8 – 40.9)	25.9 (17.2 – 37.1)
	Mopti	Bankass	Only RHF	82 (73 – 89.3)	74.5 (45.4 – 92.8)	75.7 (44.9 – 92.7)	70.4 (59.2 – 80.2)
	Mopti	Bankass	No ORT	11.6 (6.3 – 18.8)	10.4 (1.3 – 30.8)	7.6 (0.8 – 23.5)	3.6 (1.2 – 8.1)
	Mopti	Djenné	Any ORS	5.7 (2.3 – 11)	14.3 (2.7 – 37.3)	15.1 (3 – 36.5)	18.5 (10.1 – 29.8)
	Mopti	Djenné	Only RHF	89.4 (81.6 – 94.3)	75.3 (46 – 92.7)	72.2 (42.7 – 91.3)	78.6 (66 – 87.6)
	Mopti	Djenné	No ORT	4.9 (1.9 – 9.5)	10.4 (1.2 – 30.8)	12.7 (2 – 33.9)	3 (0.7 – 8.1)
	Mopti	Douentza	Any ORS	4.8 (2.1 – 8.6)	13.2 (2.9 – 31.8)	17.4 (5.8 – 34.6)	27.4 (14.2 – 43.9)
	Mopti	Douentza	Only RHF	91.2 (85.7 – 95.4)	77.3 (53.2 – 92.7)	76 (52.6 – 91)	68.4 (49.7 – 83)
	Mopti	Douentza	No ORT	4.1 (1.6 – 7.9)	9.5 (1.6 – 25.7)	6.6 (1.1 – 18)	4.2 (0.9 – 11.6)
	Mopti	Koro	Any ORS	6.4 (2.7 – 11.7)	14.9 (3.2 – 38.4)	17 (4.3 – 38.7)	32.2 (20.6 – 45.3)
	Mopti	Koro	Only RHF	86.7 (78.7 – 92.2)	75.4 (48 – 93.2)	77.7 (52.5 – 93.1)	63.1 (48.8 – 75.5)
	Mopti	Koro	No ORT	7 (3.2 – 12.9)	9.7 (1.3 – 27.1)	5.3 (0.7 – 17.2)	4.7 (1.4 – 10.8)
	Mopti	Mopti	Any ORS	5.1 (2.6 – 8.8)	15.3 (3.4 – 37.1)	18.9 (5.4 – 44.3)	34.4 (23.1 – 46.4)
	Mopti	Mopti	Only RHF	89.8 (84.6 – 93.7)	75.4 (47.9 – 92.4)	68.2 (41 – 88.1)	61.1 (49.2 – 73.1)
	Mopti	Mopti	No ORT	5.1 (2.5 – 8.8)	9.3 (1.1 – 27.5)	13 (2.4 – 33.6)	4.5 (1.4 – 10.4)
	Mopti	Ténenkou	Any ORS	4.4 (2.1 – 7.8)	14.2 (2.5 – 35)	19 (5.4 – 42)	29.1 (15.7 – 46.4)
	Mopti	Ténenkou	Only RHF	91.1 (86.1 – 94.5)	76.1 (48.1 – 92.7)	69 (41.5 – 89.5)	66 (47 – 81.2)
	Mopti	Ténenkou	No ORT	4.5 (2.3 – 8)	9.7 (1.2 – 30.2)	12.1 (1.8 – 30.9)	4.9 (1 – 13)
	Mopti	Youwarou	Any ORS	3.4 (1.4 – 6.6)	12.1 (2.3 – 32.1)	20 (5.4 – 45.7)	27.9 (16.3 – 40.9)
	Mopti	Youwarou	Only RHF	92.8 (88.3 – 95.9)	79.5 (53.8 – 94.7)	70 (38.4 – 90.1)	69.3 (55.2 – 81.4)
	Mopti	Youwarou	No ORT	3.8 (1.7 – 6.7)	8.4 (0.9 – 27.4)	10 (1.4 – 28.4)	2.8 (0.7 – 8)
	Timbuktu	Diré	Any ORS	3.4 (1.1 – 7.6)	13.5 (2.2 – 37.3)	19.2 (4.8 – 44)	21.6 (14.1 – 30.5)
	Timbuktu	Diré	Only RHF	93.7 (87.6 – 97.2)	77.6 (45.1 – 95.2)	74.3 (44.6 – 93.2)	76 (66.7 – 84.1)

	Timbuktu	Diré	No ORT	2.8 (0.7 – 6.8)	8.9 (0.8 – 27.1)	6.5 (0.6 – 22.8)	2.4 (0.7 – 5.8)
	Timbuktu	Goundam	Any ORS	3.5 (1.2 – 6.9)	12.6 (2.8 – 33.2)	17.3 (5.2 – 38.6)	19.7 (12.9 – 28.1)
	Timbuktu	Goundam	Only RHF	93.9 (88.7 – 97.1)	79 (52.2 – 93.5)	76.2 (51.7 – 92.2)	78 (69.1 – 85.1)
	Timbuktu	Goundam	No ORT	2.6 (0.9 – 6)	8.4 (1.2 – 23.9)	6.4 (0.9 – 19.6)	2.4 (0.8 – 5.7)
	Timbuktu	Gourma-Rharous	Any ORS	5.7 (2.4 – 10.9)	13.1 (3.6 – 30.5)	14.4 (4.3 – 30.8)	29 (17.8 – 42.8)
	Timbuktu	Gourma-Rharous	Only RHF	90.8 (84.1 – 95.3)	77.8 (56.3 – 91.8)	78.3 (57.5 – 91.9)	67.2 (52.3 – 79.2)
	Timbuktu	Gourma-Rharous	No ORT	3.4 (1.1 – 7.3)	9.1 (1.8 – 23.2)	7.3 (1.3 – 19.6)	3.8 (1 – 8.9)
	Timbuktu	Niafunké	Any ORS	3.3 (1.2 – 6.4)	12.4 (2.6 – 31.7)	19.4 (5.8 – 43.4)	23.4 (16.4 – 30.9)
	Timbuktu	Niafunké	Only RHF	92.9 (88.4 – 96.3)	78.6 (54 – 93.6)	72.6 (45.7 – 90.1)	74.4 (66.5 – 81.7)
	Timbuktu	Niafunké	No ORT	3.8 (1.6 – 6.9)	9.1 (1.2 – 26.3)	8 (1.1 – 22.6)	2.2 (0.7 – 4.9)
	Timbuktu	Tombouctou	Any ORS	4.8 (2 – 9.7)	13.9 (3.2 – 33.8)	14 (4.1 – 31.6)	22.7 (14.6 – 31.9)
	Timbuktu	Tombouctou	Only RHF	92.3 (86.2 – 96.3)	77.4 (51.9 – 92.7)	80.4 (58.8 – 93.7)	73.5 (63.1 – 81.7)
	Timbuktu	Tombouctou	No ORT	2.8 (0.8 – 6.6)	8.7 (1.4 – 25)	5.6 (1 – 17.5)	3.8 (1.3 – 7.9)
**Southern**	Kayes	Bafoulabé	Any ORS	10.8 (6.3 – 17)	15.9 (3.5 – 35.8)	18.7 (5.1 – 39.3)	16.9 (9.7 – 26)
	Kayes	Bafoulabé	Only RHF	44.4 (34.9 – 53.8)	73.9 (49.1 – 91.4)	72.2 (47.6 – 89.1)	81.9 (72.1 – 89.6)
	Kayes	Bafoulabé	No ORT	44.8 (35.8 – 54)	10.1 (1.6 – 26.8)	9 (1.5 – 24.3)	1.2 (0.2 – 3.5)
	Kayes	Diéma	Any ORS	6.9 (3.7 – 11.9)	15.3 (3.3 – 36.3)	17 (4.3 – 39.1)	7.5 (4.1 – 12.1)
	Kayes	Diéma	Only RHF	66.2 (58 – 74.8)	75.1 (50.7 – 92.1)	76 (50.9 – 91.7)	91.4 (86.3 – 95.1)
	Kayes	Diéma	No ORT	26.9 (19.5 – 34.7)	9.6 (1.4 – 26.9)	7 (0.8 – 21.5)	1.2 (0.2 – 3.2)
	Kayes	Kayes	Any ORS	24.2 (15.6 – 33.4)	17.3 (3.9 – 40.5)	19.4 (5.7 – 40.4)	17.1 (9 – 27.6)
	Kayes	Kayes	Only RHF	48.7 (38 – 60.7)	74.2 (46.4 – 91.6)	73.8 (49.1 – 91.7)	81.3 (70.1 – 89.7)
	Kayes	Kayes	No ORT	27.1 (18 – 36.5)	8.5 (1.2 – 23.5)	6.7 (0.9 – 20.2)	1.6 (0.3 – 5.5)
	Kayes	Kita	Any ORS	6.2 (3.2 – 11)	17 (4.6 – 36.9)	19.5 (5.6 – 39.3)	17.5 (11.7 – 25.1)
	Kayes	Kita	Only RHF	62.2 (52.7 – 70.9)	72.7 (47.1 – 91.1)	71.8 (46.2 – 88.6)	81.3 (73.6 – 87.4)
	Kayes	Kita	No ORT	31.6 (23.3 – 40.5)	10.3 (1.8 – 26.7)	8.6 (1.6 – 22.9)	1.3 (0.3 – 3.6)
	Kayes	Kéniéba	Any ORS	10.5 (4.6 – 19.1)	16 (3.2 – 39.8)	18.4 (4.9 – 39.5)	18.8 (11.3 – 29.5)
	Kayes	Kéniéba	Only RHF	62 (49.2 – 73.7)	73.2 (44.7 – 91.2)	69.8 (42.4 – 89.4)	79.6 (68.6 – 87.5)
	Kayes	Kéniéba	No ORT	27.5 (17.2 – 39.4)	10.8 (1.5 – 29.8)	11.8 (2 – 30.9)	1.6 (0.3 – 4.5)
	Kayes	Nioro	Any ORS	9.8 (4.9 – 16.4)	14.7 (3.4 – 35.5)	18.7 (5 – 40.3)	5.4 (2.5 – 10.2)

	Kayes	Nioro	Only RHF	76.3 (67.2 – 83.7)	76.5 (51.3 – 92.8)	74.6 (45.5 – 92.2)	93.4 (87.8 – 96.7)
	Kayes	Nioro	No ORT	13.9 (8.2 – 21.2)	8.8 (1.1 – 25)	6.7 (0.7 – 21.3)	1.3 (0.2 – 3.5)
	Kayes	Yélimané	Any ORS	18.4 (10.5 – 27.7)	15 (2.9 – 38.8)	18.8 (4.5 – 41.9)	8.2 (3.6 – 15.1)
	Kayes	Yélimané	Only RHF	70.4 (59.1 – 79.9)	76.9 (50.1 – 93.3)	75 (47.1 – 92.2)	90.7 (83 – 95.7)
	Kayes	Yélimané	No ORT	11.1 (5.6 – 20.2)	8.1 (1 – 24)	6.3 (0.8 – 20.9)	1.1 (0.2 – 3.3)
	Koulikoro	Banamba	Any ORS	9.3 (5.3 – 14.9)	15.8 (3.4 – 38.7)	17.1 (4.1 – 39.8)	18.4 (9.5 – 31)
	Koulikoro	Banamba	Only RHF	48.5 (38.6 – 59.4)	73.9 (46 – 92.3)	75 (48.6 – 92.6)	78 (63.9 – 87.5)
	Koulikoro	Banamba	No ORT	42.1 (32.8 – 52.4)	10.3 (1.1 – 29.7)	7.8 (1 – 23.1)	3.6 (0.9 – 9.6)
	Koulikoro	Dioïla	Any ORS	17.7 (11 – 25.5)	16.1 (3.6 – 37.7)	19.5 (5.2 – 43.7)	18.4 (11.8 – 26.8)
	Koulikoro	Dioïla	Only RHF	60 (50.2 – 69.4)	74.2 (46.2 – 91.8)	71.9 (45 – 91.7)	78.2 (68.5 – 85.7)
	Koulikoro	Dioïla	No ORT	22.3 (15.1 – 30.6)	9.7 (1.2 – 27.5)	8.6 (1.3 – 23.7)	3.5 (1.1 – 8)
	Koulikoro	Kangaba	Any ORS	8.6 (4.4 – 14.8)	18.1 (4.2 – 42.8)	23.6 (6 – 49.2)	23.6 (12.3 – 38)
	Koulikoro	Kangaba	Only RHF	80.8 (71.3 – 87.9)	72 (41.5 – 92.1)	65.2 (35.5 – 88.7)	73 (57.3 – 85.4)
	Koulikoro	Kangaba	No ORT	10.6 (5.5 – 17.6)	9.9 (1 – 30.5)	11.2 (1.6 – 29.6)	3.4 (0.6 – 9)
	Koulikoro	Kati	Any ORS	22.3 (16.7 – 27.6)	20.9 (5.1 – 45.9)	23.7 (7.2 – 48.4)	23.3 (18.7 – 28.3)
	Koulikoro	Kati	Only RHF	51.1 (44.9 – 57.2)	69.3 (38.3 – 90.8)	65.9 (36.1 – 87.3)	75.8 (70.7 – 80.4)
	Koulikoro	Kati	No ORT	26.6 (20.9 – 32.3)	9.8 (1.3 – 29.6)	10.5 (1.3 – 28.1)	1 (0.3 – 2.4)
	Koulikoro	Kolokani	Any ORS	13.2 (7.8 – 19.3)	17.5 (3.6 – 40.1)	17.5 (4.2 – 39.1)	16.2 (9.1 – 27.2)
	Koulikoro	Kolokani	Only RHF	60.3 (51.1 – 69.6)	72.3 (46 – 91.3)	73.2 (46.8 – 90.6)	82.2 (71 – 89.9)
	Koulikoro	Kolokani	No ORT	26.5 (19.3 – 34.5)	10.2 (1.5 – 30)	9.3 (1.4 – 26.2)	1.5 (0.3 – 4.6)
	Koulikoro	Koulikoro	Any ORS	16.1 (10.4 – 23)	17.2 (3.6 – 39.8)	19 (5.5 – 43.5)	19.9 (12.2 – 29.5)
	Koulikoro	Koulikoro	Only RHF	40.7 (32.2 – 50.4)	72.8 (43.9 – 92.3)	73.8 (46.1 – 91.2)	77.2 (67 – 85.3)
	Koulikoro	Koulikoro	No ORT	43.3 (34.9 – 52.3)	10 (1.3 – 29.7)	7.2 (0.9 – 21.2)	2.9 (0.8 – 7)
	Koulikoro	Nara	Any ORS	7 (3 – 12.3)	13.5 (3.4 – 32.4)	14.4 (3.8 – 31.8)	11 (5 – 20.1)
	Koulikoro	Nara	Only RHF	81.4 (72.5 – 88.4)	77.5 (54.2 – 92.4)	76.5 (55.2 – 91.8)	84.8 (73.8 – 92.3)
	Koulikoro	Nara	No ORT	11.6 (6.4 – 18.8)	9 (1.5 – 25.1)	9 (1.7 – 23.1)	4.2 (1.1 – 10.4)
	Sikasso	Bougouni	Any ORS	13.7 (8.6 – 19.8)	16.5 (4.1 – 36.8)	21.4 (6.6 – 43.6)	24.4 (14.5 – 36.1)
	Sikasso	Bougouni	Only RHF	72.2 (63.6 – 79.3)	73.9 (47.4 – 90.8)	64.7 (40.1 – 85.9)	73.5 (60.4 – 83.7)
	Sikasso	Bougouni	No ORT	14.1 (8.8 – 20.6)	9.6 (1.4 – 26.9)	13.9 (3.2 – 32.3)	2.1 (0.4 – 6.1)

	Sikasso	Kadiolo	Any ORS	27.4 (19.3 – 36.6)	17.8 (3.3 – 43.8)	17.2 (4 – 39.4)	23.8 (11.6 – 39.3)
	Sikasso	Kadiolo	Only RHF	59.7 (49.9 – 69.4)	72.2 (39.7 – 91.7)	70.4 (42.5 – 90.4)	74 (57.7 – 87.2)
	Sikasso	Kadiolo	No ORT	13 (7.4 – 19.6)	10.1 (1 – 30.2)	12.3 (2.1 – 33.8)	2.2 (0.3 – 8.1)
	Sikasso	Kolondiéba	Any ORS	19 (11.9 – 28.7)	15.7 (3.1 – 38.8)	20.3 (5.1 – 43.8)	27.3 (15.8 – 41.9)
	Sikasso	Kolondiéba	Only RHF	67.7 (56.6 – 77)	75.4 (46.7 – 93)	66.5 (38.1 – 88.5)	70.6 (55.4 – 83.1)
	Sikasso	Kolondiéba	No ORT	13.3 (7.4 – 20.8)	9 (1 – 27.4)	13.2 (2.4 – 33.9)	2.1 (0.3 – 7.1)
	Sikasso	Koutiala	Any ORS	18.2 (11.6 – 26.3)	17.2 (3.3 – 42.2)	19.6 (4.6 – 44.3)	28.9 (18.8 – 40.3)
	Sikasso	Koutiala	Only RHF	70.2 (60.5 – 78.8)	73 (43.6 – 92)	68.1 (39.6 – 88.8)	63.1 (50.7 – 73.6)
	Sikasso	Koutiala	No ORT	11.7 (6.5 – 18.6)	9.8 (1.1 – 28)	12.4 (2.1 – 32.1)	8 (3.4 – 15.7)
	Sikasso	Sikasso	Any ORS	16.7 (10.4 – 24.5)	17.2 (3.8 – 42.6)	20.2 (5.4 – 43.5)	20.9 (13.2 – 31.8)
	Sikasso	Sikasso	Only RHF	72.4 (63.3 – 79.9)	72.8 (45 – 91.7)	63.2 (34 – 85.6)	77.2 (66.3 – 85.6)
	Sikasso	Sikasso	No ORT	10.9 (6.3 – 16.9)	10 (1.2 – 29.9)	16.6 (3.4 – 40.4)	1.9 (0.4 – 5)
	Sikasso	Yanfolila	Any ORS	7.6 (3.5 – 13.7)	17.2 (4.1 – 38.8)	24.7 (7.4 – 49.7)	24 (11.4 – 39.4)
	Sikasso	Yanfolila	Only RHF	85.9 (76.7 – 91.9)	72.7 (43.4 – 91.6)	61.6 (32.9 – 85)	72.8 (55.4 – 86.3)
	Sikasso	Yanfolila	No ORT	6.5 (2.8 – 11.6)	10.1 (1.2 – 30)	13.7 (2.5 – 35)	3.2 (0.5 – 9.4)
	Sikasso	Yorosso	Any ORS	13.5 (7.3 – 22.4)	16.8 (3.1 – 43.4)	19.6 (4.7 – 47.1)	26.7 (15.7 – 39.6)
	Sikasso	Yorosso	Only RHF	80.5 (70.4 – 88.2)	73 (42.1 – 92.8)	63.9 (33.2 – 87.7)	70.6 (57.1 – 82.5)
	Sikasso	Yorosso	No ORT	6 (2.2 – 11.7)	10.2 (0.9 – 31.4)	16.5 (3.1 – 40.6)	2.7 (0.6 – 7.5)
	Ségou	Barouéli	Any ORS	16 (9.5 – 23.9)	15.2 (3 – 37.3)	20.8 (5.6 – 45.5)	19.6 (11.9 – 28.6)
	Ségou	Barouéli	Only RHF	55.9 (45.1 – 66.3)	75.1 (46.5 – 93.4)	72.2 (43.8 – 91.3)	67.5 (55.8 – 76.9)
	Ségou	Barouéli	No ORT	28.1 (19.9 – 37.4)	9.7 (0.9 – 30.8)	7 (1 – 21.5)	12.9 (6.8 – 20.8)
	Ségou	Bla	Any ORS	21.5 (13.2 – 31.8)	15.7 (3.3 – 38.3)	16.9 (3.8 – 39.4)	25.2 (16.9 – 33.5)
	Ségou	Bla	Only RHF	66.6 (54.4 – 76.4)	74.4 (48.3 – 92.7)	73.7 (44.5 – 91.4)	55.3 (46.7 – 64.7)
	Ségou	Bla	No ORT	12 (5.8 – 20.1)	9.9 (1 – 28.5)	9.4 (1.6 – 25.7)	19.4 (12.8 – 28.2)
	Ségou	Macina	Any ORS	10.7 (5.4 – 18.3)	15.2 (3.1 – 38.9)	17.7 (4 – 40.4)	20.8 (11.9 – 32)
	Ségou	Macina	Only RHF	81.4 (71.6 – 88.8)	74.8 (45 – 92.8)	71.7 (44.4 – 91.1)	72.6 (59.7 – 83.5)
	Ségou	Macina	No ORT	7.9 (3.4 – 14.2)	10 (1.2 – 29)	10.6 (1.7 – 29.9)	6.6 (2.4 – 14)
	Ségou	Niono	Any ORS	9.8 (4.4 – 17.9)	15.9 (3.1 – 41.6)	21.6 (6.1 – 46.1)	35.2 (22.3 – 49.8)
	Ségou	Niono	Only RHF	78.1 (67.2 – 87.1)	75 (44.1 – 92.4)	71.1 (43.9 – 90.5)	55.6 (40.1 – 69.8)

	Ségou	Niono	No ORT	12.1 (5.8 – 21)	9.1 (1 – 29.1)	7.2 (0.9 – 22.3)	9.2 (3.3 – 18.6)
	Ségou	San	Any ORS	13.6 (7.9 – 20.6)	15.9 (3.1 – 39.9)	16 (3.3 – 36.9)	16.2 (10.3 – 23.7)
	Ségou	San	Only RHF	80.9 (72.8 – 87.5)	74 (45.8 – 92.9)	73.3 (46.3 – 91.1)	80.9 (73 – 87.3)
	Ségou	San	No ORT	5.5 (2.4 – 9.9)	10.1 (1 – 29.9)	10.8 (1.8 – 28.5)	2.8 (1 – 6.3)
	Ségou	Ségou	Any ORS	17.2 (9.8 – 27.3)	16 (3.5 – 38)	20.5 (5.7 – 45.3)	21.7 (15.4 – 29.3)
	Ségou	Ségou	Only RHF	70.4 (56.9 – 80.1)	74.1 (46.3 – 92.4)	70.7 (42.2 – 90.7)	56.1 (48.3 – 64.5)
	Ségou	Ségou	No ORT	12.4 (6.2 – 20.7)	9.9 (0.9 – 31.2)	8.8 (1.3 – 25.6)	22.1 (15.3 – 29.1)
	Ségou	Tominian	Any ORS	7.8 (4.1 – 13.9)	14.5 (3.1 – 35.8)	15.5 (3.4 – 36.8)	15 (9 – 23.2)
	Ségou	Tominian	Only RHF	88.3 (81.1 – 93)	75.6 (50.1 – 92.6)	73.4 (45.6 – 91)	83 (74.5 – 89.6)
	Ségou	Tominian	No ORT	4 (1.6 – 7.5)	9.9 (1.2 – 29)	11 (1.7 – 28.8)	2 (0.6 – 4.7)

c) Senegal

Coverage estimates by Region, Department, Treatment (Any ORS, Only RHF, and No ORT) for 2000, 2006, 2012, and 2017. Estimates are shown as percentages in the format: mean (2.5%–97.5% uncertainty interval). Groups Northern, Central, and Southern correspond to regions shown in Figure 4.

Group	Region	Department	Treatment	2000	2006	2012	2017
Northern	Louga	Kébémer	Any ORS	11 (7.4 – 15.5)	22.2 (15.8 – 28.9)	24.1 (18.1 – 31.6)	24.4 (19.1 – 30.2)
	Louga	Kébémer	Only RHF	59.2 (52.2 – 65.7)	71.6 (63.6 – 78.9)	72.2 (64.3 – 79)	73.2 (67 – 78.9)
	Louga	Kébémer	No ORT	29.8 (24.1 – 36)	6.2 (3.2 – 10.5)	3.7 (2 – 6.1)	2.3 (1.3 – 3.8)
	Louga	Linguère	Any ORS	10.3 (6.8 – 14.3)	18.8 (13.6 – 24.9)	19.7 (14.6 – 25.6)	23.6 (19 – 28.9)
	Louga	Linguère	Only RHF	62.9 (56.3 – 69.4)	75.7 (68.8 – 81.4)	77.1 (70.5 – 83)	74.3 (68.6 – 79.4)
	Louga	Linguère	No ORT	26.8 (21.2 – 32.9)	5.6 (3.1 – 9)	3.2 (1.7 – 5.2)	2.1 (1.2 – 3.6)
	Louga	Louga	Any ORS	11.3 (7.4 – 15.9)	21.8 (15.6 – 28.7)	23.9 (17.7 – 31)	25.4 (19.9 – 31.1)
	Louga	Louga	Only RHF	62.4 (55.3 – 69)	72.1 (64.4 – 79.6)	72.5 (64.5 – 79.5)	72.5 (66.5 – 78.2)
	Louga	Louga	No ORT	26.4 (20.3 – 32.4)	6.1 (3.1 – 10.3)	3.6 (1.8 – 6)	2.1 (1.1 – 3.5)
	Matam	Kanel	Any ORS	12 (7.6 – 17.7)	22.5 (16.5 – 29.1)	22.3 (16.4 – 29)	27.8 (22.1 – 33.8)
	Matam	Kanel	Only RHF	56.8 (48.8 – 64.2)	71.4 (64.2 – 78.1)	74.8 (68 – 81.3)	70.5 (64.1 – 76.4)
	Matam	Kanel	No ORT	31.2 (24.5 – 38.7)	6.1 (3.4 – 10.2)	3 (1.6 – 5.2)	1.7 (0.9 – 2.8)
	Matam	Matam	Any ORS	11.5 (7.1 – 16.7)	23.1 (16.8 – 30.3)	22 (16.3 – 28.6)	28 (22.1 – 34.3)
	Matam	Matam	Only RHF	60.1 (52.2 – 67.7)	71.9 (63.9 – 79.4)	75.2 (68.1 – 81.7)	70.2 (63.6 – 76.6)
	Matam	Matam	No ORT	28.4 (21.4 – 35.4)	5 (2.6 – 8.6)	2.8 (1.4 – 4.8)	1.7 (0.9 – 2.9)
	Matam	Ranérou Ferlo	Any ORS	11.4 (7.5 – 16.3)	19 (13.9 – 24.4)	20.2 (15.5 – 26.1)	24.9 (20 – 30.5)
	Matam	Ranérou Ferlo	Only RHF	58.4 (51.3 – 65.6)	75.5 (68.5 – 81.6)	76.8 (70.5 – 81.9)	73.2 (67.2 – 78.7)
	Matam	Ranérou Ferlo	No ORT	30.2 (23.4 – 37.2)	5.4 (3 – 8.5)	3 (1.6 – 4.9)	1.9 (1 – 3)
	Saint-Louis	Dagana	Any ORS	11 (6.9 – 16)	21.9 (15.9 – 28.4)	24.7 (18.6 – 31.8)	27.8 (21.5 – 34.5)
	Saint-Louis	Dagana	Only RHF	61.2 (53.8 – 68.4)	72.3 (64.7 – 78.6)	71.8 (63.7 – 78.7)	70.3 (63.2 – 77)
	Saint-Louis	Dagana	No ORT	27.7 (21.6 – 34.2)	5.8 (3.3 – 9.2)	3.5 (1.9 – 5.6)	1.9 (1 – 3.3)
	Saint-Louis	Podor	Any ORS	10.8 (7.3 – 15.4)	20.6 (15.2 – 26.7)	22.1 (16.8 – 28.4)	26.7 (21.4 – 32.5)
	Saint-Louis	Podor	Only RHF	61.5 (54.5 – 67.7)	74.2 (66.8 – 80.1)	75 (68.2 – 80.8)	71.5 (65.3 – 77.1)
	Saint-Louis	Podor	No ORT	27.6 (21.7 – 34)	5.2 (3 – 8.6)	3 (1.7 – 4.7)	1.8 (1 – 3)
	Saint-Louis	Saint-Louis	Any ORS	11.7 (6.8 – 18)	22.6 (15 – 31.1)	24.6 (16.7 – 33.4)	27.1 (19.7 – 35.2)

	Saint-Louis	Saint-Louis	Only RHF	61.7 (52 – 71.1)	71.7 (61.4 – 80.2)	71.5 (61.6 – 80.2)	70.9 (61.9 – 78.4)
	Saint-Louis	Saint-Louis	No ORT	26.5 (18.9 – 34.9)	5.7 (2.8 – 10.1)	3.9 (1.9 – 7.3)	2 (1 – 3.6)
**Central**	Dakar	Dakar	Any ORS	14.8 (8.2 – 23.3)	24 (14.9 – 33.7)	24.9 (15.8 – 35.8)	29.1 (20.2 – 39.2)
	Dakar	Dakar	Only RHF	47.3 (34.5 – 59.7)	68.4 (56.6 – 79.5)	70.8 (58.4 – 81.1)	67.8 (56.9 – 77.5)
	Dakar	Dakar	No ORT	38 (26.9 – 50.6)	7.6 (3.5 – 13.5)	4.3 (1.9 – 8.5)	3.1 (1.5 – 5.5)
	Dakar	Guédiawaye	Any ORS	13.2 (6.1 – 21.9)	22.4 (13.3 – 33.1)	23 (13.8 – 34.7)	28.9 (18 – 42.6)
	Dakar	Guédiawaye	Only RHF	48 (34.7 – 63)	69.2 (56 – 80.9)	72 (58.1 – 83)	66.9 (52.1 – 78.7)
	Dakar	Guédiawaye	No ORT	38.8 (26.8 – 50.3)	8.4 (3.7 – 15)	5 (2.1 – 10)	4.3 (1.9 – 8)
	Dakar	Pikine	Any ORS	13.7 (7.8 – 21.4)	24.1 (16.1 – 33)	25 (16.5 – 35)	31.1 (21.6 – 42)
	Dakar	Pikine	Only RHF	47.2 (36.7 – 58.1)	67.4 (56.3 – 77.3)	70 (58.9 – 79.5)	64.9 (53.7 – 75)
	Dakar	Pikine	No ORT	39 (29.6 – 49.1)	8.5 (4.1 – 14.5)	5 (2.4 – 9.2)	4 (2.1 – 7)
	Dakar	Rufisque	Any ORS	11.9 (7.2 – 18.1)	25.2 (16.7 – 34.4)	27.8 (19.1 – 36.9)	31.8 (23.7 – 40.6)
	Dakar	Rufisque	Only RHF	46.8 (37.5 – 56.4)	64.1 (53.8 – 73.3)	65.9 (55.6 – 75.8)	63.7 (54.2 – 72.5)
	Dakar	Rufisque	No ORT	41.3 (32.8 – 50.3)	10.7 (5.4 – 17.8)	6.3 (3.2 – 10.8)	4.5 (2.5 – 7.3)
	Diourbel	Bambey	Any ORS	9.9 (6.4 – 14.1)	20.1 (13.7 – 28)	22.4 (15.8 – 29.8)	24.9 (19 – 31.1)
	Diourbel	Bambey	Only RHF	53.1 (45.8 – 60.9)	73.6 (64.3 – 81.3)	73.8 (65.9 – 81)	72 (65.3 – 78.3)
	Diourbel	Bambey	No ORT	37.1 (30.9 – 43.5)	6.3 (3.2 – 10.4)	3.7 (1.8 – 6.5)	3.1 (1.7 – 5.1)
	Diourbel	Diourbel	Any ORS	10.1 (6.6 – 14.4)	20.3 (14.1 – 28.2)	22 (15.5 – 29.3)	24.2 (18.4 – 31.1)
	Diourbel	Diourbel	Only RHF	54.1 (46.4 – 61.6)	72.9 (64.1 – 80.4)	74.3 (66.3 – 81.7)	72.6 (65.4 – 78.8)
	Diourbel	Diourbel	No ORT	35.8 (29.1 – 42.8)	6.7 (3.3 – 11.5)	3.7 (1.8 – 6.5)	3.2 (1.8 – 5.4)
	Diourbel	Mbacké	Any ORS	9.5 (6.2 – 14)	19.1 (12.4 – 26.5)	20.1 (13.7 – 27.7)	22 (16.7 – 28.2)
	Diourbel	Mbacké	Only RHF	56.7 (49.4 – 64.3)	74.3 (65.6 – 82.3)	76.1 (67.5 – 83.3)	74.9 (67.9 – 80.9)
	Diourbel	Mbacké	No ORT	33.8 (27.2 – 40.7)	6.6 (3.3 – 11.2)	3.8 (1.9 – 6.9)	3.2 (1.7 – 5.4)
	Fatick	Fatick	Any ORS	13.2 (8.9 – 18.1)	23.1 (16.3 – 30.2)	24.8 (18.4 – 32)	28.3 (22.3 – 34.8)
	Fatick	Fatick	Only RHF	52.5 (45.7 – 59.9)	71.2 (63.3 – 78.8)	72.1 (64.5 – 79)	68.9 (62.1 – 75.3)
	Fatick	Fatick	No ORT	34.3 (27.8 – 40.6)	5.7 (3 – 9.5)	3.2 (1.6 – 5.6)	2.8 (1.6 – 4.3)
	Fatick	Foundiougne	Any ORS	15.8 (10.9 – 21.6)	24.4 (17.9 – 32)	26.9 (20 – 34.9)	29.7 (23.6 – 36.7)
	Fatick	Foundiougne	Only RHF	51.3 (43.7 – 58.8)	69.4 (61.2 – 77.3)	69.7 (61 – 77.4)	67.8 (60.1 – 74.5)
	Fatick	Foundiougne	No ORT	32.9 (26 – 40)	6.2 (3.2 – 10.3)	3.4 (1.8 – 5.7)	2.5 (1.4 – 4)

	Fatick	Gossas	Any ORS	11.1 (7.4 – 16)	20 (13.3 – 27.9)	20.9 (14.9 – 27.6)	23.9 (18.3 – 30.1)
	Fatick	Gossas	Only RHF	54.8 (47.3 – 62.7)	73.7 (64.8 – 81.7)	75.7 (68.3 – 82.5)	73.2 (66.2 – 79.5)
	Fatick	Gossas	No ORT	34.1 (27.2 – 40.8)	6.3 (3.3 – 10.4)	3.5 (1.7 – 6.1)	2.9 (1.6 – 5)
	Kaffrine	Birkilane	Any ORS	14.4 (9.5 – 19.9)	22.8 (15.8 – 30.7)	24.1 (17.3 – 31.7)	25.6 (19.8 – 32.4)
	Kaffrine	Birkilane	Only RHF	53.1 (45.5 – 61.4)	70.6 (61.9 – 78.7)	72.4 (63.8 – 79.9)	72.1 (64.9 – 78.2)
	Kaffrine	Birkilane	No ORT	32.5 (25.4 – 39.6)	6.6 (3.4 – 10.8)	3.5 (1.8 – 6.3)	2.4 (1.3 – 3.8)
	Kaffrine	Kaffrine	Any ORS	13.7 (9.1 – 19)	21.4 (14.9 – 29)	23 (16.5 – 29.8)	24.5 (19.4 – 30.4)
	Kaffrine	Kaffrine	Only RHF	53.7 (45.5 – 60.9)	71.9 (63.5 – 79)	73.5 (65.6 – 80.7)	73.1 (66.8 – 78.9)
	Kaffrine	Kaffrine	No ORT	32.6 (25.4 – 39.8)	6.8 (3.6 – 11)	3.6 (1.9 – 6.2)	2.3 (1.4 – 3.8)
	Kaffrine	Koungheul	Any ORS	13.7 (8.9 – 19.9)	20.8 (14.9 – 28)	22.9 (16.9 – 30)	25 (19.5 – 31.3)
	Kaffrine	Koungheul	Only RHF	53 (44.5 – 61.5)	73 (65.4 – 79.6)	73.7 (65.9 – 80.5)	73 (66.3 – 78.9)
	Kaffrine	Koungheul	No ORT	33.4 (25.9 – 40.6)	6.2 (3.2 – 10.4)	3.3 (1.7 – 5.9)	2 (1.1 – 3.3)
	Kaffrine	Malème Hodar	Any ORS	13 (8.7 – 18.7)	20.7 (14.3 – 28)	22.2 (15.9 – 29)	24.2 (19.1 – 30.1)
	Kaffrine	Malème Hodar	Only RHF	54.9 (47 – 63.1)	72.8 (65.1 – 80.1)	74.4 (66.6 – 81.4)	73.5 (67.3 – 79.1)
	Kaffrine	Malème Hodar	No ORT	32.1 (24.8 – 39)	6.4 (3.4 – 10.4)	3.5 (1.8 – 6.1)	2.3 (1.3 – 3.7)
	Kaolack	Guinguinéo	Any ORS	13.7 (9 – 18.9)	22.8 (15.8 – 30.4)	23.9 (17.2 – 31.6)	26.8 (21 – 33.7)
	Kaolack	Guinguinéo	Only RHF	52.6 (44.9 – 61)	71 (62.3 – 79)	72.7 (64.7 – 80.5)	70.4 (63 – 77)
	Kaolack	Guinguinéo	No ORT	33.6 (26.7 – 41)	6.2 (3.3 – 10.6)	3.4 (1.7 – 6)	2.8 (1.5 – 4.7)
	Kaolack	Kaolack	Any ORS	14.4 (9.9 – 20.1)	24.1 (16.8 – 32.4)	25.9 (19.1 – 33.6)	28.5 (22.5 – 35.4)
	Kaolack	Kaolack	Only RHF	51.1 (43.1 – 59.2)	68.9 (60 – 77.3)	70.2 (61.8 – 78)	68.5 (61.2 – 75)
	Kaolack	Kaolack	No ORT	34.4 (27.4 – 42.3)	6.9 (3.7 – 11.5)	3.9 (2 – 6.7)	3.1 (1.7 – 4.9)
	Kaolack	Nioro du Rip	Any ORS	15 (10.2 – 20.8)	22.7 (15.9 – 29.8)	24.6 (17.9 – 31.7)	25.8 (20.1 – 31.6)
	Kaolack	Nioro du Rip	Only RHF	51.8 (44.3 – 59.6)	70.5 (62.4 – 78.6)	71.8 (63.8 – 78.8)	71.9 (65.7 – 77.8)
	Kaolack	Nioro du Rip	No ORT	33.2 (26 – 40.1)	6.8 (3.6 – 11)	3.6 (2 – 6.1)	2.4 (1.3 – 3.7)
	Kolda	Kolda	Any ORS	14.9 (9.9 – 21.4)	23.4 (16.6 – 30.4)	25.3 (18.7 – 32.8)	27 (21.6 – 33.3)
	Kolda	Kolda	Only RHF	47.3 (39 – 55.8)	70.7 (62.4 – 78.2)	71.2 (62.8 – 78.5)	71 (64.4 – 76.6)
	Kolda	Kolda	No ORT	37.8 (30.5 – 45.1)	6 (3.2 – 10.2)	3.5 (1.9 – 5.9)	2 (1.1 – 3.2)
	Kolda	Médina Yoro Foula	Any ORS	15.3 (10.5 – 21.3)	22.1 (16.3 – 28.9)	24.3 (18.3 – 31.9)	25.6 (20.2 – 31.6)
	Kolda	Médina Yoro Foula	Only RHF	49.5 (41.6 – 57.2)	71.9 (64.3 – 78.8)	72.3 (64.2 – 79.1)	72.5 (66.1 – 78.3)

	Kolda	Médina Yoro Foula	No ORT	35.2 (28.2 – 42.5)	5.9 (3.1 – 9.9)	3.4 (1.8 – 5.5)	1.9 (1 – 3.1)
	Kolda	Vélingara	Any ORS	14.2 (9.2 – 20.2)	22.7 (16 – 30.1)	24.3 (18.4 – 31.5)	26.3 (20.4 – 32.4)
	Kolda	Vélingara	Only RHF	48.5 (40.6 – 56.4)	71.9 (63.8 – 79.1)	72.2 (64.8 – 79)	71.7 (65.1 – 78.1)
	Kolda	Vélingara	No ORT	37.3 (30.2 – 45)	5.4 (2.8 – 8.9)	3.5 (1.9 – 5.9)	2 (1.1 – 3.5)
	Kédougou	Kédougou	Any ORS	14.5 (9.4 – 21.4)	23.4 (17.1 – 31)	25 (18.6 – 32.4)	26.8 (20.9 – 33.4)
	Kédougou	Kédougou	Only RHF	50.2 (41.6 – 59.2)	71 (63.1 – 77.9)	71.8 (63.9 – 78.5)	71.4 (64.5 – 77.5)
	Kédougou	Kédougou	No ORT	35.3 (27.4 – 43.3)	5.7 (3.1 – 9.2)	3.2 (1.8 – 5.2)	1.8 (0.9 – 3.2)
	Kédougou	Salémata	Any ORS	14.7 (9.6 – 22.4)	23.5 (16.8 – 31.7)	25.4 (18.8 – 33.7)	27.1 (21 – 35)
	Kédougou	Salémata	Only RHF	49.8 (40.4 – 58.9)	70.9 (62.3 – 78.4)	71.4 (62.7 – 78.6)	71.1 (63 – 77.6)
	Kédougou	Salémata	No ORT	35.4 (26.8 – 44.8)	5.6 (3 – 9.3)	3.2 (1.7 – 5.6)	1.8 (0.9 – 3.1)
	Kédougou	Saraya	Any ORS	14.6 (9.3 – 21.1)	22.8 (16.7 – 29.5)	24.6 (18.9 – 31.5)	26.5 (20.7 – 33.2)
	Kédougou	Saraya	Only RHF	50.1 (41.8 – 58.8)	71.4 (63.8 – 78)	72 (64.7 – 78.4)	71.6 (64.5 – 77.7)
	Kédougou	Saraya	No ORT	35.3 (27.1 – 43.6)	5.8 (3.1 – 9.3)	3.4 (1.9 – 5.7)	1.9 (1 – 3.2)
	Tambacounda	Bakel	Any ORS	12.9 (8.6 – 18.1)	21.2 (15.7 – 27.3)	22.7 (17.5 – 28.7)	25.5 (20.2 – 31.1)
	Tambacounda	Bakel	Only RHF	52.9 (45.4 – 60)	73.4 (66.3 – 79.5)	74.1 (67.3 – 79.9)	72.7 (66.9 – 78.2)
	Tambacounda	Bakel	No ORT	34.2 (27.4 – 41)	5.4 (3.1 – 8.7)	3.1 (1.8 – 5.2)	1.8 (1 – 3)
	Tambacounda	Goudiry	Any ORS	13.6 (9.3 – 18.8)	20.9 (15.8 – 26.5)	22.5 (17.6 – 28.2)	25.1 (20 – 30.4)
	Tambacounda	Goudiry	Only RHF	51 (43.6 – 57.7)	73.9 (67.8 – 79.8)	74.2 (68.3 – 79.8)	73 (67.3 – 78.4)
	Tambacounda	Goudiry	No ORT	35.4 (28.6 – 42.3)	5.2 (3 – 8.3)	3.2 (1.9 – 5.1)	1.9 (1.1 – 3)
	Tambacounda	Koupentoum	Any ORS	13.6 (9.3 – 19.3)	20.5 (15 – 27.3)	22.7 (17.1 – 29.5)	25.4 (19.8 – 31.3)
	Tambacounda	Koupentoum	Only RHF	51.7 (44.1 – 59.5)	73.8 (66.8 – 80.3)	74.1 (67 – 80.3)	72.6 (66.3 – 78.7)
	Tambacounda	Koupentoum	No ORT	34.6 (27.6 – 41.5)	5.7 (3.1 – 9.3)	3.2 (1.7 – 5.3)	2 (1.1 – 3.2)
	Tambacounda	Tambacounda	Any ORS	14 (9.7 – 19.2)	21.7 (16.2 – 27.7)	23.3 (18.4 – 29.1)	25.6 (20 – 30.8)
	Tambacounda	Tambacounda	Only RHF	50 (42.9 – 56.8)	73.1 (66 – 79.3)	73.5 (66.9 – 78.9)	72.5 (66.6 – 78.2)
	Tambacounda	Tambacounda	No ORT	35.9 (29.2 – 42.8)	5.2 (2.9 – 8.3)	3.2 (1.9 – 5.1)	1.9 (1.1 – 3.1)
	Thiès	Mbour	Any ORS	12.7 (8.6 – 18.2)	22.4 (15.7 – 29.9)	24.3 (17.7 – 31.9)	27.8 (21.7 – 34.5)
	Thiès	Mbour	Only RHF	49.8 (42 – 57.8)	71.1 (63.1 – 78.8)	72 (64.1 – 79.4)	69.3 (62.2 – 75.4)
	Thiès	Mbour	No ORT	37.5 (30.5 – 44.4)	6.5 (3.5 – 10.6)	3.7 (1.9 – 6.5)	2.9 (1.7 – 4.7)
	Thiès	Thiès	Any ORS	10.8 (7.1 – 15.4)	21.3 (14.3 – 29.4)	23.7 (16.9 – 32.2)	26.7 (20.5 – 33.6)

	Thiès	Thiès	Only RHF	50.9 (43 – 58.2)	71.8 (62.6 – 80.2)	72.3 (63 – 79.8)	70.1 (63.1 – 76.7)
	Thiès	Thiès	No ORT	38.3 (31.3 – 45.3)	6.9 (3.6 – 11.2)	4 (2 – 7)	3.1 (1.7 – 5.1)
	Thiès	Tivaouane	Any ORS	11 (7.2 – 15.8)	21.9 (15.4 – 29)	24.3 (18 – 31.9)	26 (20.2 – 32.1)
	Thiès	Tivaouane	Only RHF	54.7 (47.4 – 61.5)	71.9 (63.4 – 79.6)	72 (64 – 79.1)	71.5 (64.9 – 78)
	Thiès	Tivaouane	No ORT	34.3 (27.8 – 40.9)	6.2 (3.4 – 10.1)	3.7 (1.9 – 6.4)	2.5 (1.4 – 4.1)
**Southern**	Sédhiou	Bounkiling	Any ORS	17.2 (12 – 23.5)	22.6 (16.1 – 30.1)	25.2 (18.8 – 33.1)	26.7 (21 – 33.1)
	Sédhiou	Bounkiling	Only RHF	47.5 (39.8 – 55.2)	70.4 (62 – 78.3)	71.5 (63.3 – 78.9)	71.5 (65 – 77.6)
	Sédhiou	Bounkiling	No ORT	35.3 (27.9 – 42.7)	7 (3.7 – 11.5)	3.2 (1.7 – 5.2)	1.8 (0.9 – 3.1)
	Sédhiou	Goudomp	Any ORS	16.1 (11.1 – 22.7)	22.2 (15.6 – 29.5)	25.1 (18.4 – 33.5)	27.1 (21.2 – 33.4)
	Sédhiou	Goudomp	Only RHF	46.2 (37.9 – 54.6)	70.8 (62.4 – 78.7)	71.5 (62.6 – 79.1)	70.9 (64 – 77.3)
	Sédhiou	Goudomp	No ORT	37.8 (30 – 45.8)	7 (3.6 – 11.4)	3.5 (1.8 – 5.8)	2 (1.1 – 3.4)
	Sédhiou	Sédhiou	Any ORS	16.8 (11.8 – 23.1)	22.8 (16.4 – 30)	25.4 (18.7 – 33.1)	27.6 (21.9 – 33.7)
	Sédhiou	Sédhiou	Only RHF	45.8 (38.1 – 53.6)	69.9 (61.5 – 77.3)	71.1 (62.8 – 78.4)	70.5 (63.9 – 76.5)
	Sédhiou	Sédhiou	No ORT	37.5 (30 – 45.3)	7.3 (4.1 – 12)	3.4 (1.9 – 5.7)	1.9 (1.1 – 3.2)
	Ziguinchor	Bignona	Any ORS	21.6 (16.1 – 28)	26.1 (19.2 – 33.7)	28.7 (21.9 – 36.8)	31.1 (25 – 37.7)
	Ziguinchor	Bignona	Only RHF	42.4 (34.8 – 50.4)	66.7 (58.3 – 74.3)	67.9 (59.2 – 75.4)	66.8 (59.7 – 73.1)
	Ziguinchor	Bignona	No ORT	35.9 (28.9 – 43.3)	7.3 (4 – 11.4)	3.5 (1.8 – 5.8)	2.1 (1.2 – 3.5)
	Ziguinchor	Oussouye	Any ORS	21.5 (14.7 – 29.5)	26.2 (18.5 – 34.9)	29.4 (21.5 – 38.2)	32.4 (24.4 – 40.9)
	Ziguinchor	Oussouye	Only RHF	42.7 (33.2 – 52.1)	66.8 (57.7 – 75.5)	66.5 (56.3 – 75.2)	65.2 (56.1 – 73.8)
	Ziguinchor	Oussouye	No ORT	35.8 (27.2 – 45.2)	7 (3.5 – 11.7)	4.2 (2 – 7.3)	2.3 (1.2 – 4.1)
	Ziguinchor	Ziguinchor	Any ORS	20.4 (14.5 – 27.7)	24.9 (17.1 – 33.3)	28.6 (20.9 – 37.6)	31.9 (24.9 – 40)
	Ziguinchor	Ziguinchor	Only RHF	42.4 (32.7 – 51.3)	67.5 (58.1 – 76.9)	67.3 (57.6 – 75.6)	65.7 (57.1 – 73.1)
	Ziguinchor	Ziguinchor	No ORT	37.2 (28.9 – 46.4)	7.6 (4 – 12.9)	4 (2.1 – 7.1)	2.4 (1.3 – 4.1)

Additional file 1: Table S11. Changes in oral rehydration therapies in three time periods.
Rates of change over time in oral rehydration therapies in a) Sierra Leone, b) Mali, and c) Senegal.

a) Sierra Leone

Average yearly change in treatment coverage by Province, District, Treatment (Any ORS, Only RHF, and No ORT) for three time periods. Estimates are shown as percentage change per year in the format: mean (2.5%–97.5% uncertainty interval). The Change column indicates whether there was a greater than 95% posterior probability of increase or decrease in each time period. Groups and time periods correspond to those presented in Figure 2.

Group	Province	District	Treatment	2000 – 2009	Change	2009 – 2013	Change	2013 – 2017	Change
**Western**	Western	Western Rural	Any ORS	1.4 (0 – 2.7)	Increase	2.9 (0.3 – 6)	Increase	-3.2 (-5.5 – -1.1)	Decrease
	Western	Western Rural	Only RHF	-0.2 (-1.5 – 1.2)	Uncertain	-2.3 (-5.3 – 0.2)	Uncertain	3 (0.9 – 5.4)	Increase
	Western	Western Rural	No ORT	-1.2 (-1.8 – -0.6)	Decrease	-0.6 (-1.7 – 0)	Uncertain	0.2 (-0.2 – 0.7)	Uncertain
	Western	Western Urban	Any ORS	1.3 (-0.4 – 2.6)	Uncertain	3.1 (0.3 – 6.4)	Increase	-3.9 (-6.3 – -1.5)	Decrease
	Western	Western Urban	Only RHF	-0.1 (-1.4 – 1.6)	Uncertain	-2.5 (-5.9 – 0.3)	Uncertain	3.7 (1.4 – 6.2)	Increase
	Western	Western Urban	No ORT	-1.2 (-1.9 – -0.5)	Decrease	-0.6 (-1.8 – 0)	Uncertain	0.2 (-0.3 – 0.8)	Uncertain
**Northern**	Northern	Bombali	Any ORS	4.5 (2.9 – 5.8)	Increase	3.2 (0.7 – 6.2)	Increase	-3.2 (-5.4 – -1.3)	Decrease
	Northern	Bombali	Only RHF	-3 (-4.4 – -1.4)	Decrease	-2.4 (-5.3 – 0.1)	Uncertain	3.2 (1.2 – 5.4)	Increase
	Northern	Bombali	No ORT	-1.4 (-2.3 – -0.7)	Decrease	-0.8 (-2.1 – -0.1)	Decrease	0 (-0.3 – 0.4)	Uncertain
	Northern	Kambia	Any ORS	3.6 (2 – 5.2)	Increase	2.8 (0.1 – 6.1)	Increase	-2.2 (-4.8 – -0.1)	Decrease
	Northern	Kambia	Only RHF	-1.8 (-3.5 – 0)	Decrease	-2.2 (-5.5 – 0.4)	Uncertain	2.1 (0 – 4.6)	Uncertain
	Northern	Kambia	No ORT	-1.8 (-2.7 – -1)	Decrease	-0.6 (-1.7 – 0)	Uncertain	0.1 (-0.2 – 0.6)	Uncertain
	Northern	Koinadugu	Any ORS	3.3 (1.6 – 4.8)	Increase	2 (-0.8 – 5.4)	Uncertain	-0.5 (-2.5 – 1.4)	Uncertain
	Northern	Koinadugu	Only RHF	-2.1 (-3.7 – -0.3)	Decrease	-1.3 (-4.3 – 1.4)	Uncertain	0.5 (-1.4 – 2.5)	Uncertain
	Northern	Koinadugu	No ORT	-1.2 (-2.1 – -0.4)	Decrease	-0.8 (-2.1 – 0)	Decrease	0 (-0.3 – 0.4)	Uncertain
	Northern	Port Loko	Any ORS	3 (1.5 – 4.3)	Increase	3 (0.3 – 6.1)	Increase	-2.6 (-4.5 – -0.9)	Decrease
	Northern	Port Loko	Only RHF	-1.6 (-3 – 0)	Decrease	-2.3 (-5.4 – 0.4)	Uncertain	2.5 (0.8 – 4.4)	Increase
	Northern	Port Loko	No ORT	-1.4 (-2 – -0.8)	Decrease	-0.7 (-1.8 – 0)	Decrease	0.1 (-0.2 – 0.6)	Uncertain
	Northern	Tonkolili	Any ORS	3.8 (2.4 – 5.1)	Increase	3 (0.9 – 5.7)	Increase	-2.5 (-4.3 – -0.9)	Decrease
	Northern	Tonkolili	Only RHF	-2.8 (-4.1 – -1.3)	Decrease	-2.2 (-4.8 – 0)	Uncertain	2.6 (0.9 – 4.3)	Increase
	Northern	Tonkolili	No ORT	-1 (-1.7 – -0.4)	Decrease	-0.9 (-2 – -0.1)	Decrease	0 (-0.3 – 0.3)	Uncertain

**Southern and Eastern**	Eastern	Kailahun	Any ORS	0 (-2 – 1.8)	Uncertain	5.7 (2.3 – 9.4)	Increase	-0.3 (-2.4 – 1.7)	Uncertain
	Eastern	Kailahun	Only RHF	0 (-1.8 – 2)	Uncertain	-4.7 (-8.3 – -1.5)	Decrease	0.7 (-1.3 – 2.8)	Uncertain
	Eastern	Kailahun	No ORT	0 (-0.7 – 1)	Uncertain	-1 (-3 – 0.3)	Uncertain	-0.4 (-1.2 – 0.2)	Uncertain
	Eastern	Kenema	Any ORS	0.6 (-1.2 – 2)	Uncertain	5.3 (2.6 – 8.6)	Increase	-0.1 (-1.6 – 1.3)	Uncertain
	Eastern	Kenema	Only RHF	-0.4 (-1.9 – 1.3)	Uncertain	-4 (-7.4 – -1.3)	Decrease	0.2 (-1.2 – 1.7)	Uncertain
	Eastern	Kenema	No ORT	-0.1 (-0.7 – 0.6)	Uncertain	-1.2 (-2.8 – -0.2)	Decrease	-0.1 (-0.4 – 0.1)	Uncertain
	Eastern	Kono	Any ORS	1 (-0.8 – 2.6)	Uncertain	6 (2.9 – 9.6)	Increase	-2.6 (-4.6 – -0.8)	Decrease
	Eastern	Kono	Only RHF	-0.6 (-2.1 – 1.3)	Uncertain	-5.2 (-8.7 – -2.3)	Decrease	2.8 (1 – 4.8)	Increase
	Eastern	Kono	No ORT	-0.5 (-1.1 – 0.3)	Uncertain	-0.8 (-2.3 – 0.1)	Uncertain	-0.2 (-0.6 – 0.2)	Uncertain
	Southern	Bo	Any ORS	1.3 (-0.4 – 2.7)	Uncertain	4.4 (1.9 – 7.4)	Increase	-0.9 (-2.4 – 0.4)	Uncertain
	Southern	Bo	Only RHF	-1 (-2.5 – 0.6)	Uncertain	-3.1 (-6 – -0.7)	Decrease	1 (-0.4 – 2.5)	Uncertain
	Southern	Bo	No ORT	-0.2 (-0.9 – 0.6)	Uncertain	-1.3 (-2.9 – -0.2)	Decrease	-0.1 (-0.4 – 0.2)	Uncertain
	Southern	Bonthe	Any ORS	1 (-1.1 – 2.9)	Uncertain	5.1 (2.1 – 8.7)	Increase	-0.9 (-3 – 1)	Uncertain
	Southern	Bonthe	Only RHF	-0.4 (-2.3 – 1.7)	Uncertain	-4.4 (-8.2 – -1.4)	Decrease	0.9 (-1 – 3)	Uncertain
	Southern	Bonthe	No ORT	-0.7 (-1.4 – 0)	Decrease	-0.6 (-1.6 – -0.1)	Decrease	0 (-0.2 – 0.3)	Uncertain
	Southern	Moyamba	Any ORS	2 (0.4 – 3.5)	Increase	3.3 (0.7 – 6.2)	Increase	-1.6 (-3.8 – 0.1)	Uncertain
	Southern	Moyamba	Only RHF	-1.1 (-2.7 – 0.5)	Uncertain	-2.4 (-5.2 – 0.1)	Uncertain	1.5 (-0.2 – 3.7)	Uncertain
	Southern	Moyamba	No ORT	-0.8 (-1.5 – -0.2)	Decrease	-0.8 (-2 – -0.1)	Decrease	0.1 (-0.2 – 0.5)	Uncertain
	Southern	Pujehun	Any ORS	0.1 (-2 – 2.2)	Uncertain	6 (2.5 – 9.9)	Increase	-0.3 (-2.1 – 1.4)	Uncertain
	Southern	Pujehun	Only RHF	0.1 (-2 – 2.3)	Uncertain	-5.1 (-9 – -1.6)	Decrease	0.3 (-1.4 – 2)	Uncertain
	Southern	Pujehun	No ORT	-0.2 (-0.9 – 0.6)	Uncertain	-0.9 (-2.4 – -0.1)	Decrease	0 (-0.4 – 0.4)	Uncertain

b) Mali

Average yearly change in treatment coverage by Region, Cercle, Treatment (Any ORS, Only RHF, and No ORT) for three time periods. Estimates are shown as percentage change per year in the format: mean (2.5%–97.5% uncertainty interval). The Change column indicates whether there was a greater than 95% posterior probability of increase or decrease in each time period. Groups and time periods correspond to those presented in Figure 3.

Group	Region	Cercle	Treatment	2001 – 2004	Change	2004 – 2011	Change	2011 – 2018	Change
Bamako	Bamako	Bamako	Any ORS	-2.3 (-9.4 – 7.4)	Uncertain	0.4 (-4.7 – 5.1)	Uncertain	-0.3 (-4.2 – 2.7)	Uncertain
	Bamako	Bamako	Only RHF	9 (-2.7 – 17.7)	Uncertain	-0.5 (-6 – 5.6)	Uncertain	1.7 (-1.8 – 6.2)	Uncertain
	Bamako	Bamako	No ORT	-6.7 (-10.7 – 0.1)	Uncertain	0.1 (-3.1 – 3.5)	Uncertain	-1.4 (-4.2 – 0)	Decrease
Northern	Gao	Ansongo	Any ORS	2 (-2.6 – 9.7)	Uncertain	0.3 (-3.3 – 4)	Uncertain	2.4 (-1.3 – 5.4)	Uncertain
	Gao	Ansongo	Only RHF	-4.1 (-13.2 – 2.1)	Uncertain	-0.4 (-5.1 – 4.5)	Uncertain	-1.9 (-5.8 – 2.4)	Uncertain
	Gao	Ansongo	No ORT	2.1 (-0.9 – 7.9)	Uncertain	0.1 (-2.9 – 2.8)	Uncertain	-0.4 (-3.2 – 1.6)	Uncertain
	Gao	Bourem	Any ORS	1 (-3.2 – 7.5)	Uncertain	0.2 (-3 – 3.3)	Uncertain	3 (0 – 5.3)	Increase
	Gao	Bourem	Only RHF	-2.5 (-11.2 – 3.1)	Uncertain	-0.4 (-4.6 – 3.8)	Uncertain	-2.2 (-5.1 – 1.6)	Uncertain
	Gao	Bourem	No ORT	1.5 (-1.5 – 6.3)	Uncertain	0.2 (-2.2 – 2.7)	Uncertain	-0.8 (-3 – 0.5)	Uncertain
	Gao	Gao	Any ORS	0.6 (-5.1 – 8.8)	Uncertain	-0.2 (-3.9 – 3.6)	Uncertain	2.9 (-0.4 – 5.4)	Uncertain
	Gao	Gao	Only RHF	-2.4 (-12.9 – 4.8)	Uncertain	0.4 (-4.3 – 5.2)	Uncertain	-2.6 (-5.6 – 1.3)	Uncertain
	Gao	Gao	No ORT	1.8 (-1.6 – 8.4)	Uncertain	-0.2 (-3.2 – 2.3)	Uncertain	-0.3 (-2.5 – 1.1)	Uncertain
	Gao	Ménaka	Any ORS	0.4 (-4.5 – 5.6)	Uncertain	0.6 (-2.1 – 3.5)	Uncertain	0.3 (-2.8 – 3.4)	Uncertain
	Gao	Ménaka	Only RHF	0.3 (-7.5 – 7.1)	Uncertain	-0.6 (-4.2 – 3.2)	Uncertain	0.2 (-3.3 – 4.1)	Uncertain
	Gao	Ménaka	No ORT	-0.8 (-4.9 – 4.1)	Uncertain	0 (-2.2 – 2.2)	Uncertain	-0.5 (-2.5 – 1.3)	Uncertain
	Kidal	Abeïbara	Any ORS	0.9 (-3.9 – 6.7)	Uncertain	-0.3 (-3 – 2.2)	Uncertain	0.6 (-1.9 – 3.3)	Uncertain
	Kidal	Abeïbara	Only RHF	-1.8 (-9.8 – 5)	Uncertain	0.5 (-3.3 – 4.3)	Uncertain	-0.4 (-3.8 – 2.9)	Uncertain
	Kidal	Abeïbara	No ORT	0.9 (-3.2 – 6.3)	Uncertain	-0.2 (-2.5 – 1.9)	Uncertain	-0.2 (-2.3 – 1.5)	Uncertain
	Kidal	Kidal	Any ORS	1.1 (-3.6 – 7.7)	Uncertain	0 (-3 – 3)	Uncertain	0.5 (-2.5 – 2.6)	Uncertain
	Kidal	Kidal	Only RHF	-2.4 (-11.2 – 4.2)	Uncertain	0 (-4.3 – 4.4)	Uncertain	-0.3 (-3.2 – 3.5)	Uncertain
	Kidal	Kidal	No ORT	1.2 (-1.9 – 6.7)	Uncertain	0 (-2.4 – 2.6)	Uncertain	-0.2 (-2.4 – 1.3)	Uncertain
	Kidal	Tessalit	Any ORS	1 (-3.2 – 6.4)	Uncertain	-0.1 (-2.5 – 2.6)	Uncertain	0.7 (-1.9 – 3.3)	Uncertain
	Kidal	Tessalit	Only RHF	-2 (-9.7 – 3.6)	Uncertain	0.2 (-3.1 – 3.5)	Uncertain	-0.4 (-3.8 – 2.9)	Uncertain
	Kidal	Tessalit	No ORT	1 (-2.3 – 5.6)	Uncertain	-0.1 (-2.2 – 1.8)	Uncertain	-0.2 (-2.1 – 1.4)	Uncertain

	Kidal	Tin-Essako	Any ORS	0.9 (-4.1 – 7.3)	Uncertain	-0.1 (-3 – 2.9)	Uncertain	0.4 (-2.7 – 3.1)	Uncertain
	Kidal	Tin-Essako	Only RHF	-1.7 (-10.4 – 5.1)	Uncertain	0.2 (-3.6 – 4.3)	Uncertain	-0.2 (-3.6 – 3.3)	Uncertain
	Kidal	Tin-Essako	No ORT	0.8 (-3.3 – 6)	Uncertain	-0.1 (-2.6 – 2.2)	Uncertain	-0.2 (-2.4 – 1.5)	Uncertain
	Mopti	Bandiagara	Any ORS	3 (-1.7 – 10.5)	Uncertain	0.3 (-3.6 – 4.1)	Uncertain	2.1 (-1.1 – 4.6)	Uncertain
	Mopti	Bandiagara	Only RHF	-4.3 (-14.6 – 2.4)	Uncertain	0 (-5 – 4.9)	Uncertain	-1.7 (-4.7 – 2.3)	Uncertain
	Mopti	Bandiagara	No ORT	1.3 (-2.2 – 7.3)	Uncertain	-0.3 (-3.3 – 2.5)	Uncertain	-0.5 (-2.8 – 0.8)	Uncertain
	Mopti	Bankass	Any ORS	2.9 (-1.5 – 10.3)	Uncertain	0.2 (-3.6 – 4.2)	Uncertain	1.3 (-2.3 – 3.7)	Uncertain
	Mopti	Bankass	Only RHF	-2.5 (-12.5 – 4.2)	Uncertain	0.2 (-5 – 5.2)	Uncertain	-0.8 (-3.7 – 3.8)	Uncertain
	Mopti	Bankass	No ORT	-0.4 (-4.4 – 6.4)	Uncertain	-0.4 (-3.8 – 2.3)	Uncertain	-0.6 (-2.8 – 0.6)	Uncertain
	Mopti	Djenné	Any ORS	2.9 (-1.4 – 10.8)	Uncertain	0.1 (-3.3 – 3.9)	Uncertain	0.5 (-2.8 – 3.1)	Uncertain
	Mopti	Djenné	Only RHF	-4.7 (-14.8 – 1.9)	Uncertain	-0.5 (-5.7 – 4.7)	Uncertain	0.9 (-2.4 – 5.3)	Uncertain
	Mopti	Djenné	No ORT	1.8 (-1.7 – 9.2)	Uncertain	0.3 (-3.1 – 3.6)	Uncertain	-1.4 (-4.5 – 0.2)	Uncertain
	Mopti	Douentza	Any ORS	2.8 (-0.8 – 9)	Uncertain	0.6 (-2.4 – 3.7)	Uncertain	1.4 (-1.9 – 4.4)	Uncertain
	Mopti	Douentza	Only RHF	-4.6 (-13 – 0.8)	Uncertain	-0.2 (-4.3 – 3.7)	Uncertain	-1.1 (-4.5 – 2.8)	Uncertain
	Mopti	Douentza	No ORT	1.8 (-1 – 7.3)	Uncertain	-0.4 (-2.9 – 1.7)	Uncertain	-0.3 (-2.1 – 1)	Uncertain
	Mopti	Koro	Any ORS	2.9 (-1.4 – 11)	Uncertain	0.3 (-3.2 – 3.9)	Uncertain	2.2 (-1.4 – 5)	Uncertain
	Mopti	Koro	Only RHF	-3.8 (-13.2 – 2.7)	Uncertain	0.3 (-4.3 – 5)	Uncertain	-2.1 (-5.2 – 1.8)	Uncertain
	Mopti	Koro	No ORT	0.9 (-2.6 – 6.9)	Uncertain	-0.6 (-3.2 – 1.5)	Uncertain	-0.1 (-1.8 – 1.2)	Uncertain
	Mopti	Mopti	Any ORS	3.4 (-0.8 – 10.9)	Uncertain	0.5 (-3.4 – 4.8)	Uncertain	2.2 (-1.4 – 5)	Uncertain
	Mopti	Mopti	Only RHF	-4.8 (-14.4 – 1.1)	Uncertain	-1 (-6.1 – 3.8)	Uncertain	-1 (-4.4 – 3.2)	Uncertain
	Mopti	Mopti	No ORT	1.4 (-1.8 – 7.6)	Uncertain	0.5 (-2.7 – 3.7)	Uncertain	-1.2 (-4.3 – 0.6)	Uncertain
	Mopti	Ténenkou	Any ORS	3.3 (-0.8 – 10.2)	Uncertain	0.7 (-2.8 – 4.5)	Uncertain	1.4 (-2.4 – 4.8)	Uncertain
	Mopti	Ténenkou	Only RHF	-5 (-14.7 – 0.7)	Uncertain	-1 (-5.9 – 3.6)	Uncertain	-0.4 (-4.4 – 4.3)	Uncertain
	Mopti	Ténenkou	No ORT	1.7 (-1.4 – 8.2)	Uncertain	0.3 (-2.9 – 3.4)	Uncertain	-1 (-4 – 0.8)	Uncertain
	Mopti	Youwarou	Any ORS	2.9 (-0.6 – 9.5)	Uncertain	1.1 (-2.7 – 5.3)	Uncertain	1.1 (-2.8 – 4)	Uncertain
	Mopti	Youwarou	Only RHF	-4.4 (-13.1 – 0.8)	Uncertain	-1.4 (-6.2 – 3.5)	Uncertain	-0.1 (-3.7 – 4.6)	Uncertain
	Mopti	Youwarou	No ORT	1.5 (-1.2 – 7.7)	Uncertain	0.2 (-2.8 – 3.2)	Uncertain	-1 (-3.7 – 0.4)	Uncertain
	Timbuktu	Diré	Any ORS	3.4 (-0.7 – 11.4)	Uncertain	0.8 (-3.4 – 4.8)	Uncertain	0.3 (-3.3 – 2.9)	Uncertain
	Timbuktu	Diré	Only RHF	-5.4 (-16 – 0.6)	Uncertain	-0.5 (-5.4 – 4.5)	Uncertain	0.2 (-2.8 – 4.6)	Uncertain

	Timbuktu	Diré	No ORT	2 (-1 – 8.1)	Uncertain	-0.3 (-3.3 – 2.2)	Uncertain	-0.6 (-2.9 – 0.5)	Uncertain
	Timbuktu	Goundam	Any ORS	3 (-0.5 – 9.6)	Uncertain	0.7 (-2.5 – 4)	Uncertain	0.3 (-2.9 – 2.4)	Uncertain
	Timbuktu	Goundam	Only RHF	-5 (-14.1 – 0.1)	Uncertain	-0.4 (-4.7 – 3.8)	Uncertain	0.2 (-2.3 – 4)	Uncertain
	Timbuktu	Goundam	No ORT	1.9 (-0.8 – 6.9)	Uncertain	-0.3 (-2.7 – 1.9)	Uncertain	-0.6 (-2.5 – 0.4)	Uncertain
	Timbuktu	Gourma-Rharous	Any ORS	2.5 (-1.1 – 8.4)	Uncertain	0.2 (-2.9 – 2.9)	Uncertain	2.1 (-0.9 – 4.6)	Uncertain
	Timbuktu	Gourma-Rharous	Only RHF	-4.3 (-11.9 – 0.6)	Uncertain	0.1 (-3.5 – 3.9)	Uncertain	-1.6 (-4.6 – 1.8)	Uncertain
	Timbuktu	Gourma-Rharous	No ORT	1.9 (-0.9 – 6.8)	Uncertain	-0.3 (-2.5 – 1.9)	Uncertain	-0.5 (-2.3 – 0.7)	Uncertain
	Timbuktu	Niafunké	Any ORS	3 (-0.4 – 9.5)	Uncertain	1 (-2.4 – 4.6)	Uncertain	0.6 (-3 – 2.9)	Uncertain
	Timbuktu	Niafunké	Only RHF	-4.8 (-13.4 – 0.4)	Uncertain	-0.9 (-5.4 – 3.5)	Uncertain	0.3 (-2.6 – 4.2)	Uncertain
	Timbuktu	Niafunké	No ORT	1.8 (-1.1 – 7.5)	Uncertain	-0.1 (-2.9 – 2.4)	Uncertain	-0.8 (-3 – 0.3)	Uncertain
	Timbuktu	Tombouctou	Any ORS	3 (-0.7 – 9.9)	Uncertain	0 (-3.3 – 3.3)	Uncertain	1.2 (-1.7 – 3.3)	Uncertain
	Timbuktu	Tombouctou	Only RHF	-5 (-13.9 – 0.5)	Uncertain	0.4 (-3.6 – 4.7)	Uncertain	-1 (-3.4 – 2.4)	Uncertain
	Timbuktu	Tombouctou	No ORT	2 (-0.7 – 7.4)	Uncertain	-0.4 (-3.1 – 1.5)	Uncertain	-0.3 (-1.8 – 0.7)	Uncertain
**Southern**	Kayes	Bafoulabé	Any ORS	1.7 (-2.8 – 8.5)	Uncertain	0.4 (-3.2 – 4.1)	Uncertain	-0.3 (-3.4 – 2.1)	Uncertain
	Kayes	Bafoulabé	Only RHF	9.9 (1.2 – 16.4)	Increase	-0.2 (-4.5 – 4.3)	Uncertain	1.4 (-1.5 – 5.2)	Uncertain
	Kayes	Bafoulabé	No ORT	-11.6 (-16 – -5.6)	Decrease	-0.2 (-2.9 – 2.4)	Uncertain	-1.1 (-3.3 – 0)	Uncertain
	Kayes	Diéma	Any ORS	2.8 (-1.5 – 9.7)	Uncertain	0.2 (-3.3 – 4)	Uncertain	-1.4 (-4.5 – 0.6)	Uncertain
	Kayes	Diéma	Only RHF	2.9 (-6 – 9.6)	Uncertain	0.1 (-4.3 – 4.6)	Uncertain	2.2 (-0.2 – 6)	Uncertain
	Kayes	Diéma	No ORT	-5.8 (-9.9 – 0.5)	Uncertain	-0.4 (-3 – 2.3)	Uncertain	-0.8 (-2.8 – 0.1)	Uncertain
	Kayes	Kayes	Any ORS	-2.3 (-7.9 – 5.4)	Uncertain	0.3 (-3.8 – 4)	Uncertain	-0.3 (-3.7 – 2.2)	Uncertain
	Kayes	Kayes	Only RHF	8.5 (-1.6 – 16)	Uncertain	-0.1 (-4.6 – 4.8)	Uncertain	1.1 (-1.9 – 5.1)	Uncertain
	Kayes	Kayes	No ORT	-6.2 (-10.6 – -0.2)	Decrease	-0.2 (-2.7 – 2)	Uncertain	-0.7 (-2.7 – 0.3)	Uncertain
	Kayes	Kita	Any ORS	3.6 (-0.8 – 10.7)	Uncertain	0.4 (-3.3 – 3.6)	Uncertain	-0.3 (-3.3 – 2)	Uncertain
	Kayes	Kita	Only RHF	3.5 (-5.5 – 10.1)	Uncertain	-0.1 (-4.3 – 4.6)	Uncertain	1.3 (-1.3 – 4.9)	Uncertain
	Kayes	Kita	No ORT	-7.1 (-11.2 – -1.1)	Decrease	-0.2 (-2.9 – 2.2)	Uncertain	-1.1 (-3.1 – 0)	Decrease
	Kayes	Kéniéba	Any ORS	1.8 (-3.2 – 10)	Uncertain	0.4 (-3.4 – 4.2)	Uncertain	0.1 (-3.3 – 2.6)	Uncertain
	Kayes	Kéniéba	Only RHF	3.7 (-6 – 11)	Uncertain	-0.5 (-5.4 – 4.5)	Uncertain	1.4 (-1.9 – 5.5)	Uncertain
	Kayes	Kéniéba	No ORT	-5.6 (-10.7 – 1.2)	Uncertain	0.1 (-2.8 – 3.2)	Uncertain	-1.5 (-4.1 – 0)	Uncertain
	Kayes	Nioro	Any ORS	1.6 (-3.1 – 9)	Uncertain	0.6 (-2.7 – 4.3)	Uncertain	-1.9 (-5.1 – 0.1)	Uncertain

	Kayes	Nioro	Only RHF	0.1 (-8.7 – 6.6)	Uncertain	-0.3 (-5.3 – 4)	Uncertain	2.7 (0.1 – 6.8)	Increase
	Kayes	Nioro	No ORT	-1.7 (-5.7 – 3.9)	Uncertain	-0.3 (-2.8 – 2.4)	Uncertain	-0.8 (-2.9 – 0.2)	Uncertain
	Kayes	Yélimané	Any ORS	-1.1 (-6.4 – 7.1)	Uncertain	0.5 (-3.3 – 4.5)	Uncertain	-1.5 (-4.9 – 0.8)	Uncertain
	Kayes	Yélimané	Only RHF	2.2 (-7.5 – 9)	Uncertain	-0.3 (-5 – 4.4)	Uncertain	2.2 (-0.6 – 6.4)	Uncertain
	Kayes	Yélimané	No ORT	-1 (-5.1 – 4.6)	Uncertain	-0.3 (-3 – 2.2)	Uncertain	-0.7 (-2.9 – 0.2)	Uncertain
	Koulikoro	Banamba	Any ORS	2.2 (-2.5 – 10.1)	Uncertain	0.2 (-3.7 – 4)	Uncertain	0.2 (-3.4 – 2.9)	Uncertain
	Koulikoro	Banamba	Only RHF	8.5 (-1.7 – 15.7)	Uncertain	0.2 (-4.5 – 4.8)	Uncertain	0.4 (-3 – 4.5)	Uncertain
	Koulikoro	Banamba	No ORT	-10.6 (-15.7 – -3.4)	Decrease	-0.3 (-3.6 – 2.2)	Uncertain	-0.6 (-2.9 – 0.8)	Uncertain
	Koulikoro	Dioïla	Any ORS	-0.5 (-5.4 – 7)	Uncertain	0.5 (-3.2 – 4.3)	Uncertain	-0.2 (-3.7 – 2.3)	Uncertain
	Koulikoro	Dioïla	Only RHF	4.7 (-5.6 – 11.5)	Uncertain	-0.3 (-5 – 4.8)	Uncertain	0.9 (-2.1 – 4.8)	Uncertain
	Koulikoro	Dioïla	No ORT	-4.2 (-8.6 – 2.1)	Uncertain	-0.2 (-3 – 2.5)	Uncertain	-0.7 (-3 – 0.5)	Uncertain
	Koulikoro	Kangaba	Any ORS	3.2 (-2.1 – 11.5)	Uncertain	0.8 (-3.5 – 4.7)	Uncertain	0 (-4.1 – 3.4)	Uncertain
	Koulikoro	Kangaba	Only RHF	-3 (-13.6 – 4.5)	Uncertain	-1 (-5.8 – 4.4)	Uncertain	1.1 (-3.1 – 5.6)	Uncertain
	Koulikoro	Kangaba	No ORT	-0.2 (-4.3 – 6.6)	Uncertain	0.2 (-3.1 – 3.2)	Uncertain	-1.1 (-3.8 – 0.6)	Uncertain
	Koulikoro	Kati	Any ORS	-0.5 (-6.2 – 8.3)	Uncertain	0.4 (-3.8 – 4.3)	Uncertain	-0.1 (-3.6 – 2.4)	Uncertain
	Koulikoro	Kati	Only RHF	6.1 (-5 – 13.6)	Uncertain	-0.5 (-5.1 – 4.7)	Uncertain	1.4 (-1.7 – 5.5)	Uncertain
	Koulikoro	Kati	No ORT	-5.6 (-9.3 – 0.9)	Uncertain	0.1 (-2.9 – 3.2)	Uncertain	-1.4 (-3.9 – -0.1)	Decrease
	Koulikoro	Kolokani	Any ORS	1.5 (-3.7 – 9)	Uncertain	0 (-3.7 – 3.7)	Uncertain	-0.2 (-3.7 – 2.3)	Uncertain
	Koulikoro	Kolokani	Only RHF	4 (-5.2 – 11.1)	Uncertain	0.1 (-4.6 – 4.8)	Uncertain	1.3 (-1.7 – 5.3)	Uncertain
	Koulikoro	Kolokani	No ORT	-5.4 (-9.8 – 1.2)	Uncertain	-0.1 (-3 – 2.6)	Uncertain	-1.1 (-3.5 – 0.1)	Uncertain
	Koulikoro	Koulikoro	Any ORS	0.4 (-4.7 – 8.5)	Uncertain	0.2 (-3.7 – 4)	Uncertain	0.1 (-3.6 – 2.5)	Uncertain
	Koulikoro	Koulikoro	Only RHF	10.7 (1.2 – 17.9)	Increase	0.1 (-4.8 – 4.8)	Uncertain	0.5 (-2.5 – 4.4)	Uncertain
	Koulikoro	Koulikoro	No ORT	-11.1 (-15.7 – -4.2)	Decrease	-0.4 (-3.2 – 1.9)	Uncertain	-0.6 (-2.6 – 0.5)	Uncertain
	Koulikoro	Nara	Any ORS	2.2 (-1.7 – 8.5)	Uncertain	0.1 (-3 – 3.1)	Uncertain	-0.5 (-3 – 1.7)	Uncertain
	Koulikoro	Nara	Only RHF	-1.3 (-9.3 – 4.5)	Uncertain	-0.1 (-3.8 – 3.7)	Uncertain	1.2 (-1.6 – 4.4)	Uncertain
	Koulikoro	Nara	No ORT	-0.9 (-4.8 – 4.6)	Uncertain	0 (-2.3 – 2.5)	Uncertain	-0.7 (-2.9 – 0.8)	Uncertain
	Sikasso	Bougouni	Any ORS	1 (-3.6 – 8.4)	Uncertain	0.7 (-3 – 4.2)	Uncertain	0.4 (-3 – 3.2)	Uncertain
	Sikasso	Bougouni	Only RHF	0.5 (-8.5 – 6.8)	Uncertain	-1.3 (-5.6 – 3.5)	Uncertain	1.3 (-2.1 – 5.1)	Uncertain
	Sikasso	Bougouni	No ORT	-1.5 (-5.1 – 4.2)	Uncertain	0.6 (-2.2 – 3.5)	Uncertain	-1.7 (-4.4 – -0.1)	Decrease

	Sikasso	Kadiolo	Any ORS	-3.2 (-8.9 – 6.2)	Uncertain	-0.1 (-4.3 – 3.7)	Uncertain	0.9 (-2.6 – 3.9)	Uncertain
	Sikasso	Kadiolo	Only RHF	4.2 (-6.5 – 12)	Uncertain	-0.2 (-5.3 – 5.2)	Uncertain	0.5 (-3.3 – 5)	Uncertain
	Sikasso	Kadiolo	No ORT	-1 (-4.9 – 6.1)	Uncertain	0.3 (-3.2 – 3.8)	Uncertain	-1.4 (-4.4 – 0.3)	Uncertain
	Sikasso	Kolondiéba	Any ORS	-1.1 (-6.4 – 6.7)	Uncertain	0.7 (-3.3 – 4.4)	Uncertain	1 (-3 – 4)	Uncertain
	Sikasso	Kolondiéba	Only RHF	2.5 (-7 – 9.3)	Uncertain	-1.3 (-6.1 – 3.8)	Uncertain	0.6 (-3.2 – 5.1)	Uncertain
	Sikasso	Kolondiéba	No ORT	-1.4 (-5.3 – 5)	Uncertain	0.6 (-2.7 – 3.8)	Uncertain	-1.6 (-4.5 – 0.1)	Uncertain
	Sikasso	Koutiala	Any ORS	-0.3 (-5.6 – 8.2)	Uncertain	0.3 (-3.8 – 4.4)	Uncertain	1.3 (-2.4 – 4.1)	Uncertain
	Sikasso	Koutiala	Only RHF	0.9 (-9.2 – 8.3)	Uncertain	-0.7 (-6.1 – 4.5)	Uncertain	-0.7 (-4.4 – 3.7)	Uncertain
	Sikasso	Koutiala	No ORT	-0.6 (-4.6 – 5.6)	Uncertain	0.4 (-2.7 – 3.6)	Uncertain	-0.6 (-3.5 – 1.2)	Uncertain
	Sikasso	Sikasso	Any ORS	0.2 (-5.1 – 8.6)	Uncertain	0.4 (-3.8 – 4.3)	Uncertain	0.1 (-3.6 – 2.9)	Uncertain
	Sikasso	Sikasso	Only RHF	0.1 (-9.8 – 7.3)	Uncertain	-1.4 (-6.3 – 3.9)	Uncertain	2 (-1.7 – 6.3)	Uncertain
	Sikasso	Sikasso	No ORT	-0.3 (-3.9 – 6.4)	Uncertain	1 (-2.6 – 4.8)	Uncertain	-2.1 (-5.4 – -0.2)	Decrease
	Sikasso	Yanfolila	Any ORS	3.2 (-1.5 – 10.4)	Uncertain	1.1 (-2.9 – 4.9)	Uncertain	-0.1 (-4 – 3.3)	Uncertain
	Sikasso	Yanfolila	Only RHF	-4.4 (-14.6 – 2.4)	Uncertain	-1.6 (-6.4 – 3.7)	Uncertain	1.6 (-2.4 – 6.4)	Uncertain
	Sikasso	Yanfolila	No ORT	1.2 (-2.3 – 7.6)	Uncertain	0.5 (-2.5 – 3.8)	Uncertain	-1.5 (-4.7 – 0.3)	Uncertain
	Sikasso	Yorosso	Any ORS	1.1 (-4.2 – 9.9)	Uncertain	0.4 (-4.1 – 4.8)	Uncertain	1 (-3.1 – 4)	Uncertain
	Sikasso	Yorosso	Only RHF	-2.5 (-13 – 4.6)	Uncertain	-1.3 (-6.6 – 4.5)	Uncertain	1 (-3.1 – 5.8)	Uncertain
	Sikasso	Yorosso	No ORT	1.4 (-2.4 – 8.4)	Uncertain	0.9 (-2.7 – 4.5)	Uncertain	-2 (-5.4 – 0)	Uncertain
	Ségou	Barouéli	Any ORS	-0.3 (-5.1 – 7.2)	Uncertain	0.8 (-3 – 4.9)	Uncertain	-0.2 (-3.9 – 2.5)	Uncertain
	Ségou	Barouéli	Only RHF	6.4 (-4 – 13.3)	Uncertain	-0.4 (-5.3 – 4.7)	Uncertain	-0.7 (-3.9 – 3.6)	Uncertain
	Ségou	Barouéli	No ORT	-6.1 (-10.7 – 0.1)	Uncertain	-0.4 (-3.5 – 2.2)	Uncertain	0.8 (-1.2 – 2.4)	Uncertain
	Ségou	Bla	Any ORS	-1.9 (-7.2 – 6.1)	Uncertain	0.2 (-3.7 – 3.9)	Uncertain	1.2 (-2.2 – 3.5)	Uncertain
	Ségou	Bla	Only RHF	2.6 (-6.9 – 10.2)	Uncertain	-0.1 (-5 – 4.8)	Uncertain	-2.6 (-5.5 – 1.7)	Uncertain
	Ségou	Bla	No ORT	-0.7 (-4.9 – 5.3)	Uncertain	-0.1 (-3 – 2.6)	Uncertain	1.4 (-1.1 – 3.2)	Uncertain
	Ségou	Macina	Any ORS	1.5 (-3.5 – 9.5)	Uncertain	0.4 (-3.6 – 4.4)	Uncertain	0.5 (-3.1 – 3.1)	Uncertain
	Ségou	Macina	Only RHF	-2.2 (-12.7 – 5.2)	Uncertain	-0.4 (-5.6 – 4.7)	Uncertain	0.1 (-3.2 – 4)	Uncertain
	Ségou	Macina	No ORT	0.7 (-3.4 – 7.1)	Uncertain	0.1 (-3.1 – 3.1)	Uncertain	-0.6 (-3.3 – 1)	Uncertain
	Ségou	Niono	Any ORS	2 (-3 – 10.7)	Uncertain	0.8 (-3.5 – 5)	Uncertain	1.9 (-2.2 – 5)	Uncertain
	Ségou	Niono	Only RHF	-1 (-11.9 – 5.8)	Uncertain	-0.6 (-5.5 – 4.9)	Uncertain	-2.2 (-5.9 – 2.2)	Uncertain

	Ségou	Niono	No ORT	-1 (-5.1 – 5.9)	Uncertain	-0.3 (-3.2 – 2.3)	Uncertain	0.3 (-2.1 – 2.1)	Uncertain
	Ségou	San	Any ORS	0.8 (-4.3 – 8.6)	Uncertain	0 (-3.7 – 3.8)	Uncertain	0 (-3.2 – 2.3)	Uncertain
	Ségou	San	Only RHF	-2.3 (-11.5 – 4.6)	Uncertain	-0.1 (-5.3 – 4.5)	Uncertain	1.1 (-1.9 – 5.3)	Uncertain
	Ségou	San	No ORT	1.5 (-1.9 – 7.9)	Uncertain	0.1 (-3 – 3.3)	Uncertain	-1.1 (-3.7 – 0.3)	Uncertain
	Ségou	Ségou	Any ORS	-0.4 (-5.7 – 7.4)	Uncertain	0.6 (-3.3 – 4.7)	Uncertain	0.2 (-3.5 – 2.7)	Uncertain
	Ségou	Ségou	Only RHF	1.2 (-8.5 – 8.8)	Uncertain	-0.5 (-5.5 – 4.5)	Uncertain	-2.1 (-5.1 – 2.4)	Uncertain
	Ségou	Ségou	No ORT	-0.8 (-5.1 – 6.6)	Uncertain	-0.2 (-3.4 – 2.7)	Uncertain	1.9 (-0.6 – 3.5)	Uncertain
	Ségou	Tominian	Any ORS	2.2 (-1.9 – 9.3)	Uncertain	0.2 (-3.5 – 3.7)	Uncertain	-0.1 (-3.2 – 2.1)	Uncertain
	Ségou	Tominian	Only RHF	-4.2 (-12.9 – 2.1)	Uncertain	-0.3 (-5 – 4.4)	Uncertain	1.4 (-1.6 – 5.5)	Uncertain
	Ségou	Tominian	No ORT	2 (-1.3 – 8.3)	Uncertain	0.2 (-3 – 3.1)	Uncertain	-1.3 (-3.9 – 0.1)	Uncertain

c) Senegal

Average yearly change in treatment coverage by Region, Department, Treatment (Any ORS, Only RHF, and No ORT) for three time periods. Estimates are shown as percentage change per year in the format: mean (2.5%–97.5% uncertainty interval). The Change column indicates whether there was a greater than 95% posterior probability of increase or decrease in each time period. Groups and time periods correspond to those presented in Figure 4.

Group	Region	Department	Treatment	2000 – 2006	Change	2006 – 2012	Change	2012 – 2017	Change
**Northern**	Louga	Kébémer	Any ORS	1.9 (0.7 – 3.1)	Increase	0.3 (-1.1 – 1.8)	Uncertain	0.1 (-1.6 – 1.7)	Uncertain
	Louga	Kébémer	Only RHF	2.1 (0.4 – 3.6)	Increase	0.1 (-1.6 – 1.7)	Uncertain	0.2 (-1.6 – 2)	Uncertain
	Louga	Kébémer	No ORT	-3.9 (-5 – -2.8)	Decrease	-0.4 (-1.1 – 0.2)	Uncertain	-0.3 (-0.8 – 0.2)	Uncertain
	Louga	Linguère	Any ORS	1.4 (0.3 – 2.6)	Increase	0.2 (-1.1 – 1.3)	Uncertain	0.8 (-0.8 – 2.2)	Uncertain
	Louga	Linguère	Only RHF	2.1 (0.7 – 3.7)	Increase	0.2 (-1.1 – 1.6)	Uncertain	-0.6 (-2.2 – 1.1)	Uncertain
	Louga	Linguère	No ORT	-3.5 (-4.6 – -2.5)	Decrease	-0.4 (-1 – 0.1)	Uncertain	-0.2 (-0.6 – 0.1)	Uncertain
	Louga	Louga	Any ORS	1.8 (0.5 – 3)	Increase	0.4 (-1.1 – 1.9)	Uncertain	0.3 (-1.4 – 2)	Uncertain
	Louga	Louga	Only RHF	1.6 (-0.1 – 3.2)	Uncertain	0.1 (-1.6 – 1.7)	Uncertain	0 (-1.9 – 1.9)	Uncertain
	Louga	Louga	No ORT	-3.4 (-4.5 – -2.3)	Decrease	-0.4 (-1.1 – 0.2)	Uncertain	-0.3 (-0.8 – 0.1)	Uncertain
	Matam	Kanel	Any ORS	1.7 (0.4 – 3)	Increase	0 (-1.5 – 1.4)	Uncertain	1.1 (-0.7 – 2.7)	Uncertain
	Matam	Kanel	Only RHF	2.4 (0.8 – 4.2)	Increase	0.6 (-1 – 2.2)	Uncertain	-0.9 (-2.6 – 1.1)	Uncertain
	Matam	Kanel	No ORT	-4.2 (-5.5 – -2.9)	Decrease	-0.5 (-1.2 – 0)	Uncertain	-0.2 (-0.7 – 0.1)	Uncertain
	Matam	Matam	Any ORS	1.9 (0.6 – 3.3)	Increase	-0.2 (-1.6 – 1.4)	Uncertain	1.2 (-0.6 – 3)	Uncertain
	Matam	Matam	Only RHF	2 (0.2 – 3.8)	Increase	0.6 (-1.2 – 2.2)	Uncertain	-1 (-2.9 – 0.9)	Uncertain
	Matam	Matam	No ORT	-3.9 (-5.1 – -2.6)	Decrease	-0.4 (-1 – 0.1)	Uncertain	-0.2 (-0.6 – 0.1)	Uncertain
	Matam	Ranérou Ferlo	Any ORS	1.3 (0.1 – 2.4)	Increase	0.2 (-1 – 1.4)	Uncertain	0.9 (-0.6 – 2.5)	Uncertain
	Matam	Ranérou Ferlo	Only RHF	2.8 (1.3 – 4.5)	Increase	0.2 (-1.2 – 1.6)	Uncertain	-0.7 (-2.3 – 0.9)	Uncertain
	Matam	Ranérou Ferlo	No ORT	-4.1 (-5.4 – -3)	Decrease	-0.4 (-1 – 0.1)	Uncertain	-0.2 (-0.6 – 0.1)	Uncertain
	Saint-Louis	Dagana	Any ORS	1.8 (0.6 – 3)	Increase	0.5 (-1.1 – 1.9)	Uncertain	0.6 (-1.3 – 2.4)	Uncertain
	Saint-Louis	Dagana	Only RHF	1.9 (0.2 – 3.4)	Increase	-0.1 (-1.7 – 1.6)	Uncertain	-0.3 (-2.3 – 1.7)	Uncertain
	Saint-Louis	Dagana	No ORT	-3.7 (-4.8 – -2.5)	Decrease	-0.4 (-1 – 0.2)	Uncertain	-0.3 (-0.8 – 0.1)	Uncertain
	Saint-Louis	Podor	Any ORS	1.6 (0.6 – 2.7)	Increase	0.2 (-1 – 1.5)	Uncertain	0.9 (-0.7 – 2.5)	Uncertain
	Saint-Louis	Podor	Only RHF	2.1 (0.5 – 3.7)	Increase	0.1 (-1.3 – 1.5)	Uncertain	-0.7 (-2.3 – 1.1)	Uncertain

	Saint-Louis	Podor	No ORT	-3.7 (-4.9 – -2.7)	Decrease	-0.4 (-0.9 – 0.1)	Uncertain	-0.2 (-0.6 – 0.1)	Uncertain
	Saint-Louis	Saint-Louis	Any ORS	1.8 (0.2 – 3.5)	Increase	0.3 (-1.6 – 2.3)	Uncertain	0.5 (-1.9 – 2.9)	Uncertain
	Saint-Louis	Saint-Louis	Only RHF	1.7 (-0.6 – 3.9)	Uncertain	0 (-2.3 – 2.1)	Uncertain	-0.1 (-2.6 – 2.4)	Uncertain
	Saint-Louis	Saint-Louis	No ORT	-3.5 (-5 – -2)	Decrease	-0.3 (-1.1 – 0.4)	Uncertain	-0.4 (-1.1 – 0.1)	Uncertain
**Central**	Dakar	Dakar	Any ORS	1.5 (-0.4 – 3.6)	Uncertain	0.1 (-2.1 – 2.3)	Uncertain	0.8 (-1.8 – 3.5)	Uncertain
	Dakar	Dakar	Only RHF	3.5 (0.5 – 6.4)	Increase	0.4 (-1.9 – 3)	Uncertain	-0.6 (-3.5 – 2.3)	Uncertain
	Dakar	Dakar	No ORT	-5.1 (-7.2 – -3)	Decrease	-0.6 (-1.6 – 0.3)	Uncertain	-0.2 (-1.1 – 0.4)	Uncertain
	Dakar	Guédiawaye	Any ORS	1.5 (-0.6 – 3.7)	Uncertain	0.1 (-2.3 – 2.6)	Uncertain	1.2 (-1.9 – 4.5)	Uncertain
	Dakar	Guédiawaye	Only RHF	3.5 (0.1 – 6.6)	Increase	0.5 (-2.5 – 3.2)	Uncertain	-1 (-4.7 – 2.4)	Uncertain
	Dakar	Guédiawaye	No ORT	-5.1 (-7.4 – -2.8)	Decrease	-0.6 (-1.7 – 0.4)	Uncertain	-0.2 (-1.2 – 0.8)	Uncertain
	Dakar	Pikine	Any ORS	1.7 (-0.1 – 3.7)	Uncertain	0.1 (-2 – 2.1)	Uncertain	1.2 (-1.6 – 4)	Uncertain
	Dakar	Pikine	Only RHF	3.4 (0.7 – 5.9)	Increase	0.4 (-2 – 2.8)	Uncertain	-1 (-4.1 – 2)	Uncertain
	Dakar	Pikine	No ORT	-5.1 (-6.9 – -3.3)	Decrease	-0.6 (-1.6 – 0.4)	Uncertain	-0.2 (-1.1 – 0.5)	Uncertain
	Dakar	Rufisque	Any ORS	2.2 (0.6 – 4)	Increase	0.4 (-1.6 – 2.3)	Uncertain	0.8 (-1.6 – 3.2)	Uncertain
	Dakar	Rufisque	Only RHF	2.9 (0.4 – 5.2)	Increase	0.3 (-1.9 – 2.6)	Uncertain	-0.4 (-3 – 2.3)	Uncertain
	Dakar	Rufisque	No ORT	-5.1 (-7 – -3.4)	Decrease	-0.7 (-2 – 0.4)	Uncertain	-0.4 (-1.4 – 0.5)	Uncertain
	Diourbel	Bambey	Any ORS	1.7 (0.4 – 3.1)	Increase	0.4 (-1.2 – 2)	Uncertain	0.5 (-1.3 – 2.4)	Uncertain
	Diourbel	Bambey	Only RHF	3.4 (1.4 – 5.2)	Increase	0 (-1.8 – 1.9)	Uncertain	-0.4 (-2.5 – 1.7)	Uncertain
	Diourbel	Bambey	No ORT	-5.1 (-6.3 – -3.9)	Decrease	-0.4 (-1.2 – 0.2)	Uncertain	-0.1 (-0.7 – 0.4)	Uncertain
	Diourbel	Diourbel	Any ORS	1.7 (0.4 – 3.1)	Increase	0.3 (-1.4 – 1.9)	Uncertain	0.4 (-1.4 – 2.3)	Uncertain
	Diourbel	Diourbel	Only RHF	3.1 (1 – 5)	Increase	0.2 (-1.5 – 2.2)	Uncertain	-0.3 (-2.4 – 1.7)	Uncertain
	Diourbel	Diourbel	No ORT	-4.8 (-6.1 – -3.5)	Decrease	-0.5 (-1.4 – 0.2)	Uncertain	-0.1 (-0.7 – 0.5)	Uncertain
	Diourbel	Mbacké	Any ORS	1.6 (0.4 – 3)	Increase	0.2 (-1.3 – 1.7)	Uncertain	0.4 (-1.4 – 2.1)	Uncertain
	Diourbel	Mbacké	Only RHF	2.9 (1 – 4.7)	Increase	0.3 (-1.5 – 2)	Uncertain	-0.2 (-2.1 – 1.7)	Uncertain
	Diourbel	Mbacké	No ORT	-4.5 (-5.8 – -3.3)	Decrease	-0.5 (-1.3 – 0.3)	Uncertain	-0.1 (-0.8 – 0.4)	Uncertain
	Fatick	Fatick	Any ORS	1.7 (0.4 – 3)	Increase	0.3 (-1.3 – 1.9)	Uncertain	0.7 (-1.2 – 2.5)	Uncertain
	Fatick	Fatick	Only RHF	3.1 (1.3 – 4.7)	Increase	0.1 (-1.6 – 1.9)	Uncertain	-0.6 (-2.6 – 1.4)	Uncertain
	Fatick	Fatick	No ORT	-4.8 (-5.9 – -3.6)	Decrease	-0.4 (-1.1 – 0.1)	Uncertain	-0.1 (-0.6 – 0.4)	Uncertain
	Fatick	Foundiougne	Any ORS	1.4 (-0.1 – 2.9)	Uncertain	0.4 (-1.3 – 2.1)	Uncertain	0.6 (-1.3 – 2.5)	Uncertain

	Fatick	Foundiougne	Only RHF	3 (1.3 – 4.8)	Increase	0 (-1.8 – 1.9)	Uncertain	-0.4 (-2.5 – 1.7)	Uncertain
	Fatick	Foundiougne	No ORT	-4.5 (-5.8 – -3.2)	Decrease	-0.5 (-1.2 – 0.1)	Uncertain	-0.2 (-0.7 – 0.3)	Uncertain
	Fatick	Gossas	Any ORS	1.5 (0.2 – 2.9)	Increase	0.2 (-1.4 – 1.7)	Uncertain	0.6 (-1.2 – 2.3)	Uncertain
	Fatick	Gossas	Only RHF	3.1 (1.2 – 5)	Increase	0.3 (-1.4 – 2.1)	Uncertain	-0.5 (-2.4 – 1.5)	Uncertain
	Fatick	Gossas	No ORT	-4.6 (-5.9 – -3.3)	Decrease	-0.5 (-1.2 – 0.1)	Uncertain	-0.1 (-0.7 – 0.4)	Uncertain
	Kaffrine	Birkilane	Any ORS	1.4 (0 – 2.8)	Uncertain	0.2 (-1.5 – 1.9)	Uncertain	0.3 (-1.6 – 2.3)	Uncertain
	Kaffrine	Birkilane	Only RHF	2.9 (0.9 – 4.8)	Increase	0.3 (-1.6 – 2.2)	Uncertain	-0.1 (-2.1 – 1.9)	Uncertain
	Kaffrine	Birkilane	No ORT	-4.3 (-5.6 – -2.9)	Decrease	-0.5 (-1.3 – 0.1)	Uncertain	-0.2 (-0.8 – 0.2)	Uncertain
	Kaffrine	Kaffrine	Any ORS	1.3 (0 – 2.7)	Increase	0.3 (-1.3 – 1.9)	Uncertain	0.3 (-1.4 – 2)	Uncertain
	Kaffrine	Kaffrine	Only RHF	3 (1.2 – 4.7)	Increase	0.3 (-1.5 – 2)	Uncertain	-0.1 (-1.9 – 1.8)	Uncertain
	Kaffrine	Kaffrine	No ORT	-4.3 (-5.6 – -3)	Decrease	-0.5 (-1.3 – 0.1)	Uncertain	-0.2 (-0.8 – 0.2)	Uncertain
	Kaffrine	Koungheul	Any ORS	1.2 (-0.1 – 2.5)	Uncertain	0.4 (-1.2 – 1.9)	Uncertain	0.4 (-1.3 – 2.1)	Uncertain
	Kaffrine	Koungheul	Only RHF	3.3 (1.5 – 5.1)	Increase	0.1 (-1.5 – 1.8)	Uncertain	-0.2 (-2 – 1.7)	Uncertain
	Kaffrine	Koungheul	No ORT	-4.5 (-5.8 – -3.2)	Decrease	-0.5 (-1.2 – 0.2)	Uncertain	-0.3 (-0.8 – 0.1)	Uncertain
	Kaffrine	Malème Hodar	Any ORS	1.3 (0.1 – 2.6)	Increase	0.2 (-1.2 – 1.7)	Uncertain	0.4 (-1.2 – 2.1)	Uncertain
	Kaffrine	Malème Hodar	Only RHF	3 (1.2 – 4.6)	Increase	0.3 (-1.4 – 2)	Uncertain	-0.2 (-2 – 1.6)	Uncertain
	Kaffrine	Malème Hodar	No ORT	-4.3 (-5.5 – -3)	Decrease	-0.5 (-1.3 – 0.1)	Uncertain	-0.2 (-0.8 – 0.2)	Uncertain
	Kaolack	Guinguinéo	Any ORS	1.5 (0 – 3)	Increase	0.2 (-1.5 – 1.9)	Uncertain	0.6 (-1.2 – 2.5)	Uncertain
	Kaolack	Guinguinéo	Only RHF	3.1 (1.1 – 5)	Increase	0.3 (-1.5 – 2.2)	Uncertain	-0.5 (-2.5 – 1.6)	Uncertain
	Kaolack	Guinguinéo	No ORT	-4.6 (-5.9 – -3.2)	Decrease	-0.5 (-1.2 – 0.1)	Uncertain	-0.1 (-0.7 – 0.3)	Uncertain
	Kaolack	Kaolack	Any ORS	1.6 (0.1 – 3.2)	Increase	0.3 (-1.5 – 2)	Uncertain	0.5 (-1.4 – 2.5)	Uncertain
	Kaolack	Kaolack	Only RHF	3 (1 – 4.8)	Increase	0.2 (-1.7 – 2.2)	Uncertain	-0.4 (-2.4 – 1.8)	Uncertain
	Kaolack	Kaolack	No ORT	-4.6 (-5.9 – -3.3)	Decrease	-0.5 (-1.3 – 0.1)	Uncertain	-0.2 (-0.8 – 0.4)	Uncertain
	Kaolack	Nioro du Rip	Any ORS	1.3 (-0.2 – 2.7)	Uncertain	0.3 (-1.2 – 1.9)	Uncertain	0.2 (-1.5 – 1.9)	Uncertain
	Kaolack	Nioro du Rip	Only RHF	3.1 (1.3 – 5)	Increase	0.2 (-1.7 – 1.9)	Uncertain	0 (-1.8 – 1.9)	Uncertain
	Kaolack	Nioro du Rip	No ORT	-4.4 (-5.6 – -3.1)	Decrease	-0.5 (-1.3 – 0.1)	Uncertain	-0.3 (-0.8 – 0.2)	Uncertain
	Kolda	Kolda	Any ORS	1.4 (-0.2 – 2.9)	Uncertain	0.3 (-1.2 – 1.9)	Uncertain	0.3 (-1.6 – 2.1)	Uncertain
	Kolda	Kolda	Only RHF	3.9 (2.1 – 5.7)	Increase	0.1 (-1.7 – 1.9)	Uncertain	0 (-1.9 – 2)	Uncertain
	Kolda	Kolda	No ORT	-5.3 (-6.7 – -4)	Decrease	-0.4 (-1.1 – 0.2)	Uncertain	-0.3 (-0.8 – 0.1)	Uncertain

	Kolda	Médina Yoro Foula	Any ORS	1.1 (-0.3 – 2.6)	Uncertain	0.4 (-1.1 – 1.9)	Uncertain	0.3 (-1.5 – 1.9)	Uncertain
	Kolda	Médina Yoro Foula	Only RHF	3.7 (2 – 5.4)	Increase	0.1 (-1.7 – 1.7)	Uncertain	0 (-1.8 – 2)	Uncertain
	Kolda	Médina Yoro Foula	No ORT	-4.9 (-6.2 – -3.6)	Decrease	-0.4 (-1.1 – 0.1)	Uncertain	-0.3 (-0.8 – 0.1)	Uncertain
	Kolda	Vélingara	Any ORS	1.4 (0 – 2.9)	Increase	0.3 (-1.3 – 1.7)	Uncertain	0.4 (-1.5 – 2.1)	Uncertain
	Kolda	Vélingara	Only RHF	3.9 (1.9 – 5.7)	Increase	0 (-1.5 – 1.7)	Uncertain	-0.1 (-2.1 – 1.9)	Uncertain
	Kolda	Vélingara	No ORT	-5.3 (-6.6 – -4)	Decrease	-0.3 (-0.9 – 0.2)	Uncertain	-0.3 (-0.8 – 0.1)	Uncertain
	Kédougou	Kédougou	Any ORS	1.5 (0 – 2.9)	Increase	0.3 (-1.4 – 1.8)	Uncertain	0.4 (-1.5 – 2.2)	Uncertain
	Kédougou	Kédougou	Only RHF	3.5 (1.6 – 5.3)	Increase	0.1 (-1.6 – 2)	Uncertain	-0.1 (-2.1 – 1.9)	Uncertain
	Kédougou	Kédougou	No ORT	-4.9 (-6.4 – -3.5)	Decrease	-0.4 (-1 – 0.1)	Uncertain	-0.3 (-0.7 – 0.1)	Uncertain
	Kédougou	Salémata	Any ORS	1.5 (-0.1 – 2.9)	Uncertain	0.3 (-1.3 – 2)	Uncertain	0.4 (-1.8 – 2.3)	Uncertain
	Kédougou	Salémata	Only RHF	3.5 (1.4 – 5.5)	Increase	0.1 (-1.8 – 1.9)	Uncertain	-0.1 (-2.1 – 2.1)	Uncertain
	Kédougou	Salémata	No ORT	-5 (-6.6 – -3.5)	Decrease	-0.4 (-1.1 – 0.2)	Uncertain	-0.3 (-0.8 – 0.1)	Uncertain
	Kédougou	Saraya	Any ORS	1.4 (0 – 2.7)	Increase	0.3 (-1.2 – 1.7)	Uncertain	0.4 (-1.4 – 2.2)	Uncertain
	Kédougou	Saraya	Only RHF	3.5 (1.8 – 5.4)	Increase	0.1 (-1.5 – 1.7)	Uncertain	-0.1 (-2 – 1.8)	Uncertain
	Kédougou	Saraya	No ORT	-4.9 (-6.3 – -3.6)	Decrease	-0.4 (-1.1 – 0.2)	Uncertain	-0.3 (-0.8 – 0.1)	Uncertain
	Tambacounda	Bakel	Any ORS	1.4 (0.3 – 2.6)	Increase	0.2 (-1.2 – 1.6)	Uncertain	0.5 (-1 – 2.1)	Uncertain
	Tambacounda	Bakel	Only RHF	3.4 (1.8 – 5)	Increase	0.1 (-1.3 – 1.7)	Uncertain	-0.3 (-2 – 1.4)	Uncertain
	Tambacounda	Bakel	No ORT	-4.8 (-6 – -3.6)	Decrease	-0.4 (-1 – 0.1)	Uncertain	-0.3 (-0.7 – 0.1)	Uncertain
	Tambacounda	Goudiry	Any ORS	1.2 (0.1 – 2.4)	Increase	0.3 (-0.9 – 1.5)	Uncertain	0.5 (-1 – 1.9)	Uncertain
	Tambacounda	Goudiry	Only RHF	3.8 (2.2 – 5.4)	Increase	0.1 (-1.3 – 1.5)	Uncertain	-0.2 (-1.8 – 1.4)	Uncertain
	Tambacounda	Goudiry	No ORT	-5 (-6.2 – -3.9)	Decrease	-0.3 (-0.9 – 0.1)	Uncertain	-0.3 (-0.7 – 0.1)	Uncertain
	Tambacounda	Koupentoum	Any ORS	1.1 (-0.2 – 2.4)	Uncertain	0.4 (-1 – 1.8)	Uncertain	0.5 (-1.2 – 2.2)	Uncertain
	Tambacounda	Koupentoum	Only RHF	3.7 (1.9 – 5.3)	Increase	0.1 (-1.5 – 1.6)	Uncertain	-0.3 (-2.1 – 1.5)	Uncertain
	Tambacounda	Koupentoum	No ORT	-4.8 (-6.1 – -3.6)	Decrease	-0.4 (-1.1 – 0.1)	Uncertain	-0.2 (-0.7 – 0.1)	Uncertain
	Tambacounda	Tambacounda	Any ORS	1.3 (0.1 – 2.5)	Increase	0.3 (-1 – 1.5)	Uncertain	0.5 (-1.1 – 1.9)	Uncertain
	Tambacounda	Tambacounda	Only RHF	3.8 (2.2 – 5.4)	Increase	0.1 (-1.3 – 1.4)	Uncertain	-0.2 (-1.8 – 1.5)	Uncertain
	Tambacounda	Tambacounda	No ORT	-5.1 (-6.3 – -4)	Decrease	-0.3 (-0.9 – 0.1)	Uncertain	-0.3 (-0.7 – 0.1)	Uncertain
	Thiès	Mbour	Any ORS	1.6 (0.3 – 3.1)	Increase	0.3 (-1.4 – 2)	Uncertain	0.7 (-1.2 – 2.6)	Uncertain
	Thiès	Mbour	Only RHF	3.5 (1.7 – 5.4)	Increase	0.2 (-1.7 – 2)	Uncertain	-0.6 (-2.7 – 1.5)	Uncertain

	Thiès	Mbour	No ORT	-5.2 (-6.4 – -4)	Decrease	-0.5 (-1.2 – 0.2)	Uncertain	-0.2 (-0.8 – 0.3)	Uncertain
	Thiès	Thiès	Any ORS	1.7 (0.4 – 3.2)	Increase	0.4 (-1.2 – 2.1)	Uncertain	0.6 (-1.4 – 2.6)	Uncertain
	Thiès	Thiès	Only RHF	3.5 (1.5 – 5.4)	Increase	0.1 (-1.8 – 2)	Uncertain	-0.4 (-2.6 – 1.9)	Uncertain
	Thiès	Thiès	No ORT	-5.2 (-6.6 – -4)	Decrease	-0.5 (-1.3 – 0.3)	Uncertain	-0.2 (-0.8 – 0.4)	Uncertain
	Thiès	Tivaouane	Any ORS	1.8 (0.5 – 3.1)	Increase	0.4 (-1.1 – 2)	Uncertain	0.3 (-1.6 – 2.1)	Uncertain
	Thiès	Tivaouane	Only RHF	2.9 (1 – 4.6)	Increase	0 (-1.7 – 1.8)	Uncertain	-0.1 (-2.1 – 1.9)	Uncertain
	Thiès	Tivaouane	No ORT	-4.7 (-5.9 – -3.4)	Decrease	-0.4 (-1.1 – 0.2)	Uncertain	-0.2 (-0.8 – 0.2)	Uncertain
**Southern**	Sédhiou	Bounkiling	Any ORS	0.9 (-0.6 – 2.4)	Uncertain	0.4 (-1.1 – 2.2)	Uncertain	0.3 (-1.5 – 2.1)	Uncertain
	Sédhiou	Bounkiling	Only RHF	3.8 (1.9 – 5.8)	Increase	0.2 (-1.8 – 1.9)	Uncertain	0 (-1.9 – 1.9)	Uncertain
	Sédhiou	Bounkiling	No ORT	-4.7 (-6 – -3.3)	Decrease	-0.6 (-1.4 – 0)	Decrease	-0.3 (-0.7 – 0.1)	Uncertain
	Sédhiou	Goudomp	Any ORS	1 (-0.4 – 2.4)	Uncertain	0.5 (-1 – 2.2)	Uncertain	0.4 (-1.7 – 2.2)	Uncertain
	Sédhiou	Goudomp	Only RHF	4.1 (1.9 – 6)	Increase	0.1 (-1.8 – 1.8)	Uncertain	-0.1 (-2.1 – 2.1)	Uncertain
	Sédhiou	Goudomp	No ORT	-5.1 (-6.6 – -3.6)	Decrease	-0.6 (-1.4 – 0)	Uncertain	-0.3 (-0.8 – 0.1)	Uncertain
	Sédhiou	Sédhiou	Any ORS	1 (-0.4 – 2.4)	Uncertain	0.4 (-1.1 – 2.1)	Uncertain	0.4 (-1.4 – 2.2)	Uncertain
	Sédhiou	Sédhiou	Only RHF	4 (2 – 5.9)	Increase	0.2 (-1.6 – 2)	Uncertain	-0.1 (-2 – 1.9)	Uncertain
	Sédhiou	Sédhiou	No ORT	-5 (-6.4 – -3.6)	Decrease	-0.7 (-1.4 – 0)	Decrease	-0.3 (-0.7 – 0.1)	Uncertain
	Ziguinchor	Bignona	Any ORS	0.7 (-0.8 – 2.3)	Uncertain	0.4 (-1.3 – 2.1)	Uncertain	0.5 (-1.3 – 2.4)	Uncertain
	Ziguinchor	Bignona	Only RHF	4 (2.1 – 5.8)	Increase	0.2 (-1.7 – 2.1)	Uncertain	-0.2 (-2.2 – 1.8)	Uncertain
	Ziguinchor	Bignona	No ORT	-4.8 (-6.1 – -3.4)	Decrease	-0.6 (-1.4 – 0)	Uncertain	-0.3 (-0.8 – 0.1)	Uncertain
	Ziguinchor	Oussouye	Any ORS	0.8 (-1 – 2.5)	Uncertain	0.5 (-1.4 – 2.5)	Uncertain	0.6 (-1.6 – 2.9)	Uncertain
	Ziguinchor	Oussouye	Only RHF	4 (1.8 – 6.2)	Increase	-0.1 (-2.3 – 2.1)	Uncertain	-0.3 (-2.6 – 2.2)	Uncertain
	Ziguinchor	Oussouye	No ORT	-4.8 (-6.4 – -3.2)	Decrease	-0.5 (-1.4 – 0.2)	Uncertain	-0.4 (-1 – 0.2)	Uncertain
	Ziguinchor	Ziguinchor	Any ORS	0.7 (-1 – 2.4)	Uncertain	0.6 (-1.2 – 2.6)	Uncertain	0.7 (-1.5 – 2.8)	Uncertain
	Ziguinchor	Ziguinchor	Only RHF	4.2 (2 – 6.3)	Increase	0 (-2.1 – 2.1)	Uncertain	-0.3 (-2.7 – 2.1)	Uncertain
	Ziguinchor	Ziguinchor	No ORT	-4.9 (-6.5 – -3.4)	Decrease	-0.6 (-1.5 – 0.1)	Uncertain	-0.3 (-1 – 0.2)	Uncertain

8.0 Additional file 1: References

1. Munos MK, Walker CLF, Black RE. The effect of oral rehydration solution and recommended home fluids on diarrhoea mortality. Int J Epidemiol. 2010 Apr 1;39(Suppl 1):i75–87.

2. Wiens KE, Lindstedt PA, Blacker BF, Johnson KB, Baumann MM, Schaeffer LE, et al. Mapping geographical inequalities in oral rehydration therapy coverage in low-income and middle-income countries, 2000–17. Lancet Glob Health. 2020 Aug 1;8(8):e1038–60.

3. Kish L. Survey sampling. New York: John Wiley & Sons, Inc.; 1965. 672 p.

4. WorldPop. Get data. pop_release 2019_08_29. [Internet]. [cited 2017 Jul 25]. Available from: http://www.worldpop.org.uk/data/get_data/

5. Fullman N, Yearwood J, Abay SM, Abbafati C, Abd-Allah F, Abdela J, et al. Measuring performance on the Healthcare Access and Quality Index for 195 countries and territories and selected subnational locations: a systematic analysis from the Global Burden of Disease Study 2016. Lancet. 2018 Jun 2;391(10136):2236–71.

6. Faraway JJ. Linear Models with R, Chapter 4, Problems with the Predictors. CRC Press; 2004. 83–94 p.

7. Database of Global Administrative Areas. GADM data version 3.6. Shapefile version 2019_09_10. [Internet]. [cited 2019 Sep 10]. Available from: https://gadm.org/data.html

8. Bhatt S, Cameron E, Flaxman SR, Weiss DJ, Smith DL, Gething PW. Improved prediction accuracy for disease risk mapping using Gaussian process stacked generalization. J R Soc Interface. 2017 Sep 1;14(134):20170520.

9. Head T, MechCoder, Louppe G, Shcherbatyi I, fcharras, Vinícius Z, et al. scikit-optimize/scikit-optimize: v0.5.2 [Internet]. Zenodo; 2018. Available from: https://zenodo.org/record/1207017#.XMiMN2hKiUk

10. Stein ML. Interpolation of Spatial Data - Some Theory for Kriging [Internet]. Springer; 1999. Available from: https://www.springer.com/us/book/9780387986296

11. Gelfand AE, Diggle PJ, Guttorp P, Fuentes M. Handbook of Spatial Statistics [Internet]. CRC Press; 2010. Available from: https://www.crcpress.com/Handbook-of-Spatial-Statistics/Gelfand-Diggle-Guttorp-Fuentes/p/book/9781420072877

12. Rue H, Martino S, Chopin N. Approximate Bayesian inference for latent Gaussian models by using integrated nested Laplace approximations. J R Stat Soc Ser B Stat Methodol. 2009 Apr 1;71(2):319–92.

13. Martins TG, Simpson D, Lindgren F, Rue H. Bayesian computing with INLA: new features. CSDA. 2013 Nov 1;67:68–83.

14. Lindgren F, Rue H, Lindström J. An explicit link between Gaussian fields and Gaussian Markov random fields: the stochastic partial differential equation approach. J R Stat Soc Ser B Stat Methodol. 2011 Sep 1;73(4):423–98.

15. Fuglstad G-A, Simpson D, Lindgren F, Rue H. Constructing Priors that Penalize the Complexity of Gaussian Random Fields. J Am Stat Assoc. 2019 Jan 2;114(525):445–52.

16. Dwyer-Lindgren L, Cork MA, Sligar A, Steuben KM, Wilson KF, Provost NR, et al. Mapping HIV prevalence in sub-Saharan Africa between 2000 and 2017. Nature. 2019 Jun;570(7760):189–93.

17. Guerin PJ, Brasher C, Baron E, Mic D, Grimont F, Ryan M, et al. Case management of a multidrug-resistant Shigella dysenteriae serotype 1 outbreak in a crisis context in Sierra Leone, 1999-2000. Trans R Soc Trop Med Hyg. 2004 Nov;98(11):635–43.

18. Wilson SE, Morris SS, Gilbert SS, Mosites E, Hackleman R, Weum KLM, et al. Scaling up access to oral rehydration solution for diarrhea: Learning from historical experience in low– and high–performing countries. J Glob Health [Internet]. 2013 Jun [cited 2018 Oct 11];3(1). Available from: https://www.ncbi.nlm.nih.gov/pmc/articles/PMC3700030/

19. Diaz T, George AS, Rao SR, Bangura PS, Baimba JB, McMahon SA, et al. Healthcare seeking for diarrhoea, malaria and pneumonia among children in four poor rural districts in Sierra Leone in the context of free health care: results of a cross-sectional survey. BMC Public Health. 2013 Feb 20;13(1):157.

20. Carvajal-Vélez L, Amouzou A, Perin J, Maïga A, Tarekegn H, Akinyemi A, et al. Diarrhea management in children under five in sub-Saharan Africa: does the source of care matter? A Countdown analysis. BMC Public Health. 2016 Aug 19;16(1):830.

21. Maxmen A. Sierra Leone’s free health-care initiative: work in progress. The Lancet. 2013 Jan 19;381(9862):191–2.

22. Groen RS, Kamara TB, Nwomeh BC, Daoh KS, Kushner AL. Free health care for under 5 year olds increases access to surgical care in Sierra Leone: an analysis of case load and patient characteristics. World J Surg. 2013 Jun 1;37(6):1216–9.

23. McMahon SA, George AS, Yumkella F, Diaz T. Spoiled breast milk and bad water; local understandings of diarrhea causes and prevention in rural Sierra Leone. BMC Public Health. 2013 Dec 13;13(1):1172.

24. Sierra Leone Ministry of Health and Sanitation. Policy for Community Health Workers in Sierra Leone; [Internet]. 2012 [cited 2019 Nov 13]. Available from: http://chwcentral.org/sites/default/files/CHW-Policy%20Sierra%20Leone.pdf

25. Abdelmalak MJ, Ahmed BS, Mehta K. Health knowledge and health practices in Makeni, Sierra Leone: a community-based household survey. Int Health. 2016;8(3):220–6.

26. Blacklock A, Sesay A, Kamara A, Kamara M, Blacklock C. Characteristics and clinical management of patients admitted to cholera wards in a regional referral hospital during the 2012 epidemic in Sierra Leone. Glob Health Action. 2015;8:25266.

27. Camacho A, Kucharski A, Aki-Sawyerr Y, White MA, Flasche S, Baguelin M, et al. Temporal changes in Ebola transmission in Sierra Leone and implications for control requirements: a real-time modelling study. PLOS Curr Outbreaks [Internet]. 2015 Feb 10 [cited 2019 Dec 16]; Available from: index.html%3Fp=55052.html

28. Elston JWT, Moosa AJ, Moses FL, Walker G, Dotta N, Waldman R, et al. Impact of the Ebola outbreak on health systems and population health in Sierra Leone. J Public Health. 2015;

29. Winch PJ, Gilroy KE, Doumbia S, Patterson AE, Daou Z, Diawara A, et al. Operational issues and trends associated with the pilot introduction of zinc for childhood diarrhoea in Bougouni District, Mali. J Health Popul Nutr. 2008 Jun;26(2):151–63.

30. Ellis AA, Winch P, Daou Z, Gilroy KE, Swedberg E. Home management of childhood diarrhoea in southern Mali—Implications for the introduction of zinc treatment. Soc Sci Med. 2007 Feb 1;64(3):701–12.

31. Franco LM, Diop FP, Burgert CR, Kelley AG, Makinen M, Simpara CHT. Effects of mutual health organizations on use of priority health-care services in urban and rural Mali: a case-control study. Bull World Health Organ. 2008 Nov;86(11):830–8.

32. Gill S, Haynes J, Coates S. Diarrhoea dialogues from policies to progress. A call for urgent action to prevent the biggest killer of children in sub-Saharan Africa. [Internet]. [cited 2019 Nov 13]. Available from: http://defeatdd.org/sites/default/files/node-images/DD%20low%20res%20US%20Version%20J.pdf

33. Gibson A, Taylor P, Levine R, Fleming E, Mwebesa W, Waltensperger K, et al. MCHIP End-of-Project Report. :9.

34. The roots of Mali's conflict [Internet]. [cited 2019 Nov 13]. Available from: https://www.clingendael.org/pub/2015/the_roots_of_malis_conflict/2_rebellion_and_fragmentation_in_northern_mali/

35. Mali announces far-reaching health reform - The Lancet [Internet]. [cited 2020 Jan 10]. Available from: https://www.thelancet.com/journals/lancet/article/PIIS0140-6736(19)30684-1/fulltext

36. Integrated community case management of childhood illness: documentation of best practices and bottlenecks to program implementation in Senegal (summary report) [Internet]. MCHIP. 2012 [cited 2019 Nov 12]. Available from: https://www.mchip.net/technical-resource/integrated-community-case-management-of-childhood-illness-documentation-of-best-practices-and-bottlenecks-to-program-implementation-in-senegal-summary-report/

37. Wilson SE, Morris SS, Gilbert S. ORS case study: Senegal, Bill & Melinda Gates Foundation [Internet]. 2012 [cited 2020 Jan 14]. Available from: https://www.shopsplusproject.org/sites/default/files/resources/Senegal_BMGF_ORS%20Case%20Study_2012.pdf

38. Collins D, Jarrah Z, Gilmartin C, Saya U. The costs of integrated community case management (iCCM) programs: a multi–country analysis. J Glob Health [Internet]. 2014 Dec [cited 2019 Nov 12];4(2). Available from: https://www.ncbi.nlm.nih.gov/pmc/articles/PMC4267093/

39. Plan de passage a l'echelle de la prise en charge de la diarrhée chez l'enfant par le sel de réhydratation orale (SRO) a faible osmolarité et le zinc. Direction de la Santé, Division de l'Alimentation et de la Santé de l'Enfant (DANSE), Ministère de la Santé de l'Hygiène Publique et de la Prévention, Sénégal; 2011.

40. The Micronutrient Initiative, Government of Canada and Teck launch new initiative with Senegal Ministry of Health to save children's lives - Nutrition International [Internet]. [cited 2019 Oct 29]. Available from: https://www.nutritionintl.org/2012/05/micronutrient-initiative-government-canada-teck-launch-new-initiative-senegal-ministry-health-save-childrens-lives/

41. Laying the groundwork for universal health coverage in Senegal through sustainable financing | Results for Development [Internet]. [cited 2020 Jan 10]. Available from: https://www.r4d.org/projects/laying-groundwork-universal-health-coverage-senegal-sustainable-financing/

42. Reiner RC, Wiens KE, Deshpande A, Baumann MM, Lindstedt PA, Blacker BF, et al. Mapping geographical inequalities in childhood diarrhoeal morbidity and mortality in low-income and middle-income countries, 2000–17: analysis for the Global Burden of Disease Study 2017. The Lancet. 2020 Jun 6;395(10239):1779–801.
